# Supplementary material for: A patient–clinician James Lind Alliance partnership to identify research priorities for hyperemesis gravidarum
Source: BMJ Open. 2021 Jan 15;11(1):e041254. doi: 10.1136/bmjopen-2020-041254 (PMC7813320; doi:10.1136/bmjopen-2020-041254)
Supplement: Supplementary data [file bmjopen-2020-041254supp001.pdf]

Supplementary File 1:

Members of the HG PSP Steering Committee

**Patient Representatives**	Margaret O'Hara (MOH)	Pregnancy Sickness Support (PSS) (United Kingdom(UK) charity)
	Emma Watford (EW)	Pregnancy Sickness Support (PSS) (United Kingdom(UK) charity)
	Norah Gauw (NGT)	ZEHG, The Netherlands
	Karina Fee (KF)	Hyperemesis Ireland
	Kimber MacGibbon (KMG)	Hyperemesis Education and Research (HER) Foundation (United States (US) and international charity)
**Clinical representatives**	Dr Rebecca Painter (RP) (Ob/Gyne)	Amsterdam University Medical Centres (and ZEHG Charity)
	Prof Catherine Nelson-Piercy (C N-P) (Obstetric Physician)	Professor of obstetric medicine and consultant obstetric physician, Guy's and St Thomas' Hospital
	Prof Brian Cleary (BC)(Pharmacist)	Rotunda Hospital, Dublin (and Hyperemesis Ireland Charity)
	Marian MacBride (MM) (Senior Dietician)	Health Service Executive, Dublin (and Hyperemesis Ireland Charity)
	Katherine Shorter (KS) (Nurse, HG Day Unit)	Nottingham University Hospital Trust
	Deirdre Munro (DM) (Midwife)	Global Village of Midwives Network, Portiuncula University Hospital, Co. Galway Ireland (and Hyperemesis Ireland Charity)
	Dr Roger Gadsby (RG) (General Practitioner)	Royal College of General Practitioners + experience of running a JLA PSP and PSS UK Charity
	Prof Jone Trovik (JT) (Ob/Gyne)	Haukeland University Hospital, Bergen, Norway
	Dr Ria Clarke (RC) (Ob/Gyne)	Junior Doctor, UK
	Dr Helen Penny (HP) (Clinical Psychologist)	Cardiff University
**The James Lind Alliance (JLA)**	Patricia Ellis (PE)	James Lind Alliance
