# Supplementary material for: A patient–clinician James Lind Alliance partnership to identify research priorities for hyperemesis gravidarum
Source: BMJ Open. 2021 Jan 15;11(1):e041254. doi: 10.1136/bmjopen-2020-041254 (PMC7813320; doi:10.1136/bmjopen-2020-041254)
Supplement: Supplementary data [file bmjopen-2020-041254supp002.pdf]

A JAMES LIND ALLIANCE PROJECT

HYPEREMESIS GRAVIDARUM PRIORITY SETTING PARTNERSHIP - PROTOCOL

PROTOCOL DRAFT 27/02/2018

PURPOSE OF THE PSP AND BACKGROUND

The purpose of this protocol is to set out the aims, objectives and commitments of the Hyperemesis Gravidarum (HG) Priority Setting Partnership (PSP) (HG PSP) and the basic roles and responsibilities of the partners therein. It is recommended that the Protocol is reviewed by the Steering Group and updated on at least a quarterly basis.

The James Lind Alliance (JLA) is a non-profit initiative, established in 2004. It brings patients, carers and clinicians together in Priority Setting Partnerships (PSPs). These partnerships identify and prioritise uncertainties, or 'unanswered questions', about the effects of treatments or other aspects of the condition, such as diagnosis, prognosis and the organisation of care, that they agree are the most important. The aim of this is to help ensure that those who fund health research are aware of what really matters to both patients and clinicians. The National Institute for Health Research (NIHR – www.nihr.ac.uk) funds the infrastructure of the JLA to oversee the processes for priority setting partnerships, based at the NIHR Evaluation, Trials and Studies Coordinating Centre (NETSCC), University of Southampton.

The HG PSP has come together following the 2nd International Colloquium on Hyperemesis Gravidarum held in the UK in October 2017. During this event leading HG researchers as well as patients from around the world agreed that setting Research Priorities for the condition was itself a priority and motivation for such a project was strong. Following this event, a subgroup of speakers and delegates with established international links to others in the field agreed to pursue the project and formed the initial PSP.

Historically, HG has been an under-researched condition and the quality of HG research to date has been poor (Boelig et al. 2016; McParlin et al. 2016). In part efforts have been hampered by a lack of international definition of HG and core outcome set for research, thus rendering much of the data too heterogenetic for meta-analysis

(Grooten et al. 2015). Thankfully these barriers are being tackled and an internationally agreed definition and core outcome set are anticipated in 2018 (ref conference).

A Cochrane review in 2016 (Boelig et al. 2016) concluded that there was insufficient evidence to provide a definitive recommendation on the best way to treat severe nausea and vomiting in early pregnancy. A simultaneous systematic review concluded that the overall quality of evidence for HG treatments was low and that further research was therefore required (O'Donnell et al. 2016). In particular the authors of the review made a number of research recommendations in order of priority. However, neither of these systematic reviews engaged meaningfully with patients who have experienced hyperemesis gravidarum and as a result the research recommendations have been criticised by patient advocacy groups for this condition (Chapman 2016) (email communication with HTA and public engagement exercises).

An increase in public and professional interest in HG in recent years and recognition of the long-term consequences for offspring has led to increase in funding and a flurry of research proposals. There is also a strong motivation among women affected by HG to take part in research and in patient and public involvement in the development of HG research (Dean and Goddard 2016).

AIMS AND OBJECTIVES OF THE HG PSP

The aim of the HG PSP is to identify the unanswered questions about HG treatment from patient and clinical perspectives and then prioritise those that patients and clinicians agree are the most important.

The objectives of the HG PSP are to:

- Together with patients and clinicians, work internationally to identify uncertainties about the effects of HG treatments and management which have not yet been answered by existing research
- Determine by international consensus a prioritised list of those uncertainties, to guide future research
- To publicise the results of the PSP among researchers, research commissioning bodies and the general public in order to stimulate research in these areas

THE STEERING GROUP

The HG PSP will be led and managed Caitlin Dean (CD) with the following steering group:

**Patient Representatives**	Margaret O'Hara (MOH)<br>Emma Watford (EW)	Pregnancy Sickness Support (PSS) (United Kingdom (UK) charity)

	Norah Gauw (NG)	The Netherlands
	Karina Fee (KF)	Hyperemesis Ireland
	Kimber MacGibbon (KMG)	Hyperemesis Education and Research (HER) Foundation (United States (US) and international charity)
**Clinical representatives**	Dr Rebecca Painter (RP)	Amsterdam Medical Centre
	Prof Catherine Nelson-Piercy (C N-P) (Obstetric Physician)	Professor of obstetric medicine and consultant obstetric physician, Guy's and St Thomas' Hospital
	Dr Brian Cleary (BC)(Pharmacist)	Rotunda Hospital, Dublin
	Marian MacBride (MM) (Dietician)	Rotunda Hospital, Dublin
	Kate Shorter (KS) (Nurse, HG Day Unit)	Nottingham University Hospital Trust
	Deirdre Munro (DM) (Midwife)	Global Village of Midwives Network
	Dr Roger Gadsby (RG) (General Practitioner)	Royal College of General Practitioners + experience of running a JLA PSP
	Jone Trovik (JT) (Ob/Gyne)	Haukeland University Hospital, Bergen, Norway
	Ria Clarke (RC) (Ob/Gyne)	Junior Doctor, UK
	Helen Penny (HP) (Psychologist)	Cardiff University
**The James Lind Alliance (JLA)**	Patricia Ellis (PE)	James Lind Alliance

Dean CR, *et al. BMJ Open* 2021; 11:e041254. doi: 10.1136/bmjopen-2020-041254

The Steering Group includes representation of patient/carer groups and clinicians[1].

The Steering Group will agree the resources, including time and expertise that they will be able to contribute to each stage of the process. The JLA will advise on this.

THE WIDER PARTNERS

Organisations and individuals will be invited to be involved with the PSP as partners. Partners are groups or individuals who will commit to supporting the PSP by disseminating the PSP survey and helping the PSP to gather questions and uncertainties of practical clinical importance relating to the treatment and management of the health problem in question. Partners represent the following groups:

- people who have had HG
- carers and offspring of people who have had HG
- medical doctors, nurses and professionals allied to medicine with clinical experience of HG.

It is important that all organisations which can reach and advocate for these groups should be invited to become involved in the PSP. The JLA Adviser will take responsibility for ensuring the various stakeholder groups are able to contribute equally to the process.

EXCLUSION CRITERIA

Some organisations may be judged by the JLA or the Steering Group to have conflicts of interest, which may be perceived to adversely affect those organisations' views, causing unacceptable bias, for example, pharmaceutical companies or individual stakeholders in such companies. As such conflicts of interest would be likely to affect the ultimate findings of the PSP, those organisations will not be invited to participate. It is possible, however, that interested parties may participate in a purely observational capacity when the Steering Group considers it may be helpful.

[1] In some cases, it has been suggested that researchers are represented at this level, to advise on the shaping of research questions. However, researchers cannot participate in the prioritisation exercise. This is to ensure that the final prioritised research questions are those agreed by patients, carers and clinicians only, in line with the JLA's mission.

THE METHODS THE PSP WILL USE

The methods adopted in any stage will be agreed through consultation between the Steering Group members, guided by the PSP's aims and objectives.

STEP 1: IDENTIFICATION AND INVITATION OF POTENTIAL PARTNERS

Potential partner organisations will be identified through a process of peer knowledge and consultation, through the Steering Group members' networks. Potential partners will be contacted and informed of the establishment and aims of the HG PSP and may be invited to participate in an online discussion.

People wishing to participate will be able to register using an online form with a convenient email address for future communication via mailchimp. Details of which stakeholder group they identify as and which organisation they represent will be gathered.

Initial organisations will include:

Patient Charities:	Pregnancy Sickness Support
	HER Foundation
	Hyperemesis Ireland
	ZEHG (Netherlands organisation)
	Hyperemesis Gravidarum Facebook Group (10,000 + members)
	Hyperemesis Gravidarum Norway
	NB. A number of countries have budding organisations and informal patient networks, such as Australia and Germany. Representatives from each country we are aware of will be contacted. Additionally, the HER Foundation has at least one contact in most countries worldwide.
Clinician Networks:	Royal College of Obstetricians and Gynaecologists
	Royal College of General Practitioners
	Royal College of Midwives
	Royal College of Nursing Midwifery Forum and Women's Health Forum
	Association of Early Pregnancy Units
	American College of Obstetricians and Gynaecologists
	Dutch NVOG

Dean CR, *et al. BMJ Open* 2021; 11:e041254. doi: 10.1136/bmjopen-2020-041254

	Dutch O&G Nurses
	"RCOG" and "RCM" equivalents for as many countries as possible will be sought out and approached.

STEP 2: INITIAL STAKEHOLDER INTRODUCTION

The initial stakeholder information sheet for those registering interest in participation will have several key objectives:

- To welcome and introduce potential members of the HG PSP
- To present the proposed plan for the PSP
- To initiate discussion, answer questions and address concerns
- To invite potential partner organisations which will commit to the PSP to identify themselves and identify individuals who will be those organisations' representatives and the PSP's principal contacts
- To establish principles upon which an open, inclusive and transparent mechanism can be based for contributing to, reporting and recording the work and progress of the PSP.

This will be done via email using the emails previously registered. A forum within the project website can be used to initiate discussion, answer questions and address concerns.

STEP 3: IDENTIFYING TREATMENT UNCERTAINTIES

Each partner will disseminate an online survey to its members to solicit questions and uncertainties of practical clinical importance relating to the treatment and management of HG. A period of 8 weeks will be given to complete this exercise. Online surveys have been found to be an effective way of engaging with women affected by HG (Dean and Goddard 2016) and for collecting research priorities (Gill et al. 2013).

Predominately treatment uncertainties will be collected using an online questionnaire however the methods may be designed according to the nature and membership of each organisation, but must be as transparent, inclusive and representative as practicable. Methods may include membership meetings, email consultation, postal questionnaires, internet message boards and focus group work.

Existing sources of information about treatment uncertainties for patients and clinicians will be searched. These can include question-answering services for patients and carers and for clinicians; research recommendations in systematic reviews and clinical guidelines; protocols for systematic reviews being prepared and registers of ongoing research. A separate protocol for this search will be written.

STEP 4: REFINING QUESTIONS AND UNCERTAINTIES

The consultation process will produce "raw" unanswered questions about diagnosis and the effects of treatments. These raw questions will be assembled, categorised and refined by CD and RP into "collated indicative questions" which are clear, addressable by research and understandable to all. Similar or duplicate questions will be combined where appropriate and the questions prepared for prioritisation. The following stages outlined by the JLA PSP Data Management Guide (James Lind Alliance 2016) will be followed:

Stage 1: Downloading the survey data

Survey responses from survey monkey will be downloaded and managed in an Excel spreadsheet. Additional uncertainties identified through other methods, ie. Literature search, discussion groups etc. will be added into the spreadsheet with their source documented. Each respondent and their demographic data will be identified with a unique identifier which will be attached to each of their submissions where one person submitted multiple responses. Personal identifying details will then be removed to anonymise the data.

Stage 2: Remove out of scope submissions

The scope of this PSP will need to be determined by the steering group in advance *(see minutes of steering group meeting 2 – scope). Submissions which fall outside of the scope or are established to not be uncertainties will be removed and kept separately with associated detail such as number of each stakeholder group that submitted the question and their demographics. These exclusions will be reviewed by the steering group to confirm the exclusion and decisions regarding what to do with them will be made (ie. Passing to other organisations or feeding back to the participants)

Stage 3: Categorising eligible submissions

Submissions will be categorised using the existing taxonomy by the UK Clinical Research Collaboration: Health Research Classification System (UK Clinical Research Collaboration 2009).

Stage 4: Format the submissions

Submissions will be rephrased to clarify the precise uncertainty. Where possible the "Patient/Population, Intervention, Comparison, Outcome" (PICO) format will be used. Where this is not possible uncertainties will be formatted in a way that will be useful and valuable to the research community. Lay language will be used.

Submissions which cannot be standardised will be listed and referred to the steering group for discussion. Where possible they may be combined with other eligible submissions. Formatted questions will be recorded with their frequency of submission by each stakeholder group (ie. How many times it was submitted by patients and clinicians).

Stage 5: Verifying the uncertainties

Dean CR, *et al. BMJ Open* 2021; 11:e041254. doi: 10.1136/bmjopen-2020-041254

Systematic reviews and guidelines will be identified and checked by CD to see to what extent these refined questions have, or have not, been answered by previous research, specifically, up-to-date systematic review or meta-analysis. Sometimes, uncertainties are expressed that can in fact be resolved with reference to existing research evidence - ie they are "unrecognised knowns" and not uncertainties. These 'answerable questions' will be recorded and dealt with separately from the 'true uncertainties' considered during the research priority setting process. If a question about treatment effects can be answered with existing information but this is not known, it suggests that information is not being communicated effectively to those who need it.

Uncertainties which are not adequately addressed by previous research will be collated and recorded on a template supplied by the JLA by CD. The list will be reviewed and agreed by the steering group.

Evidence checking protocol as agreed by Steering Group on 15th July 2019

HYPEREMESIS GRAVIDARUM PRIORITY SETTING PARTNERSHIP

EVIDENCE CHECKING PROTOCOL

This summary sets out the approach to the evidence checking process for the Hyperemesis Gravidarum Priority Setting Partnership (HG PSP), including the specific role of the Steering Group (SG) and what will be done with the findings.

THE PURPOSE OF EVIDENCE CHECKING

Systematic reviews and guidelines will be identified and checked by Caitlin Dean (CD) to see to what extent the refined questions, generated from the raw survey data, have, or have not, been answered by previous research. Sometimes, uncertainties are expressed that can in fact be resolved with reference to existing research evidence - ie they are "unrecognised knowns" and not uncertainties. If a question about treatment effects can be answered with existing information but this is not known, it suggests that information is not being communicated effectively to those who need it. Whereas uncertainties not adequately addressed by previous research will go forward to the next stage for prioritisation.

THE PROCESS FOR EVIDENCE CHECKING

Searches

A scoping search of Embase and Medline was conducted in August 2018 by information specialist Rene Spijker as part of a wider evidence mapping project running concurrently with the HG PSP. The search strategy sought to find all papers about HG without limitations. Embase, Medline were search for the key terms hyperemesis gravidarum/ or ("Excessive vomiting" or (pernicious adj3 vomiting) or hyperemesis) and (gravid* or pregn* or gestation or antenatal)). Additionally, for this evidence check, searches will cover the Cochrane Database of Systematic Reviews, the Cochrane Controlled Trials Register and trip Database for Guidelines. The NICE, RCOG and ACOG guidelines will be checked for references with a suitably high level of evidence (ie. Systematic review within 10 years).

The results will been screened and labelled as to which question number they relate to by two researchers independently.

Each question will be checked against the labelled results for systematic reviews with conclusive results which directly answer the question.

Additionally, the Cochrane Library and the RCOG and ACOG guidelines will be checked for each question for a suitably high level of evidence with a definitive recommendation/answer.

Excluding NICE

NICE guidelines will not be used for evidence checking for two reasons;

1. Statements and recommendations are not referenced in a way that can be cross checked for level of evidence.
2. The website states: *"This CKS topic is largely based on the Royal College of Obstetricians and Gynaecologists guideline: The management of nausea and vomiting of pregnancy and hyperemesis gravidarum [RCOG, 2016], the American College of Obstetricians and Gynecologists Practice Bulletin: Nausea and vomiting of pregnancy [ACOG, 2015], and review articles."* These references are already being check as stated above.

10 year inclusion

Due to the paucity of HG research and lack of funding the SG felt that a three-year limit on SRs, as recommended by the James Lind Alliance, was too limited. Therefore, SRs within the last 10 years will be deemed acceptable.

Final outcome

Dean CR, *et al. BMJ Open* 2021; 11:e041254. doi: 10.1136/bmjopen-2020-041254

Uncertainties which are not adequately addressed by previous research will be collated and recorded on a template supplied by the JLA (see Appendix A) by CD and will go onto the next stage for prioritisation.

UNKNOWN KNOWNS

Questions which are identified as already answered, but which people are unaware are already answered, ie. Unknown knowns, need to be translated into accessible information for the general public. CD will write a general article outlining the questions we have answers to and the answers themselves (not scientific) in lay language for dissemination to the general public via the main international charities, blogs and newsletters. Additionally, authors of guidelines such as the RCOG and NICE will be informed of the findings to ensure they are included in updates to the guidelines (if they are not already).

FURTHER INFORMATION

The JLA Guidebook provides step-by-step guidance to establishing and managing PSPs. You can find the Guidebook at www.jla.nihr.ac.uk

STEP 5: PRIORITISATION – INTERIM AND FINAL STAGES

The aim of the final stage of the priority setting process is to prioritise through consensus the identified uncertainties relating to the treatment or management of HG. This will be carried out by members of the Steering Group and the wider partnership that represents patients and clinicians. The stages will depend on the number of true uncertainties identified.

If there are more than 100 the Steering group will need to do an initial prioritisation to reduce the list to a more manageable number (max 80). Criteria for consideration include:

- Was the question suggested by both patients and clinicians?
- How many people suggested the question from each stakeholder group and in total?
- Was the question submitted by different kinds of clinicians or internationally?
- Whether the question overlaps with other ones or research recommendations

Interim Priority Setting

This will be conducted as an online survey. Questions will be scored and ranked by participants and the process tracked with relevant details. From here approx. 25 priorities will be retained for the final priority setting meeting.

Final Priority setting

The final stage to identify 10 prioritised uncertainties will take place at the ICHG Conference in Amsterdam in October 2019. The JLA will facilitate this process and ensure transparency, accountability and fairness. Participants will be expected to declare their interests in advance of this meeting.

DISSEMINATION OF FINDINGS AND RESEARCH

FINDINGS AND RESEARCH

The data will be submitted to the JLA for publication on its website on completion of the priority setting exercise, taking into account any changes made at the final workshop, in order to ensure that PSP results are publicly available.

It is anticipated that the findings of the HG PSP will be reported to funding and research agenda setting organisations such as the NIHR and the major research funding charities. Steering Group members and partners are expected to develop the prioritised uncertainties into research questions, and to work to establish the research needs of those unanswered questions to use when approaching potential funders, or when allocating funding for research themselves, if applicable. It is anticipated that Pregnancy Sickness Support will aim to provide modest grants for researchers addressing priority questions.

PUBLICITY

In addition to an academic paper a simple language report will be produced by the partner organisations and a PR strategy implemented.

A review of research resulting from the priority list will be presented at ICHG 2021 in Dublin and priority given at that conference to presentations and posters addressing the priority questions.

REFERENCES

Boelig RC, Barton SJ, Saccone G, Kelly AJ, Edwards SJ, Berghella V. 2016. Interventions for treating hyperemesis gravidarum. Cochrane Database Syst Rev 5:DOI: 10.1002/14651858.CD010607.pub2.

Chapman S. 2016. Hyperemesis gravidarum: sufferers need support, compassion and better research. Evidently Cochrane.

Dean C, Goddard S. 2016. Patient and Public Involvement in Designing an Online Survey about Hyperemesis Gravidarum. MIDIRS Midwifery Digest 26(3):283-290.

Gill FJ, Leslie GD, Grech C, Latour JM. 2013. Using a web-based survey tool to undertake a Delphi study: application for nurse education research. Nurse Educ Today 33(11):1322-8.

Grooten I, Roseboom T, Painter R. 2015. Barriers and Challenges in Hyperemesis Gravidarum Research. Nutr Metab Insights 8(Suppl 1):33-9.

James Lind Alliance. 2016. JLA PSP Data Management Guide. Southampton: James Lind Alliance.

McParlin C, O'Donnell A, Robson SC, Beyer F, Moloney E, Bryant A, Bradley J, Muirhead CR, Nelson-Piercy C, Newbury-Birch D et al. . 2016. Treatments for Hyperemesis Gravidarum and Nausea and Vomiting in Pregnancy: A Systematic Review. JAMA: Journal of the American Medical Association 316(13):1392-1401.

O'Donnell A, McParlin C, Robson SC, Beyer F, Moloney E, Bryant A, Bradley J, Muirhead C, Nelson-Piercy C, Newbury-Birch D et al. . 2016. Treatments for hyperemesis gravidarum and nausea and vomiting in pregnancy: a systematic review and economic assessment. Health Technol Assess 20(74):1-268.

UK Clinical Research Collaboration. Health Research Classification System [Internet]. London: UK Clinical Research Collaboration; 2009. Available from: http://www.hrcsonline.net/sites/default/files/HRCS_Document.pdf

Dean CR, *et al. BMJ Open* 2021; 11:e041254. doi: 10.1136/bmjopen-2020-041254
