# Supplementary material for: A patient–clinician James Lind Alliance partnership to identify research priorities for hyperemesis gravidarum
Source: BMJ Open. 2021 Jan 15;11(1):e041254. doi: 10.1136/bmjopen-2020-041254 (PMC7813320; doi:10.1136/bmjopen-2020-041254)
Supplement: Supplementary data [file bmjopen-2020-041254supp004.pdf]

Supplementary file 4

Data codes

Codes for types of participants

**Code**	**Demographic question response**
P	I was diagnosed with HG during pregnancy or I had HG during pregnancy but was not diagnosed
C	I cared for my partner/daughter/sister/friend when she had HG
O	My mother had HG during her pregnancy with me (offspring)
H	I am a health or social care professional caring for women with HG
ORG	I am part of an organisation representing people with HG and their families

Codes for raw questions prior to establishing indicative questions

OOS	Out of scope;<br>1 = alternative therapy<br>2 = other
C	Cause
H	Hereditability
R	Recurrence
MMA	Markers/Measurements/Assessment tool incl. clinical predictors
RF	Risk factors
DC	Disease characteristics incl. prevalence, duration, symptoms
T<br>TM<br><br><br><br><br><br><br>TG<br>TO<br>TIV<br>TN<br>TP<br>TA<br>TSO<br>TMM<br>TL<br>TT<br>TSAT	Treatment<br>Treatment - Medication;<br>1 = efficacy<br>2 = safety<br>3 = development of new ones<br>4 = combining/polypharmacy<br>5 = side effects<br>6 = administration (route/dose/timing)<br>Treatment guidelines/protocols<br>Treatments - other<br>Treatment - Intravenous fluid<br>Treatment – nutritional/nutrition<br>Treatment - Prevention<br>Treatment - Access<br>Treatment service organisation<br>Treatment – Marijuana<br>Treatment -Licensing<br>Treatment – Termination<br>Treatment - Satisfaction
S	Support<br>1 = psychological<br>2 = holistic<br>3 = family/carer
CS	Coping Strategies
CCO	Consequences, Complications, outcomes, other symptoms<br>1 = foetal risks/consequences incl. long term

	2 = maternal risks/consequences incl. long term<br>3 = mental health during pregnancy<br>4 = mental health after pregnancy<br>5 = quality of life<br>6 = for family
ED	Education<br>1 = of HCPs<br>2 = public/others
POST	Post HG, recovery, trauma
ECON	Economic questions
METH	Methodology specified
A	Attitudes/Beliefs/Stigma/Awareness
OTHER	Other (for further discussion with Steering Group)
