# Supplementary material for: A patient–clinician James Lind Alliance partnership to identify research priorities for hyperemesis gravidarum
Source: BMJ Open. 2021 Jan 15;11(1):e041254. doi: 10.1136/bmjopen-2020-041254 (PMC7813320; doi:10.1136/bmjopen-2020-041254)
Supplement: Supplementary data [file bmjopen-2020-041254supp005.pdf]

A JAMES LIND ALLIANCE PROJECT

HYPEREMESIS GRAVIDARUM PRIORITY SETTING PARTNERSHIP

EVIDENCE CHECKING PROTOCOL

This summary sets out the approach to the evidence checking process for the Hyperemesis Gravidarum Priority Setting Partnership (HG PSP), including the specific role of the Steering Group (SG) and what will be done with the findings.

THE PURPOSE OF EVIDENCE CHECKING

Systematic reviews and guidelines will be identified and checked by Caitlin Dean (CD) to see to what extent the refined questions, generated from the raw survey data, have, or have not, been answered by previous research. Sometimes, uncertainties are expressed that can in fact be resolved with reference to existing research evidence - ie they are "unrecognised knowns" and not uncertainties. If a question about treatment effects can be answered with existing information but this is not known, it suggests that information is not being communicated effectively to those who need it. Whereas uncertainties not adequately addressed by previous research will go forward to the next stage for prioritisation.

THE PROCESS FOR EVIDENCE CHECKING

Searches

A scoping search of Embase and Medline will be conducted by an information specialist (René Spijker as part of a wider evidence mapping project running concurrently with the HG PSP. The search strategy will seek to find all papers about HG without limitations. Embase, Medline will be searched for the key terms hyperemesis gravidarum/ or (("Excessive vomiting" or (pernicious adj3 vomiting) or hyperemesis) and (gravid* or pregn* or gestation or antenatal)). Additionally, for this evidence check, searches will cover the Cochrane Database of Systematic Reviews, the Cochrane Controlled Trials Register and trip Database for Guidelines. The RCOG and ACOG guidelines will be checked for references with a suitably high level of evidence (ie. Systematic review within 10 years) as per the JLA methodology handbook.

The results will be screened and labelled as to which question number they relate to by two researchers independently.

Each question will be checked against the labelled results for systematic reviews with conclusive results which directly answer the question.

Additionally, the Cochrane Library and the RCOG and ACOG guidelines will be checked for each question for a suitably high level of evidence with a definitive recommendation/answer.

Excluding NICE

NICE guidelines will not be used for evidence checking for two reasons;

1. Statements and recommendations are not referenced in a way that can be cross checked for level of evidence.
2. The website states: *"This CKS topic is largely based on the Royal College of Obstetricians and Gynaecologists guideline: The management of nausea and vomiting of pregnancy and hyperemesis gravidarum [RCOG, 2016], the American College of Obstetricians and Gynecologists Practice Bulletin: Nausea and vomiting of pregnancy [ACOG, 2015], and review articles."* These references are already being check as stated above.

10 year inclusion

Due to the paucity of HG research and lack of funding, the SG felt that a three-year limit on SRs, as recommended by the James Lind Alliance, was too limited. Therefore, SRs within the last 10 years will be deemed acceptable.

Final outcome

Uncertainties which are not adequately addressed by previous research will be collated and recorded on a template supplied by the JLA (see Appendix A) by CD and will go onto the next stage for prioritisation.

UNKNOWN KNOWNS

Questions which are identified as already answered 'Unknown knowns', need to be translated into accessible information for the general public. CD will write a general article outlining the questions we have answers to and the answers themselves (not scientific) in lay language for dissemination to the general public via the main international charities, blogs and newsletters. Additionally, authors of guidelines such as the RCOG and NICE will be informed of the findings to ensure they are included in updates to the guidelines (if they are not already).

FURTHER INFORMATION

The JLA Guidebook provides step-by-step guidance to establishing and managing PSPs. You can find the Guidebook at www.jla.nihr.ac.uk
