# Supplementary material for: A patient–clinician James Lind Alliance partnership to identify research priorities for hyperemesis gravidarum
Source: BMJ Open. 2021 Jan 15;11(1):e041254. doi: 10.1136/bmjopen-2020-041254 (PMC7813320; doi:10.1136/bmjopen-2020-041254)
Supplement: Supplementary data [file bmjopen-2020-041254supp006.pdf]

Supplementary File 6: Questions, evidence, participant categories of submissions and examples

Question rank after first prioritisation	Question code	Uncertainty	Answered?	No of refs identified from search strategy	Evidence	Total sub-missions	patients	HCPs	Carers	Off-spring	Org reps	Unkn-own	Sample of submitted questions (sic)
1	1	What causes hyperemesis gravidarum?	No	62	Various papers with new research in recent years. No definitive answer or SR yet.	282	249	28	9	31	1	14	Where does HG come from? What causes HG to begin with and why does it only affect some women? What medical studies is have been conducted to find the cause / cure for HG? Why do we get hg? What is the pathophysiology behind hyperemesis gravidarum? Why do some women get HG snd others don't? Why does one person get HG and the other don't?
11	2	What are the risk factors for developing HG and can we predict who will develop it?	No	85	Fan, L. Y.; Jacobsen, K. H. 2010 Risk factors for hyperemesis gravidarum, Current Women's Health Reviews - Volume 6, Issue 4, pp. 309-317: systematic review from 2010 identifies a few risk factors; Low pre-pregnancy weight, Helicobacter pylori infection, a history of hyperemesis gravidarum in a previous pregnancy, and carrying a female fetus but calls for further research with better definition of maternal characteristics.	96	77	21	4	12	1	7	Are there any ways to predict who will develop HG? As they learn more about HG, to learn if women could be tested thru gene testing. Is there a test being developed to determine likelihood off suffering HG? Can we use genetic testing to identify women as risk and offer preventative meds earlier? And will this lessen the severity? Is there any way of predicting if you will suffer from this?
3	3	What is the risk that HG will reoccur in a subsequent pregnancy? Does HG get progressively worse with subsequent pregnancies and what are the risk factors for recurrence?	No	8	5 papers vary from 15-81%, meta-analysis not possible.	212	191	16	10	17	0	6	What is the recurrence rate? Will I definitely have HG again in future pregnancies? Does Hyperemesis get worse with each pregnancy? What are the chances that you get HG with the following pregnancy/pregnancies? If you have HG, how likely are you to get it a second time? What are the chances of getting HG again after suffering with it during my first pregnancy? Is HG more severe dependant on partner? Are you likely to get HG again if you use sperm from a different man/donor? How much of an influence does the sperm have?
40	4	What clinical measurements and markers are most useful in assessing, diagnosing, managing and monitoring hyperemesis?	No	47	Niemeijer, M. N.; Grooten, I. J.; Vos, N.; Bais, J. M.; van der Post, J. A.; Mol, B. W.; Roseboom, T. J.; Leeflang, M. M.; Painter, R. C.; 2014 Diagnostic markers for hyperemesis gravidarum: a systematic review and metaanalysis American Journal of Obstetrics & Gynecology - Volume 211, Issue 2, pp. 150.e1-150.15: Systematic review concludes no support for the use of ketonuria in the diagnosis of HG. H	11	7	3	0	1	0	1	What anthropometric measures and nutritonal markers are most useful in assessing and monitoring hyperemesis management? (??weight, mid upper arm circumference, transferrin, amylase etc?????) What clinical indicators determine the threshold for hospital admission in women with HG? What diagnostic criteria/guidelines would provide the best treatment/outcomes? i.e. instead of waiting for significant weight loss and dehydration or "penalizing"proactive care that reduces these indicators

					pylori serology might be useful in specific patients.								
46	5	What objective measures can people used to know when to seek further help or hospital admission?	No	0	None	7	6	1	0	0	0	0	Self monitoring urine to know when to need help.... is this a possibility? Is there a way for the individual to self monitor how bad things are getting to be able to be proactive? How do you know when you need to go to hospital, eg what are the measures of dehydration? I remember going to hospital and being told you are not dehydrated enough for treatment following that I was reluctant to go to hospital
39	6	Can we predict the severity and duration for individual patients and which treatments they will respond to?	No	7	Some original studies identified but no systematic review or conclusive evidence	12	7	5	0	0	0	0	What factors predict severity and duration of HG in a pregnancy? Are there any clinical predictors of response to different anti-emetics in HG? Why do some HG sufferers have relief of symptoms afte 3/4 months and some are sick each day until the day they give birth? Can we predict how long HG will last? Is there any way of predicting which medications are most effective for whom?
	7	Does the sex of the foetus affect occurrence and/or severity of HG?	Yes	8	increased ratio of female to male fetus in HG pregnancies. (OR 1.27; 95% CI 1.21–1.34): MVE Veenendaal AFM van Abeelen RC Painter JAM van der Post TJ Roseboom, 2011, Consequences of hyperemesis gravidarum for offspring: a systematic review and meta-analysis. BJOG 18(11)	21	19	2	1	3	0	1	What are the chances my HG will be worse with a girl pregnancy than with a boy? Does the sex of the baby (boy/girl) make a difference in the extent of complaints of HG? Is there A difference in HG Between women who are Carrying girls vs boys?
29	8	Does Ketoanalysis have a role in the diagnosis and management of HG?	Yes and no	3	Ketoanalysis does not corrlate with severity of HG and should therefore not be used for diagnosis of HG however, this does not answer whether or not they have a role in management of HG. Niemeijer, M. N.; Grooten, I. J.; Vos, N.; Bais, J. M.; van der Post, J. A.; Mol, B. W.; Roseboom, T. J.; Leeflang, M. M.; Painter, R. C.; 2014 American Journal of Obstetrics & Gynecology - Volume 211, Issue 2, pp. 150.e1-150.15: Systematic review concludes no support for the use of ketonuria in the diagnosis of HG. Guidelines conflict but based on "good practice point/clinical experience"	21	18	3	1	1	0	0	Why do we rely on ketones to test dehydration? Can treatments be provided before you have ketones in urine instead of after? Will hospitals realise they do not have to look at ketones to diagnose dehydration in a hg patient? Why is ketosis the measure for dehydration? Can I still be dehydrated without having ketones in my urine? Why do women have to get to the point of having ketones before their condition is treated seriously?

18	9	What should the clinical definition of HG be and can it be diagnosed in a timely and consistent way?	No	1	Guidelines give level D evidence	56	51	3	0	7	0	4	At what point is your morning sickness classed as HG and is there different levels of HG ie Mild/Severe? How can we diagnose and treat patients quicker? Can hg be detected earlier in pregnancy? How can we identify hyperemesis earlier in pregnancy? What are the criteria of HG? How can Health Care Professional assess and diagnose HG earlier? How can HG be quickly diagnosed (and not dismissed as simply morning sickness)? My mother had it and was left to cope what is bad enough to get a disgnosis? How do I get diagnosed rather than told it's normal?
27	10	Is having HG a risk factor for requiring induction/c-section, miscarriage, other conditions/complications of pregnancy, or particular birth outcomes?	No	49	Multiple case-control and cohort studies identified. An SR may be able to answer this.	30	29	2	1	4	1	1	Whats the connection with HG en pregnancy diabetes? Could HG be related to any other sickness or disorder in my future, just as gestational diabetes is related with the chances of developing diabetes type 2 after pregancies? Are thyroid issues related to first having HG? Induction and C section rate amongst HG? Is there a link between hg and gestational diabetes? Is HG linked to 1gall bladder dysfunction? 2gestational diabetes 3 sluggish digestion 4 thyroid levels? Has there been any link between HG sufferers and delivery type? I.e. an increased chance of forceps delivery?
15	11	Does HG run in families? What is the level of risk of experiencing HG for a person with a family history of HG?	No	7	Multiple studies identified. An SR may be able to answer this.	74	63	6	8	10	0	2	Is HG heritable? If my mum had HG what are my chances of getting it? How likely is it to get HG if both mother and grandmother had it? Can anything specific mitigate the possibility of children experiencing hyperemesis themselves during future pregnancies, if their mother suffered hyperemesis? As I've had HG in my pregnancy what is the likelihood of a close relative suffering from the condition as well? E.g. sister or daughter. Because I had hyperemisis what are the chances of my daughter having this?
26	12	How do factors such as diet, nutrition, stress, tiredness and rest affect symptoms and coping? What can exacerbate symptoms and how can we manage those factors?	No	3	3 studies identified, each on different topics.	31	26	4	3	2	1	2	What is the effect of rest/tiredness on vomiting during pregnancy? Are there any triggers which can worsen hyperemesis? Are there and factors that aggravate HG? What factors (biological ans psychological) exacerbate HG? And how can these be managed during HG? Some foods increase phlegm/mucus and some foods increase throwing up. Is there a relation? Does fatigue/tiredness/ workload affect the nausea and vomiting exerienced? Do certain foodsubstances have more influence on HG?

5	13	Is HG preventable? What is the effect of preventative treatment or early intervention on the severity and duration of HG in a subsequent pregnancy?	No	1	One study which does not sufficiently answer the question	168	152	22	5	12	1	1	Is there anything the patient could have done/can do different to prevent it? What can be done to help prevent HG in those identified as at risk, I e those who have had it in previous pregnancy. What factors determine duration - do certain treatments or early intervention affect duration or is it determined by the underlying cause? What is the best way to get HG under control ASAP and limit the mental effects to the patient? If you start taking medication early on in pregnancy will this lessen symptom severity overall?
20	14	What are the disease characteristics of HG? (Prevalence, onset, duration, symptoms, peaks?)	No (and yes)	14	Some aspects answered such as prevalence, but other aspects such as onset, duration, symptoms and peaks not answered. Prevalence SR and meta analysis concluded: The prevalence of HG was 1.1% (CI95%:0.8%-1.3%), with a range of 0.3%-3.6%. Almost 70% of women worldwide experience NVP, but reported rates vary widely. HG, the most severe form, affects 1.1%. Ref: Einarson TR, Piwko C, Koren G. Quantifying the global rates of nausea and vomiting of pregnancy: a meta analysis. Journal of population therapeutics and clinical pharmacology = Journal de la therapeutique des populations et de la pharamcologie clinique. 2013;20(2):e171-83.	40	35	8	2	1	1	0	Numbers of how often HG occurs, are there any differences in different countries / parts of the world? Are there differences in the rate of HG across ethnic groups? How long is this likely to last? What is the chance of HG easing and then returning ? While suffering from H G I tend to be spitting every few seconds, can’t swallow any of my saliva (spit cup necessary) any research on how long this spitting last’s for? What kind of statistics are there regarding Hg? (Recurrence, who gets it, how long it will last...) Are there certain weeks when hormones always surge to make HG worse? What are the chances that this feeling will last the entire pregnancy? At what week does HG usually improve a bit? How common is it to have HG the whole pregnancy? What is the incidence of HG continueing after the first trimester and for how long does it continue?
23	15	How can symptoms of HG, other than vomiting, be effectively treated? For example, the nausea, excessive saliva, extreme sense of smell and fatigue.	No	5	Some prevalance studies on ptyalism (excessive salivation) identified. No discussion of other symptoms in guidelines	35	34	2	2	3	0	0	Is there an effective treatment for ptyalism?Is there anything to reduce the excess saliva that comes hand in hand with HG?One of the most distressing symptoms of HG for many women is the heightened sense of smell. Is there a medical way to target that symptom in particular?The sickness and nausea is obviously a big part of HG. But how do we treat the less talked about effects including extreme tiredness, no energy, headaches, dizziness, breathlessness, confusion, being unable to stand/endure movement or motion/listen to conversations/music without feeling like you are going to be sick or faint? How can the other symptoms be reduced (i.e. smell sensitivity, excessive saliva, extreme fatigue, recurring UTIs). Is there a way to control the extreme sensitivity to smell, which causes so much agony? Is there anything that can be done to dull the extremely heightened sense of smell that a lot of HG patients suffer with? As I honestly think it would have helped with the severity of the constant nausea in my case.

2	16	What are relative efficacies of the current medications and treatment options available? What are the optimal dose, route, timing and combination of the medications and what are the related side effects?	No	58	Cochrane review concludes: there is little high-quality and consistent evidence supporting any one intervention. Ref: Boelig RC, Barton SJ, Saccone G, Kelly AJ, Edwards SJ, Berghella V. Interventions for treating hyperemesis gravidarum. The Cochrane database of systematic reviews. 2016;5:DOI: 10.1002/14651858.CD010607.pub2. HTA SR found: there is little on the effectiveness of treatments in more severe NVP/HG. Ref: O'Donnell A, McParlin C, Robson SC, Beyer F, Moloney E, Bryant A, et al. Treatments for hyperemesis gravidarum and nausea and vomiting in pregnancy: a systematic review and economic assessment. Health Technol Assess. 2016;20(74):1-268	253	195	71	11	19	5	8	What are the options for treating it? What is the best dosage of x (insert any of treatments) for most effective results for most women? What are all the options available to women who experience HG? My doctor was very dismissive. What are the recommended first, second and third line anti-emetic drugs in HG? When to switch over to the first line to second line, and so on? Which is the highest dose of Cariban? Best regime. Why do doctors not readily prescribe combinations of anti-emetics when patient's symptoms appear unimproved with one anti-emetic. For example cyclizine and metoclopramide?
24	17	How can we most effectively manage HG? What clinical support measure is most important to women who have had hyperemesis and what did they find most beneficial? eg medical management, pharmaceutical review, nutrition support, rehydration, psychological support	No	13	Some trials, case-control and case studies for various support measures. No SR, no large well conducted trials.	34	27	6	3	5	0	1	What's the best way to manage HG? What clinical support measure is most important to women who have had hyperemesis and what did they find most beneficial? eg medical management, pharmaceutical review, nutrition support, rehydration, psychological support. What are the best treatment methods for Hyperemisis Gravidarum? What is the treatment recommendations? (Including medicines, food intake, intravenous therapy, psychological help...) How can we use the medical intervention available to us to make this disease more bearable for mothers who suffer?
17	18	Can we find a cure? What novel or new treatments are being developed/tested/used elsewhere which could have a curative effect and to address all the symptoms of HG rather than just the vomiting?	No	9	Some small and novel trials identified for gabapentin, clonidine, mitrazepam, marijuana and other therapies.	66	52	7	3	6	2	7	Is there a cure? Will there ever be a cure for this 'HG'? is there not a surgical way or medical way to eradicate this reaction? By what treatment can HG be totally cured? This means de vomitting, the nausea, lack of feeling hungry, etc. Given the 2 genes (contributing to appetite and placenta) which were recently found to be connected with the root causes of Hyperemesis Gravidarum, how can one reduce his / her chances of acquiring this sickness or cure it in an earlier stage than 20 weeks plus or the entire pregnancy? Please try to find a cure!!

4	19	What is the effect of HG on mental health during pregnancy? What is the efficacy of psychotherapy on symptom management/pregnancy outcomes/quality of life? How can women access psychosupportive services during pregnancy?	Yes and No	28	Systematic review and meta-analysis have shown a significantly increased frequency of depression and anxiety in women with HG. Ref: Mitchell-Jones N, Gallos I, Farren J, Tobias A, Bottomley C, Bourne T. Psychological morbidity associated with hyperemesis gravidarum: a systematic review and meta-analysis. Bjog. 2017;124(1):20-30. For interventions, various studies have assessed interventions, SR may be possible but question is not currently answered.	188	172	20	7	25	2	6	How does the mental component of HG (desperation, exhaustion, feeling that 'I can't do this for another eight months') influence the patients physical wellbeing and how do we include mental health care in the treatment? Can mental health support be provided at home for those who are unable to travel alone? How do we support women with HG before, during and after pregancy psychologicly? What mental health support is available for women and how can we improve access to this service for women who struggle to leave the house when suffering with HG? What support is available for the psychological repercussions of HG? Comparison between conventional medical treatment and psychotherapy for hyperemesis gravidarum. What are the barriers to women accessing counselling for HG? Suicidal thoughts and HG. How can this be better supported by the the community midwife and mental health team?
33	20	How can the condition be effectively managed in the community to prevent lengthy hospital admissions?	No	7	Some studies comparing ambulatory to inpatient care.	17	13	6	0	1	0	1	What is the best management or treatment to get women with HG out of the hospital and back home rather than staying in hospital? What can be done to keep HG sufferers home instead of in hospital? Can hyperemesis patients prevent themselves getting into a situation where they need to be admitted to hospital by having regular IV drips, perhaps day appointments for this or community midwifes administer this as a method of prevention/management of the condition? Are women who are car d for at home with HG more or less likely to suffer from post natal mental health issues?
25	21	Do clinical treatment guidelines for HG improve management and outcomes? And if so how can guidelines be developed and implemented nationally (where none exist) and internationally for hospital and community settings? What should be included in guidelines?	No	6	Some audits of local guidelines highlight inconsistent treatment. Effectiveness of national RCOG and ACOG guidelines have not been evaluated.	33	28	6	0	1	0	3	Would there be a benefit to developing a national best practice guidance for managing HG? can there be one HG protocol for every healthcare provider? Make a list of available medication along with safe dosage available to all medical professionals, as all too often treatment/medication given varies between hospitals, due to lack of information. Is it possible to create a worldwide guide so docters treat everybody the same. There is so much difference between country's but even between hospitals and docters.
13	22	What are the barriers to accessing treatments/services and how can we reduce them to improve access?	No	4	Four small studies/case studies identifying barriers to accessing treatment.	95	86	11	5	7	2	1	What treatment is available, how effective is each treatment and how do I access it? When partner is to ill to communicate how do I get her the best medication or know where to go for help? How can I initiate the process for home care with my insurance if homebound with HG? How can I access IV hydration at home? How can we support medical/nursing teams to access specialist mental

													health support for HG sufferers/families when NHS budgets are constantly being cut?
30	23	What self-management and coping strategies and treatments do people with HG find most helpful?	No	0	None identified	20	17	2	2	0	0	0	What can make women with HG feel better? I tried many thing nutricion , medication , many sleep but nothing worked out, what worked out for the best for other woman ?
34	24	What percentage of people with HG are satisfied with the care and treatment they received? How can satisfaction be increased and how can healthcare professionals best support people with HG?	No	0	None identified	17	13	3	0	2	0	1	How can we help in clinic? What percentage of women are satisfied with the treatment they receive for HG? How can quality of care be improved for women with HG? Were the midwives and doctors helpful whilst you were suffering? Which support and treatment options do women attribute to helping them have a positive pregnancy experience (eg a care plan, continuous care from a midwife, early diagnosis and treatment, help to make informed choice regarding treatment options etc)?
37	25	How can dehydration be prevented/managed without an IV drip?	No	0	None identified	13	10	2	0	1	0	1	What am I supposed to do when I can't keep liquid down to stay hydrated? Can the priority shift from treating dehydration to preventing dehydration? How dangerous is getting towards dehydration for longer periods or more often for the vascular system of the uterus and effects on the foetus?
10	26	What healthcare services exist and how can they collaborate and be organised to better identify, treat and support women with HG? For example, do services such as outpatient clinics or IV at home, improve outcomes and reduce the physical/mental burden of the condition?	No	18	Some audits, case-control studies and RCTs identify services such as Home Healthcare and outpatient services in specific areas of individual countries. SR of outpatient verse inpatient outcomes may be possible.	97	78	21	4	12	3	2	How could the healthcare system be better equipped to identify and treat hg? which reproducible model of management (inpatient day unit/ via a&e) has the best outcomes? How can consultant-led hyperemesis specialty clinics in maternity services improve co-ordination of care and outcomes for women? Do you think there is better scope for community treatment of Hyperemisis Gravidarum to improve women's lifestyles and mental health? Do women prefer inpatient or outpatient care for hyperemesis? What is the cost/impact of HG management in acute settings vs a community based model?
14	27	What other services are available/could be developed to support families affected by HG? And how can access be enabled to services which currently exist	No	2	Two studies suggest counselling and martial support may be helpful	78	70	12	6	5	0	0	Can mental health support be provided at home for those who are unable to travel alone? Is there any association of volunteers to help or support the expectant mother, husband and or older siblings in everyday life? E.g. picking up medication for mom, prepare food, taking care of kids...
19	28	How long does it take people to recover from HG? What post-pregnancy is care available (or could be developed) for people who have suffered HG during pregnancy and what is its impact on recovery?	No	2	Two studies (one survey, one case-control) looking at aftereffects and recovery identified.	50	46	1	4	9	1	1	How long does the average HG sufferer take to 'get over' the trauma of her pregnancy? Do some never get over it? What aftercare can be made readily available for women who have had hg during pregnancy? How does postpartum care need to be organised to shorten recovery?

47	29	Do specific specialist healthcare professional roles for conditions such as HG improve outcomes? How can such roles be developed for midwives/nurses/doctors?	No	0	None identified	7	6	2	0	1	0	0	Will there be special hg physicians in the future? Can we look into a community midwife specialising in hyperemsis? It would be nice if there is a special HG clinic or team that is specialized in HG. Would that be possible in the near future? Which doctor (gynecologist)is the best, most experienced doctor for treating Hyperemesis gravidarum in Europe?
35	30	How does HG treatment/management vary by country (and regions within countries) and how do the variations effect outcomes?	No	0	Various types of services reported in studies and guidelines from around the world. No research addressing question.	16	14	1	0	1	0	1	Why is the medical treatment different in an other country? Here in the Netherlands you can only get emesafene and sometimes metoclopramide for max 5 days. Why is the treatment in the uk not as efficient as that of the us? (I.e home care, pick lines). Why is care so different in so many areas ? Why do the treatments vary so much per hospital/country/doctor
44	31	What additional checks, tests and medical reviews should people with HG be offered during pregnancy?	No	17	Various tests proposed/highlighted in case reports and case-control studies. SR may be helpful to address.	8	8	1	0	3	0	0	Should you be seen more by consultant when pregnant with HG? How often should a doctor review the patient's condition and treatments? Why don't women with HG get more regular or routine appointments to check the healthy development of the foetus? Why are HG sufferers not offered earlier blood tests to monitor iron levels etc? when do you consider further investigations like imaging/endoscopy in cases of persistent hyperemesis beyond 16 weeks of pregnancy? why aren't women who suffer from HG monitored more closely to make sure baby's growth is going well? When you vomit many times a day for months, why aren't electrolyte levels checked?
8	32	Do negative stigmas, attitudes and beliefs surrounding HG affect the quality/consistency of care provided on individual healthcare professional and institutional levels? If so, what is the impact of such stigmas and how can they be addressed?	No	1	A number of studies identified that found persistent stigmatisation of HG among healthcare professionals in the UK. Qualitative SR may be possible	112	108	9	4	10	3	0	Why does the quality/consitency of care, in HG patients, differ so much between medical professionals? Why is the care so variable - I.e from one gp to the next? Why is it so difficult to access rapid and effective treatment? Having to fight for anti emetics and rehydration and being treated like a hysterical addict by doctors adds to the trauma of hg. Why is it so difficult to access the right medication and treatment in some areas? How can you get taken seriously by you gp when you report this, and not just brushed off as being silly? How can I explain what I'm feeling to my doctor in a way that will help me be taken seriously? What are the consequences for the patient when professionals say to them that it's a psychological issue, even if they still treat the patient physically?

51	33	How many people terminate their pregnancy due to HG symptoms, lack of access to treatment or other related factors? What are their experiences and how can they be supported?	No	1	Some studies identifying rate of termination but no conclusive evidence. SR may be possible.	6	6	1	1	0	1	0	How often do women terminate wanted pregnancies because the hg is too difficult? How many terminations are carried out because of lack of access to treatment for HG
53	34	When is it appropriate to induce labour early and/or conduct elective c-section due to third trimester HG?	No	0	No papers identified and no mention of induction/elective section in guidelines	4	3	0	0	0	0	1	Can I be induced at 36 weeks because of the severity of hg? If an unborn child can be delivered- healthy as early as 36 (?) weeks, why are doctors not relieving HG mothers of their symptoms who are still suffering near the end of their third trimester. If the woman with Hyperemesis Gravidarum suffers from the condition throughout the whole pregnancy and is severely anemic and weak, is elective Cesarean section recommended, that is, is that woman in greater risk of having complications enduring vaginal birth?
28	35	How can people with a history of, or significant risk factors for HG be supported to plan for a pregnancy and does such planning improve outcomes? What should a pre-pregnancy plan contain?	No	0	RCOG guidelines suggest possible benefit to prophylactic treatment but evidence is poor. No evidence for pre-pregnancy care plan.	27	24	2	2	5	0	1	Planning for a second pregnancy can be very difficult. How can barriers be broken down for more women before pregnancy ? How can we better support women who are terrified to have another child?
31	36	What is the role of vitamin supplementation during HG, which vitamins are important, how should they be administered, and do they effect outcomes?	No	36	Multiple articles addressing various vitamins particularly Vit K and B1. SR may be possible.	20	17	3	2	3	0	1	It is safe to take vitamins if I have HG? I was unable to swallow pregnancy tablets and was malnourished through being unable to eat or drink for 6 months. I received one dose of vitamins through a drip in this time. Why was I not given these regularly.
60	37	How can appointments and clinics within all possible settings (home, GP, hospital etc) be made more ‘HG friendly’? (ie. Reducing sensory stimulations, smells, noise, location, access to toilet/sick bowls etc)	No	0	No studies identified	2	1	1	0	0	0	0	How can appointments and clinics within all possible settings (home, GP, hospital etc) be made more accessible and ‘HG friendly’?
38	38	What are the barriers to taking/prescribing medication for HG? How can the risk and benefits of HG and its treatments be better communicated to support informed decision making and consent to treatment?	No	1	Some barriers identified but no conclusive studies within HG literature. A wider search regarding medication use in pregnancy may yield more insight which is applicable to HG?	13	11	2	0	2	0	1	The biggest worry is taking medication and any impact that may have on your baby, clear advice and information needs to be provided prior to taking any medication. I was only offered stemitil which didn't help and only learned afterwards other medication was available.
54	39	What are the barriers to medication testing and licensing for use during pregnancy and how do we try to overcome those barriers?	No	0	No results within HG literature but a wider search regarding general medication testing in pregnancy may yield more insight which is applicable to HG?	4	4	0	0	0	0	0	Need of RCTs whene it comes to effect of medications? Why are there so few medications licensed and well-tested for use for HG during pregnancy? Has odansetron become licensed for use in pregnancy yet? Can it become standard for GP’s to prescribe nationwide?

56	40	Do personalised care plans reduce suffering and improve outcomes for people?	No	3	One RCT assessed difference between patients receiving individual holistic care package and controls receiving "normal care". No statistical difference was found. Further studies required.	3	2	1	0	1	0	0	How can action plans be written up to ensure dehydration, hospitalisation are reduced? Such as day visits for fluids, and consult led care
6	41	What is the currently level of knowledge about HG and its treatments among healthcare professionals (particularly GPs)? How can effective education for healthcare professionals be designed and delivered to improve the general knowledge and awareness of HG among HCPs?	No	5	Some studies/audits have tried to assess local current knowledge but do not address how education can be improved or delivered.	148	138	9	8	22	2	4	I think raising awareness in medical professionals is the best way to support women with HG. How can we do this? Could all hospitals and medical professionals be better educated as to how they deal with HG? Is there a process to follow? considering complications, general state, psychological state, evolution of the pregnancy, effects on patient's day to day life and for patient's surroundings: are there significant differences between patients treated by well informed professionals and patients treated by professionals who don't have particular knowledge of HG ?
32	42	What is the most effective IV rehydration regime: which solution in what quantity over what time period and how frequently? Does regular rehydration improve symptoms/outcomes/quality of life?	No	5	Lack of clear studies, current recommendations in guidelines from low level evidence.	19	12	7	1	0	0	0	Is there a difference between different electrolyte infusions? What is the best IV fluid regimen in women with HG? How effective is rapid rehydration as an outpatient treatment?
36	43	How can weight loss/malnutrition from HG be effectively managed/reduced? Do nutritional therapies such as NG tubes, TPN, PEG feeding improve outcomes?	No	15	Some studies and case reports of enteral feeding for HG with mixed results. Lack of evidence for nutritional therapies in general. ACOG guidelines vague on enteral feeding, discourages parenteral feeding with low level evidence references but lacks alternatives to managing weight loss and malnutrition	14	10	4	0	0	0	0	Whicht treatment to reduce the HG and minimize the weightloss? Is it nutrition or IV for example? What role does IV nutrition play in preventing long term health impacts for HG babies? What role does IV nutrition play in preventing premature birth?
57	44	What are the risks and benefits to using Peripherally Inserted Central Catheters (PICC) lines and central lines for people with HG requiring regular IV therapy?	No	2	ACOG and RCOG references a number of low-level evidence sources highlighting risks associated with PICC lines in pregnancy.	3	3	0	0	0	0	0	If I am going to emerge 3 or 4 times a week for IV fluids and they have trouble finding veins because of dehydration why can't they put in a port or picc earlier why must we suffer so long and get yelled at for a shitty veins ?
61	45	How should treatment vary throughout pregnancy according to physiological changes and metabolic requirements? Ie. Should doses increase later in pregnancy, are some medications less suitable close to delivery	No	0	No studies identified	2	1	0	1	0	0	0	Does choice of best medication to treat HG change with gestation?

55	46	How does HG impact on a person's (and their family's) quality of life? How does quality and efficacy of treatment impact that effect?	Yes and No	7	Negative effects identified in a qualitative systematic review ref: Dean C, Bannigan K, Marsden J. Reviewing the effect of hyperemesis gravidarum on women's lives and mental health. British Journal of Midwifery. 2018;26(2): Plus further studies since then add to evidence base. However, no evidence identified on how quality and efficacy of treatments mitigate or exacerbate the effect.	4	3	2	0	0	2	0	What is the range of difference in quality of life for patients as linked to the medicines they are given?
58	47	Why are some cases of HG unresponsive to all antiemetics and how can we treat such cases?	No	9	Cause of unresponsiveness not addressed in literature. Various novel therapies, enteral/parenteral feeding and termination proposed for unresponsive cases in a small number of papers and the two guidelines.	3	3	0	0	0	0	0	Why in some cases is it not possible to control the sickness and vomiting? Why doesn't medication work (good enough)? Why do different medications or combinations of medications work (or not work) for different patients? Why don't pills or medications work against the hg effects?
12	48	What are the immediate and long-term effects of the various medications/treatments on the developing foetus throughout the various stages of pregnancy and in varying doses or combinations of treatments?	No	20	Various studies and reviews of individual medications for particular exposure periods. For example, SR found: "first trimester ondansetron exposure is not associated with an overall statistically significant increased risk of congenital malformations or cardiovascular defects." ref: Balayla, J.; D'Alton, M. E. 2016 First trimester ondansetron exposure and the risk of major congenital malformations and heart defects: A systematic review and meta-analysis. American Journal of Obstetrics and Gynecology - Volume 1, Issue 0, pp. S177. This doesn't answer the question on whether ondansetron is safe throughout pregnancy, third trimester or in what doses or in combination with other medication	96	80	19	2	9	0	3	Do any of the medicines harm the baby? Does the type, dose and duration of medication have different effects on the baby depending on the time of the pregnancy the medication is used. For example, does ondansetron have a different (potential) effect on the baby if this is taken in the first trimester vs the last trimester? Is this drugs cocktail really the best thing for baby?
45	49	What dietic service support is currently available to people with HG within maternity services? What impact does dietic care have on clinical outcomes and quality of life?	No	0	No studies identified, Guidelines suggest dietician involvement based on expert opinion	8	4	5	0	0	0	0	What is women's experience of dietetic support in maternity services? What is women's experience of dietetic support in maternity services? What impact does dietetic care have on clinical outcomes and QoL for women with hyperemesis? Is there any improvement in clinical outcomes in those who recieve dietetic input versus those who do not?

21	50	What are the nutritional requirements of the 1st, 2nd and 3rd trimesters and how can people with HG achieve these goals? Ie. Oral supplements, fortifying food, dietary measures	No	1	None identified within HG specific literature but a search within wider pregnancy literature may identify evidence relevant to this question.	38	32	5	4	6	0	0	Is there benefit in providing hyperemesis patients with oral nutritional supplements? Which foods are best to eat when suffering from HG? What is the best way to gain and retain vital nutrients during HG? What dietary advice is best for a HG-patient?
63	51	Does physiotherapy have a role to play in HG management?	No	0	No studies identified. Guidelines suggest multidisciplinary approach	1	1	0	0	0	0	0	Would having some Physiotherapy possibly reduce the risk of blood clots and help mental health Of those suffering HG?
22	52	What are the wider biopsychosocial impacts of HG on partners, families, older children, relationships, work and finances? And how can families be supported?	No	5	some studies address wider family impacts. SR maybe possible. No studies on support for families	37	34	1	0	4	2	2	What is the impact of HG on the woman's partner/ family (e.g. having to take time off work, becoming a carer, feeling hopeless and helpless) and how does HG change relationships
7	53	What are the immediate and long term effects of HG (including malnutrition and dehydration, stress) on the developing foetus?	Yes and No	56	Some immediate effects identified in systematic review but long-term consequences require further study: Veenendaal MV, van Abeelen AF, Painter RC, van der Post JA, Roseboom TJ. Consequences of hyperemesis gravidarum for offspring: a systematic review and meta-analysis. Bjog. 2011;118(11):1302-13.	130	112	25	3	8	1	3	What are the effects/consequences of HG for the baby and the mother in long term? What are the risks to the fetus when a woman has Hyperemisis Gravidarum? What are the long term effects of a HG pregnancy on the health of a child? (Impact of medication but also, is there any impact on the child anyway without meds e.g. long term growth, IQ, physical health, mental health etc)
9	54	What are the immediate and long term, physical, mental and social consequences and complications of HG (including malnutrition and dehydration) on the pregnant person's body? (ie. Metabolic impact, DVT, depression, effects of dehydration)	No	136	Psychological morbidity has been demonstrated with systematic review: Mitchell-Jones, N., et al. (2017). "Psychological morbidity associated with hyperemesis gravidarum: a systematic review and meta-analysis." Bjog-an International Journal of Obstetrics and Gynaecology 124(1): 20-30. A myriad of other consequences and complications have been identified in the literature but no systematic review.	110	95	12	5	12	2	6	What are the most common complications of HG? How can we raise the risk of embolism, so women know when to seek help? What are the longterm effects of HG for mother and baby? Metabolic impact of hyperemesis during and after pregnancy? What are the long tern effects of HG (i.e. up to 5 years after the pregnancy)? Is HG linked to higher rates of postnatal depression, family breakdown, and decisions not to have any other children? How many HG sufferers had to stop working prematurely?
41	55	Does HG effect breastfeeding or early bonding and attachment with the baby?	No	3	Some studies looking at attachment and bonding with conflicting results. Not enough for SR. Original research required.	11	10	1	1	1	0	0	What is the impact of HG on early bonding and attachment with baby? How does HG affect breastfeeding? How does the experience of HG affect subsequent bonding and attachment between the mother and baby? Can you breastfeed through HG (i.e. breastfeed an older child -- I did but couldn't get any support or find anyone else who had done it)? Are women with HG less likely to breastfeed?

42	56	Does HG impact on people's reproductive choices and/or limit families? Does this have an effect on family wellbeing and mental health?	No	2	Mixed results from a couple of heterogeneous studies SR not possible yet, research needed	10	9	3	0	0	0	0	How does HG affect the likelyhood of a family choosing to have another baby? Do you feel as though your experience with HG was limiting/ debilitating enough to make you not want to have another child, as opposed to if you hadn't suffered HG during your pregnancy? was HG a reason not to get any more children? Did HG stop you having further pregnancies?
48	57	How should people with comorbidities be managed while suffering HG, for example, diabetes, epilepsy, mental health conditions?	No	21	Various case reports of a variety of comorbidities with HG and a cohort study looking at diabetes. Further research needed. An SR on the case reports of gastric bands/bypass in relation to HG may be possible.	7	5	3	0	0	0	0	How do I maintain adequate blood sugar control as a patient with type 1 diabetes and hg? How can we best handle gestational diabetes when HG impacts what and when and if you can eat? If a type 1 diabetic woman gets HG what should she do? How does Syndrome Gilbert affect pregnant woman, especially one that suffers from Hyperemesis Gravidarum? How does HG effect women with current or past eating disorders? As someone that already suffers with mental health problems is there a chance hg has made things worse ?
16	58	How can awareness of the condition be raised among the public/employers/media etc? How does the public perception of the condition affect people suffering HG?	No	0	No studies identified	67	59	3	8	7	0	4	What do employers need to know in order to be able to support women with HG? The general public should be made aware of this condition - any plans to publicise this illness? Many people do not understand the severity of HG. How can it be explained to family members and employers? How can we all make the world more aware of what HG is and the horrific issues behind it?
49	59	What is the total economic cost to the individual, their family, the health system, and society when a person has HG? How do differing models of care alter this?	No	8	An SR which attempted economic analysis of treatments for HG/NVP found" The economic analysis was limited by lack of effectiveness data", Further research required. Ref: O'Donnell A, McParlin C, Robson SC, Beyer F, Moloney E, Bryant A, et al. Treatments for hyperemesis gravidarum and nausea and vomiting in pregnancy: a systematic review and economic assessment. Health Technol Assess. 2016;20(74):1-268	7	4	4	0	0	1	0	What is the total economic cost to the individual, her family, the health system and society when a patient has hyperemesis gravidarum? Explore economic burden of severe NVP/hyperemesis in different countries (direct + indirect cost). What is the cost/impact of HG management in acute settings vs a community based model. What is the estimated annual cost to the national economy of working-days lost due to HG and related conditions?
50	60	How can HG research be improved and stimulated? Which treatment outcomes are meaningful to patients, what definition of HG can be used for research, what light can qualitative research shed on woman's experiences of HG, can a database of HG offspring be established, How can RCTs	No	5	Problems with definition and outcomes identified in Systematic Review which demonstrates need for consensus on these for research: Koot, M. H., et al. (2018). "Variation in hyperemesis gravidarum definition and outcome reporting in randomised clinical trials: a systematic review." Bjog-an International Journal of Obstetrics and Gynaecology 125(12): 1514-1521.	7	2	5	0	0	0	0	Which treatment outcomes are meaningful to HG patients? Can we agree on an international definition of HG? women’s experiences of severe HG qualitative research? Are the children born to HG sufferers put on any sort of database to try and collate some sort of information for future medical survey/information reference? I have a more general question, methodological. Which (Medical) interventions improve the relevant outcomes. I want to see rcts! No more or much less observAtional

		of medical treatments be encouraged and supported?											
59	61	What other diagnosis should be considered, and at which points in pregnancy/disease stage?	No	70	Many case reports of differential diagnoses at varying points in pregnancy, SR would be helpful. Guidelines list various possible differential diagnosis based on low quality evidence and expert opinion.	3	2	1	0	0	0	0	when do you consider further investigations like imaging/endoscopy in cases of persistent hyperemesis beyond 16 weeks of pregnancy
62	62	Would an alternative name/classification for hyperemesis gravidarum better reflect the scope of the condition and improve awareness/reduce stigma?	No	0	No evidence identified. It may be worth looking at literature from other condition which have been renamed to assess potential impact of such work.	2	1	1	0	0	0	1	What can we rename HG which reflects that some women don't have severe vomiting but severe nausea which leads to same outcomes
64	63	What are people's rights to treatment and how can people with HG be enabled to exercise their rights?	No	0	No literature identified.	1	1	0	0	0	0	0	What are my rights if GP is refusing to offer medicines?
52	64	What is the mode of action for the various medications?	No	0	No specific reports within the HG literature identified. Searches on specific medications within pharmacology literature may yield better results.	5	4	1	0	0	0	0	What is the action of the various medications prescribed? I've seen caraban used for HG how does this mechanism work? Why is HG better controlled on a combination of medicines rather than just one?
43	65	What employment rights do people with HG have and what financial support is available to them?	No	0	No literature identified in relation to HG, general pregnancy literature searches per country may yield more results	9	8	0	1	1	0	0	
65	66	Are there survival/evolutionary benefits to HG?	No	3	Some studies proposing theories, no SR identified.	1	0	1	0	0	0	0	Are there survival/evolutionary benefits to HG?
