# Supplementary material for: A patient–clinician James Lind Alliance partnership to identify research priorities for hyperemesis gravidarum
Source: BMJ Open. 2021 Jan 15;11(1):e041254. doi: 10.1136/bmjopen-2020-041254 (PMC7813320; doi:10.1136/bmjopen-2020-041254)
Supplement: Author's manuscript [file bmjopen-2020-041254.draft_revisions.pdf]

BMJ Open is committed to open peer review. As part of this commitment we make the peer review history of every article we publish publicly available.

When an article is published we post the peer reviewers' comments and the authors' responses online. We also post the versions of the paper that were used during peer review. These are the versions that the peer review comments apply to.

The versions of the paper that follow are the versions that were submitted during the peer review process. They are not the versions of record or the final published versions. They should not be cited or distributed as the published version of this manuscript.

BMJ Open is an open access journal and the full, final, typeset and author-corrected version of record of the manuscript is available on our site with no access controls, subscription charges or pay-per-view fees (http://bmjopen.bmj.com).

If you have any questions on BMJ Open's open peer review process please email info.bmjopen@bmj.com

BMJ Open

A Patient-Clinician James Lind Alliance Partnership to Identify Research Priorities for Hyperemesis Gravidarum

Journal:	*BMJ Open*
Manuscript ID	bmjopen-2020-041254
Article Type:	Original research
Date Submitted by the Author:	04-Jun-2020
Complete List of Authors:	Dean, Caitlin; Amsterdam UMC Location AMC, Obstetrics and Gynecology; Pregnancy Sickness Support<br>Bierma, Hyke; Amsterdam UMC Location AMC, Obstetrics and Gynecology<br>Clarke, Ria; Frimley Park Hospital NHS Foundation Trust, Obstetrics and Gynaecology<br>Cleary, Brian; Royal College of Surgeons in Ireland, School of Pharmacy; Hyperemesis Ireland<br>Ellis, Patricia; James Lind Alliance<br>Gadsby, Roger; Warwick Medical School, Institute of Clinical Education; Pregnancy Sickness Support<br>Gauw, Norah; ZEHG Foundation<br>Lodge, Karen; Pregnancy Sickness Support<br>MacGibbon, Kimber; Hyperemesis Education Research<br>McBride, Marian; Hyperemesis Ireland; Health Service Executive, Strategic Planning and Transformation<br>Munro, Deirdre; Portiuncula Hospital; Hyperemesis Ireland<br>Nelson-Piercy, Catherine; Guy's & St Thomas' Foundation Trust<br>O'Hara, Margaret; Pregnancy Sickness Support<br>Penny, Helen; Cardiff University, School of Psychology<br>Shorter, Katherine; Queen's Medical Centre Nottingham University Hospital NHS Trust<br>Spijker, René; Amsterdam UMC Location AMC<br>Trovik, Jone; Haukeland University Hospital, Obstetrics and Gynaecology<br>Watford, Emma; Pregnancy Sickness Support<br>Painter, Rebecca; Amsterdam UMC Location AMC, Obstetrics and Gynaecology
Keywords:	Maternal medicine < OBSTETRICS, OBSTETRICS, HEALTH SERVICES ADMINISTRATION & MANAGEMENT, STATISTICS & RESEARCH METHODS

SCHOLARONE™
Manuscripts

I, the Submitting Author has the right to grant and does grant on behalf of all authors of the Work (as defined in the below author licence), an exclusive licence and/or a non-exclusive licence for contributions from authors who are: i) UK Crown employees; ii) where BMJ has agreed a CC-BY licence shall apply, and/or iii) in accordance with the terms applicable for US Federal Government officers or employees acting as part of their official duties; on a worldwide, perpetual, irrevocable, royalty-free basis to BMJ Publishing Group Ltd ("BMJ") its licensees and where the relevant Journal is co-owned by BMJ to the co-owners of the Journal, to publish the Work in this journal and any other BMJ products and to exploit all rights, as set out in our licence.

The Submitting Author accepts and understands that any supply made under these terms is made by BMJ to the Submitting Author unless you are acting as an employee on behalf of your employer or a postgraduate student of an affiliated institution which is paying any applicable article publishing charge ("APC") for Open Access articles. Where the Submitting Author wishes to make the Work available on an Open Access basis (and intends to pay the relevant APC), the terms of reuse of such Open Access shall be governed by a Creative Commons licence – details of these licences and which Creative Commons licence will apply to this Work are set out in our licence referred to above.

Other than as permitted in any relevant BMJ Author's Self Archiving Policies, I confirm this Work has not been accepted for publication elsewhere, is not being considered for publication elsewhere and does not duplicate material already published. I confirm all authors consent to publication of this Work and authorise the granting of this licence.

For peer review only

A Patient-Clinician James Lind Alliance Partnership to Identify Research Priorities for Hyperemesis Gravidarum

Caitlin Dean[1,2], Hyke Bierma[2], Ria Clarke[3], Brian Cleary[4,5], Patricia Ellis[6], Roger Gadsby[1,7], Norah Gauw[8], Karen Lodge[1], Kimber MacGibbon[9], Marian McBride[4,10], Deirdre Munro[4,11], Catherine Nelson-Piercy[12], Margaret O'Hara[1], Helen Penny[13], Katherine Shorter[14], René Spijker[2], Jone Trovik[15], Emma Watford[1], Rebecca C. Painter[2]

1. *Pregnancy Sickness Support, 19G Normandy Way, Bodmin, Cornwall, UK*
2. *Amsterdam University Medical Centres, University of Amsterdam, Meibergdreef 9, 1105 AZ Amsterdam, the Netherlands*
3. *Obstetrics and Gynaecology, Frimley Park Hospital, Surrey, UK*
4. *Hyperemesis Ireland, Dublin, Ireland*
5. *School of Pharmacy, Royal College of Surgeons in Ireland, Dublin, Ireland*
6. *James Lind Alliance, Southampton, UK*
7. *Warwick Medical School, University of Warwick, UK*
8. *ZEHG Foundation, The Netherlands*
9. *Hyperemesis Education and Research Foundation, Damascus, Oregon USA*
10. *Strategic Planning and Transformation, Health Service Executive, Dublin, Ireland*
11. *Portiuncula University Hospital Galway, Ireland*
12. *Guy's & St Thomas' Hospital, London, UK*
13. *School of Psychology, Cardiff University, Cardiff, UK*
14. *QMC, Nottingham University Hospital Trust, Nottingham, UK*
15. *Department Obstetrics and Gynaecology, Haukeland University Hospital, Jonas Liesvei 72, 5021, Bergen, Norway*

Corresponding author: Caitlin Dean, Pregnancy Sickness Support, 19G Normandy Way, Bodmin, Cornwall, UK; telephone number +44(0)1209 311832; email c.r.dean@amsterdamumc.nl

Word count: 3623

Objective: There are many uncertainties surrounding the etiology, treatment and sequelae of Hyperemesis Gravidarum (HG). Prioritising research questions could reduce research waste, helping researchers and funders direct attention to those questions which most urgently need addressing. The HG Priority Setting Partnership was established to identify and rank the top 25 priority research questions important to both patients and clinicians.

Methods: Following the James Lind Alliance (JLA) methodology, an HG Priority Setting Partnership (PSP) steering group was established. Stakeholders representing patients, carers and multidisciplinary professionals completed an online survey to gather uncertainties. Eligible uncertainties related to hyperemesis gravidarum. Uncertainties on nausea and vomiting of pregnancy and those on complementary treatments were not eligible. Questions were verified against the evidence. Two rounds of prioritisation included an online ranking survey and a one-day consensus workshop.

Results: 1,009 participants (938 patients/carers, 118 professionals with overlap between categories) submitted 2,899 questions. Questions originated from participants in 26 different countries, and people from 32 countries took part in the first prioritisation stage. 66 unique questions emerged, which were evidence checked according to the agreed

For peer review only

protocol. 65 true uncertainties were narrowed via an online ranking survey to 26 unranked uncertainties. The consensus workshop was attended by 19 international patients and clinicians who reached consensus on the top ten questions for international researchers to address. More patients than professionals took part in the surveys but were equally distributed during the consensus workshop. Participants from developing countries noted that the priorities may be different in their settings.

Conclusions: By following the JLA method, a prioritised list of uncertainties relevant to both HG patients and their clinicians has been identified which can inform the international HG research agenda, funders and policy makers. While it is possible to conduct an international PSP, results from developed countries may not be as relevant in low-middle income countries.

Strengths and limitations of this study

- The research process was driven by patients and healthcare professionals with a specific interest in hyperemesis gravidarum.
- Participants from a wide range of countries participated in the first truly international James Lind Priority Setting Partnership.
- Results may be less relevant to researchers within developing nations where education and awareness may be more important priorities.
- There was cross over between stakeholder groups so that many healthcare professionals and carers were also patients, therefore healthcare professionals may have been underrepresented.

Protocol

As supplementary file 1 and available at: http://www.jla.nihr.ac.uk/priority-setting-partnerships/hyperemesis-gravidarum/

INTRODUCTION

Hyperemesis gravidarum (HG) is severe vomiting and nausea in pregnancy. Affecting between 1 and 3% of all pregnancies (1), HG presents the major reason for hospital admissions in the first half of pregnancy. Yet, HG's pathophysiology has not been elucidated. Historically, HG has been an under-researched condition and the quality of HG research to date has been poor (2, 3).

A number of systematic reviews (2, 3) have shown that there is insufficient evidence to provide a definitive recommendation on the best way to treat HG. These reviews also concluded that the overall quality of evidence for HG treatments was low, and that further research was therefore required (3). The authors of the later review made several research recommendations in order of priority. However, neither of these systematic reviews engaged meaningfully with patients who had experienced hyperemesis gravidarum. As a result, the research recommendations have been criticised by patient advocacy groups for this condition (4, 5).

For peer review only - http://bmjopen.bmj.com/site/about/guidelines.xhtml
For peer review only

All too often questions being addressed by the research community are not aligned with the priorities of patients or healthcare professionals who seek answers to guide practice and decision making (6). In 2018, NHS England and the National Institute for Health Research (NIHR) conducted a needs assessment for medical research which highlighted that new research should only be undertaken where there is a defined evidence gap and a need which can be clearly articulated (7). The James Lind Alliance (JLA) is a non-profit initiative which seeks to address this mismatch by bringing patients, carers and clinicians together into Priority Setting Partnerships (PSP) to identify and prioritise the unanswered questions which they consider to be most important. Established in 2004, the JLA is funded by the NIHR and the PSPs to oversee the process with their established methodology and over 100 PSPs have been established since inception (8).

The HG PSP came together following the 2nd International Colloquium on Hyperemesis Gravidarum (ICHG) held in the United Kingdom in October 2017. During this event, leading HG researchers as well as patients from around the world agreed that setting Research Priorities for the condition was itself a priority and motivation for such a project was strong. It was noted that many of the topics relevant to patients are completely absent from the current literature (9), a mismatch noted by other JLA initiatives (6). Following this event, a subgroup of speakers and delegates with established international links to others in the field agreed to pursue the project and formed the initial PSP.

The HG PSP sought to identify the unanswered questions about HG treatment from patient and clinical perspectives and then prioritise those that patients and clinicians agree are the most important.

The objectives of the HG PSP were to:

- work internationally with patients and clinicians to identify uncertainties about the effects of HG treatments and management which have not yet been answered by existing research,
- determine by international consensus a prioritised list of those uncertainties, to guide future research, and
- to publicise the results of the PSP among researchers, research commissioning bodies and the general public in order to stimulate research in these areas.

METHODS

Patient and Public Involvement

People affected by HG, including patients, their carers and adult offspring were involved throughout this project from inception in line with the JLA methodology. The lead researcher, CD, is a patient representative and along with the patient representatives on the steering group ensured patient and carers were equal partners throughout the process including decision making around design, piloting surveys, participation and interpretation of results. The online HG patient community has been instrumental in disseminating research and will be utilised again to ensure broad public dissemination of the results from this project.

Steering group

For peer review only - http://bmjopen.bmj.com/site/about/guidelines.xhtml

The HG PSP steering group was established to oversee the project in accordance with the JLA guidelines (8). The steering group was chaired by a facilitator from JLA. Members consisted of both patients and clinicians from a range of professions involved in the care and treatment of HG, see supplementary File 2 for a list of members.

Due to the international nature of the project meetings were held online. The project ran from the first steering group meeting on the 21st March 2018 and cumulated with the final steering group meeting on 21st November 2019. The steering group's tasks were to provide input and consensus on the protocol and the survey, defining the scope of HG symptoms eligible, as well as the scope of the outcomes eligible for inclusion. Furthermore, the steering group monitored the progress of the project, provided input on the interpretation of participant feedback, and monitored the quality of the conclusions. All steering group decisions were reached by consensus.

Steering group meetings required the participation of at least three of each stakeholder groups to proceed.

Protocol, definitions, scope

The steering group developed and agreed on the study protocol (Supplementary file 1) and formally adopted terms of reference, see figure 1. The steering group agreed that no formal definition of HG would be applied, such as hospital admission; people who considered themselves to have experienced HG (or their partners/offspring/carers) would be welcome to participate. Participation would not be limited by time since experience of the condition. However, questions specifically regarding mild nausea and vomiting of pregnancy or "morning sickness" would be considered out of scope. It was unanimously agreed that the only other criteria that would render questions to be out of scope were those regarding complementary and alternative therapies.

Collection of research questions - Initial survey

Uncertainties were collected using an online survey (via SurveyMonkey®), which was also made available on paper. The survey was piloted by the steering group members. Online dissemination of the survey followed an agreed social media strategy utilising the various partner organisation's platforms. Those steering group members who distributed paper copies within clinics accepted responsibility for collecting completed surveys and returning them to the lead researcher. Posters advertising the survey with a weblink and QR code were produced and distributed to relevant clinical setting where either steering group members or other engaged stakeholders were able to display them.

Participants could submit up to five questions regarding any aspect of HG research such as diagnosis, prognosis, treatment and the organisation of care. The survey platform could only be used once on any one device. The survey was translated into both Dutch and Norwegian and paper versions of both were made available online and pre-printed. Information provided at the start of the survey about how and why to take part was also provided in video format (Viewable at: https://vimeo.com/285975872). The relevant ethics waivers were obtained for each hospital setting and a Health Research Authority certificate of non-research activity provided where required (Supplementary File 3).

For peer review only - http://bmjopen.bmj.com/site/about/guidelines.xhtml

After the first two weeks of data collection, the demographics of respondents were reviewed by the steering group members, in order to allow advertising targeted at underrepresented stakeholder groups.

Participation in this stage of the project was not a prerequisite to participation in later stages such as the interim ranking and final workshop.

Processing the data

Once the initial survey was closed, the raw data was downloaded. Foreign language versions on either paper or online were translated independently by both a healthcare professional and a patient representative for each language.

Respondents were allocated unique numbers and their raw questions separated into unique entries with the corresponding demographic information retained with each question.

The lead researcher thematically coded all responses, including out of scope questions (See supplementary file 4 for codes). Queries were discussed within the steering group until consensus was reached. To ensure consistency of coding, the steering group checked the first 100 questions and, if this gave rise to significant disagreement, the number of questions to be checked could be expanded.

Indicative questions were formed which captured the meaning of the original questions in a logical and complete question. Each raw question was allocated to the indicative question which represented it. The steering group was divided into small groups to cross-check each raw question against the allocated indicative question to ensure that they were fully represented in lay language, and original meaning was not lost or skewed during the coding/question development process.

Evidence checking

In order to identify the responses for which enough evidence of sufficient quality was available to consider them as answered i.e. "unknown knowns", an extensive check of the evidence was undertaken according to an agreed protocol (supplementary file 5). Two reviewers, CD and HB, labelled the search results according to the question references related to and CD then cross checked each question against the evidence and the RCOG and ACOG guidelines for a suitably high level of evidence identified, i.e. systematic review with conclusive results.

The steering group convened to discuss the evidence for each question. We considered a question sufficiently answered if a systematic review and meta-analysis in the last 10 years had provided a summary statistic with a narrow confidence interval, which would make it unlikely to change with increased sample size.

Interim Ranking Survey

To narrow the long list down for the final workshop, an interim online survey was conducted where participants read each question individually and scored its importance on a scale of 1-10 where 1 was not at all important and 10 was the most important. Information was

For peer review only - http://bmjopen.bmj.com/site/about/guidelines.xhtml

given at the start of the survey to encourage participants to use the full scale and to continue through the 65 questions. Several steering group members piloted the survey and comments were incorporated before launch. The questions appeared in a random order to each participant. The survey was only made available in English, however it was distributed internationally. Partial completions were not excluded.

The survey was initially open from 19th August to 18th September 2019 when a review of the demographic data was conducted. Following the review, the survey remained open until the 30th September 2019 in order to increase representation of healthcare professionals.

Consensus Workshop

Potential participants for the final consensus workshop were invited via email. Specifically, participants of the earlier surveys who had requested further information on the project were emailed as well as registered delegates for the International Colloquium on Hyperemesis Gravidarum (ICHG). Additionally, the workshop was advertised on social media through charity partner platforms and directly via email and word of mouth to steering group member contacts.

The workshop was facilitated by the JLA representative, the lead researcher (CD) was present as an observer. Participants were divided into three groups and across two sessions were switched around so that the balance of groups was always evenly distributed with an equal mix of patients and professionals. The participants were presented with all 26 indicative questions, in conjunction with the rank the indicative question had gained after the survey round. Healthcare, patient and carer ranks were separately presented to workshop participants. During the first two sessions the various groups ranked the questions 1-26 and the results were collated by the facilitators in order to reach an agreed consensus on the top 26 (8). The final session of the day saw the whole group convene, discuss any disputes regarding order, and make the final decision.

RESULTS

Uncertainty harvesting survey

The survey was open from 12th September – 16th November 2018, during which period two key healthcare professional conferences (General Practice and Early Pregnancy events) were attended and paper copies were distributed amongst attendees.

1009 participants took part in the survey, although not all participants completed all the demographic questions (between 774-964/1009, depending on the question). Missing data did not affect the eligibility or interpretation of the results. Of those who did answer any of the questions, 12.3% (n=118/963) were healthcare professionals and 97% (n=938/963) were patients and/or carers. Of the healthcare professionals who took part, 37% (n=44/118) had also suffered the condition themselves. Most participants identified as women (n=934). Only 30 people who identified as men took part of which 16 were healthcare professionals, 14 were carers (1 was both carer and professional), and 1 had been diagnosed with hyperemesis themselves. The UK and the Netherlands had the most engagement however questions were submitted from across the globe, see figure 2.

For peer review only - http://bmjopen.bmj.com/site/about/guidelines.xhtml

Initial Survey results

After removing blank and non-answers (n=19), a total of 2899 raw questions were received and coded. Of these, 37 questions were considered out of scope, 33 related to complementary therapies and four were out of scope for other reasons; one suggested a class action law suit on behalf of HG patients, two were requests to take part in research and one was excluded because no member of the steering group understood the abbreviations in the submitted question.

Indicative questions

We developed 66 indicative questions, some of which had markedly more submissions than others, for example, "what is the cause?" (n=282), "which drugs are most effective?" (n=253) and "what is the chance it will reoccur in a subsequent pregnancy?" (n=212). As the indicative questions were developed, the types of participants (patient, healthcare professional, carer, offspring) who asked each question were recorded and can be viewed in supplementary file 6.

Evidence check

The search strategy revealed 627 references, which were labelled as to which question they related to by two reviewers. Each indicative question was then checked against the search results, the two identified guidelines (RCOG and ACOG) and the Cochrane Library, for whether it could be considered answered by systematic review.

The question "does the sex of the fetus effect the occurrence/severity of the condition" was removed following the evidence check as it was deemed to be sufficiently answered by a systematic review; there is a slight increased ratio of female to male fetus in HG pregnancies (OR 1.27; 95% CI 1.21–1.34, based on x=2,672,040) (10). Five questions were considered to have partial answers, such as the role of ketoanalysis in diagnosis and management of HG (11), none were considered to be sufficiently answered that no further research is required. In all 18 questions had no references related to them while one question, complications of HG, had 136 references the majority of which were case reports. The results of the evidence check with references per question is viewable in Supplementary file 6.

Interim ranking survey

The interim survey contained all 65 indicative questions. 1,115 participants took part in the ranking survey with 524 (47%) people ranking every question. Because the survey was displayed in a random order, all questions were ranked between 603-608 times each. Healthcare professionals represented 9% of participants and were particularly hard to engage with despite efforts to inform them about the survey and encourage participation. Carers accounted for 3.6% of participants and there were notable differences in their priorities to those of patients and healthcare professionals, for example carers prioritised questions regarding symptom control for the sufferer over questions regarding harm to the fetus. International spread of participants was good with 32 countries represented.

To adjust for the low healthcare professional involvement, the question ranks were analysed and sorted by participant type. The first 21 questions were included with an

For peer review only - http://bmjopen.bmj.com/site/about/guidelines.xhtml

additional 4, that healthcare professionals had ranked above these 21, to create a top 25. However, this resulted in one question which had previously been included by patient and healthcare professionals, and which was considered acutely important by the steering group without being sufficiently covered by other questions, to be eliminated. Therefore, 26 shortlisted questions went to the workshop for final ranking.

Consensus workshop

19 participants attended from 9 countries; The Netherlands, UK, USA, Australia, Germany, Sweden, Nigeria, Ireland, Italy. There were 10 healthcare professionals and 9 patient representatives. There was good consensus between the groups and, in the final session, the whole group came together and agreed the top ten until there was unanimous agreement. The top 26 questions are shown in order of priority in Table 1.

Table 1 - Top 26 ranked priorities for hyperemesis gravidarum research

Ranking	Question
1	Can we find a cure? What novel or new treatments are being developed/tested/used elsewhere which could have a curative effect and to address all the symptoms of HG rather than just the vomiting?
2	How can we most effectively manage HG? What clinical support measure is most important to people who have had hyperemesis and what did they find most beneficial? e.g. medical management, pharmaceutical review, nutrition support, rehydration, psychological support
3	What causes hyperemesis gravidarum?
4	Is HG preventable? What is the effect of preventative treatment or early intervention on the severity and duration of HG in a subsequent pregnancy?
5	What are the immediate and long-term effects of HG (including malnutrition and dehydration, stress) on the developing foetus (offspring)?
6	What are the immediate and long-term effects of the various medications/treatments on the developing foetus (offspring) throughout the various stages of pregnancy and in varying doses or combinations of treatments?
7	What are relative efficacies of the current medications and treatment options available? What is the optimal dose, route, timing and combination of the medications and what are the related side effects?
8	What are the immediate and long term, physical, mental and social consequences and complications of HG (including malnutrition and dehydration) on the pregnant person's body? (ie. Metabolic impact, DVT, depression, effects of dehydration)
9	What clinical measurements and markers are most useful in assessing, diagnosing, managing and monitoring hyperemesis?
10	What are the nutritional requirements of the 1st, 2nd and 3rd trimesters and how can people with HG achieve these goals? i.e. Oral supplements, fortifying food, dietary measures
11	How can symptoms of HG, other than vomiting, be effectively treated? For example, the nausea, excessive saliva, extreme sense of smell and fatigue.
12	Why are some cases of HG unresponsive to all antiemetics and how can we treat such cases?
13	What is the risk that HG will reoccur in a subsequent pregnancy? Does HG get progressively worse with subsequent pregnancies and what are the risk factors for reoccurrence?
14	Do clinical treatment guidelines for HG improve management and outcomes? And if so, how can guidelines be developed and implemented nationally (where none exist) and internationally for hospital and community settings? What should be included in guidelines?
15	How can people with a history of, or significant risk factors for HG be supported to plan for a pregnancy and does such planning improve outcomes? What should a pre-pregnancy plan contain?

For peer review only - http://bmjopen.bmj.com/site/about/guidelines.xhtml

16	How does HG impact on a person's (and their family's) quality of life? How does quality and efficacy of treatment impact that effect?
17	What is the currently level of knowledge about HG and its treatments among healthcare professionals (particularly GPs)? How can effective education for healthcare professionals be designed and delivered to improve the general knowledge and awareness of HG among HCPs?
18	What is the most effective IV rehydration regime; which solution in what quantity over what time period and how frequently? Does regular rehydration improve symptoms/outcomes/quality of life?
19	What is the effect of HG on mental health during (and after) pregnancy? What is the efficacy of psychotherapy on symptom management/pregnancy outcomes/quality of life? How can people access psycho-supportive services during pregnancy?
20	What are the barriers to taking/prescribing medication for HG? How can the risk and benefits of HG and its treatments be better communicated to support informed decision making and consent to treatment?
21	What are the barriers to accessing treatments/services and how can we reduce them to improve access?
22	What healthcare services exist and how can they collaborate and be organised to better identify, treat and support people with HG? For example, do services such as outpatient clinics or IV at home, improve outcomes and reduce the physical/mental burden of the condition?
23	How can the condition be effectively managed in the community to prevent lengthy hospital admissions?
24	What self-management and coping strategies and treatments do people with HG find most helpful?
25	What employment rights do people with HG have and what financial support is available to them?
26	Do specific specialist healthcare professional roles for conditions such as HG improve outcomes? How can such roles be developed for midwives/nurses/doctors?

One participant from Nigeria expressed concern that the top ten reflected the priorities for developed, high income nations and that in low-middle income countries the priorities would be different to those reflected here. It was agreed that, despite the international nature of the project, the report would reflect that these priorities are not representative of low-middle income country (LMIC) priorities and the exercise would need to be repeated with participants representative of those countries.

DISCUSSION

The top priorities for HG research, reached by stakeholder consensus, demonstrate the lack of knowledge about fundamental aspects of HG including effective treatment, and prognosis. Our results can be used by researchers to identify future research priorities that are most relevant to patients, carers and healthcare professionals, as well as being of use to funders. The high numbers of patient responders to this PSP on HG, a transient, relatively rare condition limited to pregnancy, demonstrates the urgency felt among patients to prioritize progress in the identified research areas.

The theme of HG etiology and consequences were both highly prioritized: We don't yet have sufficient knowledge about the pathophysiology of HG or the effects of the condition on the developing fetus such as exposure to malnutrition, chronic dehydration, heightened anxiety, maternal isolation and trauma. Such information is vital for patients and clinicians to be able to make evidence based informed decisions about when and how to treat. The importance placed on identifying a cause and cure for the condition contrasts to many other JLA PSPs for conditions in which etiology and effective treatments have long been

For peer review only

established. However, common to many previous PSPs, the HG priority top questions contain general uncertainties pertaining to prevention, best management and psychological impact on those affected.

While safety evidence regarding some medications is available and plentiful, particularly of the more commonly used medications such as doxylamine/pyridoxine, which is licensed for pregnancy sickness in various countries, and off-license treatments such as ondansetron (12, 13), questions remain over efficacy, combination therapy, dose, routes of administration and almost every other aspect of treating with these medications. The lack of robust information about the consequences of HG itself has led prescribers to assume that not treating is safer than treating (14-16). In recent years researchers have made a start identifying the risks of HG, including associations with autistic spectrum disorders among offspring (17), cardiometabolic disturbances (18) and poor birth outcomes (19). However, this is not sufficient to fully inform the important and difficult decisions required by patients and their healthcare providers during management of this condition.

While healthcare professional involvement was generally lower than patient involvement across JLA projects, healthcare professionals in this project only accounted for 12% and 9% of participants in the two survey rounds. By comparison, the acne treatments PSP achieved 28% healthcare professional participation at the harvesting stage, and 64% at the ranking stage (20). The liver glycogen storage disease PSP achieved 35% at both stages (21). However, in comparison with other women’s health and pregnancy conditions, engagement was similar. The miscarriage PSP had 1093 participants in round one, of which only 9.5% were healthcare professionals; the endometriosis PSP achieved 20% healthcare professional participation, of which 15% had experienced the condition themselves (22). Likewise, as in this project, the cross over between healthcare professionals and patients was notable in other women’s health conditions, such as 11% in the preterm birth PSP (23) and 31% in the pessary use for prolapse PSP (24). The high level of patient engagement was anticipated by the authors as the patient population for this condition has previously demonstrated a strong interest for taking part in research with hundreds or even thousands of responses on surveys in a matter of days (25, 26). Although the low level of health care professional engagement was disappointing, it is not anticipated to negatively impact the goal of the PSP: to increase relevant research. Low healthcare professional engagement in pervious PSPs did not affect research stimulation: both ‘sight loss’ and ‘tinnitus’ PSPs have reported impressive funding stimulation and research uptake in the years following their PSPs (27, 28) despite low healthcare professional engagement; 17% in the sight loss PSP (29) and 11-19% in the various stages of the tinnitus PSP (30). The lack of specialist healthcare professionals roles for certain conditions may account for some of the variation in engagement, for example, are no specific HG specialist nurse or midwife roles which may have affected engagement compared to other JLA projects such as diabetes, dermatology, and stroke which have nurse and physician specialists. Alternatively, the low healthcare professional engagement in the HG PSP may reflect a more general lack of interest in, or knowledge of HG among healthcare disciplines involved in HG care.

This PSP was the second fully international PSP so far and representation was obtained from a large number of countries. However, high income countries were significantly over-represented and, despite efforts to engage doctors and patients from LMICs via social media

For peer review only

and personal contacts, there was little engagement in the survey stages and only one representative at the final workshop. By comparison, the only other international PSP for liver glycogen storage disease had three representatives (27%) from LMIC (exclusively South American countries) at its workshop (21). This is likely due to lack of funding although, for this project, travel bursaries were available including full bursaries for participants from LMICs. The JLA does not currently have a protocol for establishing how representative an international PSP should be or indeed how internationally relevant Priority Lists can be adopted in other countries. This can lead to significant research effort waste. For example, a PSP established a top ten priority list for gestational diabetes in Canada in 2017 using JLA methodology (31), yet a UK specific group is conducting a JLA for diabetes in pregnancy this year (32). Within our workshop, it was noted that the top ten priorities may not be relevant for LMICs. While repeating this process in similar high-income countries would be considered wasteful, repeating the prioritisation process of the identified uncertainties in LMICs may be a worthwhile project.

CONCLUSION

Following the JLA method, the international research arena can now direct resources to the top ten priority research questions, which will have the greatest impact for people affected by HG and the healthcare professionals caring for them.

Funding:
Funding was received from Pregnancy Sickness Support for the James Lind Alliance facilitator.
The final workshop was funded by the Bikkja Trust who also provided some bursaries for attendance at the workshop.

Author Contributions:
CD and RCP were the lead researchers, instigating the application to the JLA. CD organised the steering group meetings, designing and building the surveys, analysed the data, conducted the evidence check and wrote the majority of the final manuscript under supervision of RCP. PE (JLA Chair) chaired all the meetings, lead the consensus workshop and ensured compliance with methodology throughout. KL took minutes for steering group meetings, built and distributed surveys, organised the consensus workshop and acted as a facilitator in the workshop. RS conducted the searches for the evidence check and HB screened the results with CD. Members of the steering committee RC, BC, RG, NG, KMG, MM, DM, CN-P, MOH, HP, KS, JT and EW all attended a majority of the meetings, agreed the initial protocol and the evidence check protocol, piloted and signed off the surveys and disseminated them, checked the raw questions against the indicative ones, reviewed the evidence check results and agreed the final longlist. CD, RCP, KL, PE, RG, NG, KMG, MM and MOH were present at the consensus workshop. All authors reviewed and contributed to the final manuscript and approved it prior to submission.

Competing interests:
CD is chair of the charity Pregnancy Sickness Support, which funded the JLA aspect of this project. RG and MOH are trustees, KL is an employee and EW is a volunteer for Pregnancy Sickness Support. CD and CN-P are trustees of the Bikkja Trust which funded the consensus

For peer review only - http://bmjopen.bmj.com/site/about/guidelines.xhtml

workshop and provided some travel bursaries. CN-P has received consultancy and speaker fees from Alliance Pharma.

Data sharing statement:
All survey data and submitted questions will be available via the James Lind Alliance website without access restrictions (http://www.jla.nihr.ac.uk/priority-setting-partnerships/hyperemesis-gravidarum/)

REFERENCES

1. Einarson TR, Piwko C, Koren G. Quantifying the global rates of nausea and vomiting of pregnancy: a meta analysis. *J Popul Ther Clin Pharmacol* 2013;20(2):e171-83.
2. Boelig RC, Barton SJ, Saccone G, *et al*. Interventions for treating hyperemesis gravidarum. *Cochrane Database Syst Rev* 2016 May 11;(5): DOI: 10.1002/14651858.CD010607.pub2.
3. O'Donnell A, McParlin C, Robson SC, *et al*. Treatments for hyperemesis gravidarum and nausea and vomiting in pregnancy: a systematic review and economic assessment. *Health Technol Assess* 2016;20(74):1-268.
4. Chapman S. Hyperemesis gravidarum: sufferers need support, compassion and better research. *Evidently Cochrane* 2016. http://www.evidentlycochrane.net/hyperemesis-gravidarum-sufferers-need-support-compassion-and-better-research/ Assessed May 2020
5. Dean C, Goddard S. Patient and Public Involvement in Designing an Online Survey about Hyperemesis Gravidarum. *MIDIRS Midwifery Digest* 2016;26(3):283-90.
6. Crowe S, Fenton M, Hall M, *et al*. Patients', clinicians' and the research communities' priorities for treatment research: there is an important mismatch. *Res Involv Engagem*. 2015;1(2). https://doi.org/10.1186/s40900-015-0003-x
7. NHS England, National Institute for Health Research. NHS England's Research Needs Assessment 2018. Southampton: NHS England; 2018. p. 19.
8. The James Lind Alliance. The James Lind Alliance Guidebook Version 6. National Institute for Health Research; 2016.
9. Painter RC. Need for Research Priorities in Hyperemesis Gravidarum. International Conference on Hyperemesis Gravidarum; 2017; Windsor, UK.
10. Veenendaal MV, van Abeelen AF, Painter RC, *et al*. Consequences of hyperemesis gravidarum for offspring: a systematic review and meta-analysis. *Bjog*. 2011;118(11):1302-13.
11. Niemeijer MN, Grooten IJ, Vos N, *et al*. Diagnostic markers for hyperemesis gravidarum: a systematic review and metaanalysis. *Am J Obstet Gynecol*. 2012;211(2):150.e1-.e15.
12. Kaplan YC, Richardson JL, Keskin-Arslan E, *et al*. Use of ondansetron during pregnancy and the risk of major congenital malformations: A systematic review and meta-analysis. *Reprod Toxicol*. 2019;86:1-13.
13. Huybrechts KF, Hernández-Díaz S, Straub L, *et al*. Association of Maternal First-Trimester Ondansetron Use With Cardiac Malformations and Oral Clefts in Offspring. *JAMA*. 2018;320(23):2429-37.
14. Dean C, Murphy C. I could not survive another day: Improving treatment and tackling stigma: lessons from women's experiences of abortion for severe pregnancy sickness. London: Pregnancy Sickness Support and BPAS; 2015.

For peer review only

15. Dean C. Does the historical stigma of hyperemesis gravidarum impact healthcare professional's attitudes and treatment towards women with the condition today? A review of recent literature. *MIDIRS Midwifery Digest*. 2016;26(2):186-94.
16. Gadsby R, Barnie-Adshead T, Sykes C. Why won't doctors prescribe antiemetics in pregnancy? *BMJ*. 2011;343:d4387.
17. Getahun D, Fassett MJ, Jacobsen SJ, *et al*. Autism Spectrum Disorders in Children Exposed in Utero to Hyperemesis Gravidarum. *Am J Perinatol.* 2019.
18. Ayyavoo A, Derraik JGB, Hofman PL, *et al*. Hyperemesis gravidarum and long-term health of the offspring. *Am J Obstet Gynecol*.210(6):521-5.
19. Fiaschi L, Nelson-Piercy C, Gibson J, *et al*. Adverse Maternal and Birth Outcomes in Women Admitted to Hospital for Hyperemesis Gravidarum: a Population-Based Cohort Study. *Paediatr Perinat Epidemiol*. 2018;32(1):40-51.
20. Layton A, Eady EA, Peat M, *et al*. Identifying acne treatment uncertainties via a James Lind Alliance Priority Setting Partnership. *BMJ Open*. 2015;5(7):e008085.
21. Peeks F, Boonstra WF, de Baere L, *et al*. Research priorities for liver glycogen storage disease: An international priority setting partnership with the James Lind Alliance. *J Inherit Metab Dis*. 2019.
22. Horne AW, Saunders PTK, Abokhrais IM, *et al*. Top ten endometriosis research priorities in the UK and Ireland. *The Lancet*. 2017;389(10085):2191-2.
23. Oliver S, Uhm S, Duley L, *et al*. Top research priorities for preterm birth: results of a prioritisation partnership between people affected by preterm birth and healthcare professionals. *BMC Pregnancy Childbirth*. 2019;19(1):528.
24. Lough K, Hagen S, McClurg D, *et al*.. Shared research priorities for pessary use in women with prolapse: results from a James Lind Alliance Priority Setting Partnership. *BMJ Open*. 2018;8(4):e021276.
25. Dean CR, O'Hara ME. Ginger is ineffective for hyperemesis gravidarum, and causes harm: an internet based survey of sufferers. *MIDIRS Midwifery Digest*. 2015;25(4):449-55.
26. Nana M, Dean C, Bevan G, *et al*. Severe hyperemesis gravidarum increases rates of termination of pregnancy and suicidal ideation: results from a UK questionnaire completed by >5000 participants. International Colloquium Hyperemesis Gravidarum; 2019 10th October 2019; Amsterdam, Netherlands: ICHG.
27. Fight for Sight. Report on the funding recieved for top priority research following the James Lind Alliance PSP 2019 Available from: https://www.fightforsight.org.uk/our-research/sight-loss-priority-setting-partnership/funded-research/. Accessed May 2020
28. British Tinnitus Association. Five Years Later: What happened after the James Lind Alliance Tinnitus Priority Setting Partnership. Sheffield: British Tinnitus Association; 2018.
29. Rowe F, Wormald R, Cable R, *et al.* The Sight Loss and Vision Priority Setting Partnership (SLV-PSP): overview and results of the research prioritisation survey process. *BMJ Open*. 2014;4(7):e004905.
30. Hall DA, Mohamad N, Firkins L, *et al*. Identifying and prioritizing unmet research questionsfor people with tinnitus: the James Lind Alliance Tinnitus Priority Setting Partnership. *Clinical Investigation*. 2013;3(1):21-8.
31. Rees SE, Chadha R, Donovan LE, et al. Engaging Patients and Clinicians in Establishing Research Priorities for Gestational Diabetes Mellitus. 2017;41(2):2352-3840.
32. Diabetes and Pregnancy Priority Setting Partnership. Protocol Version 1.0. 2019. https://www.npeu.ox.ac.uk/downloads/files/jla-psp/FINAL1.0_JLA-PSP-Diabetes-and-Pregnancy-Protocol.pdf Accessed May 2020

For peer review only - http://bmjopen.bmj.com/site/about/guidelines.xhtml

Figure Captions:

Figure 1 - the JLA process with steps completed to date

Figure 2 - Country of residence of participants in the initial survey

Legend for figure 2:

- 61.82%
- 15.80%
- 6.68%
- 5.09%
- 2.44%
- 0.95%
- 0.64%
- 0.42%
- 0.32%
- 0.21%
- 0.11%

For peer review only

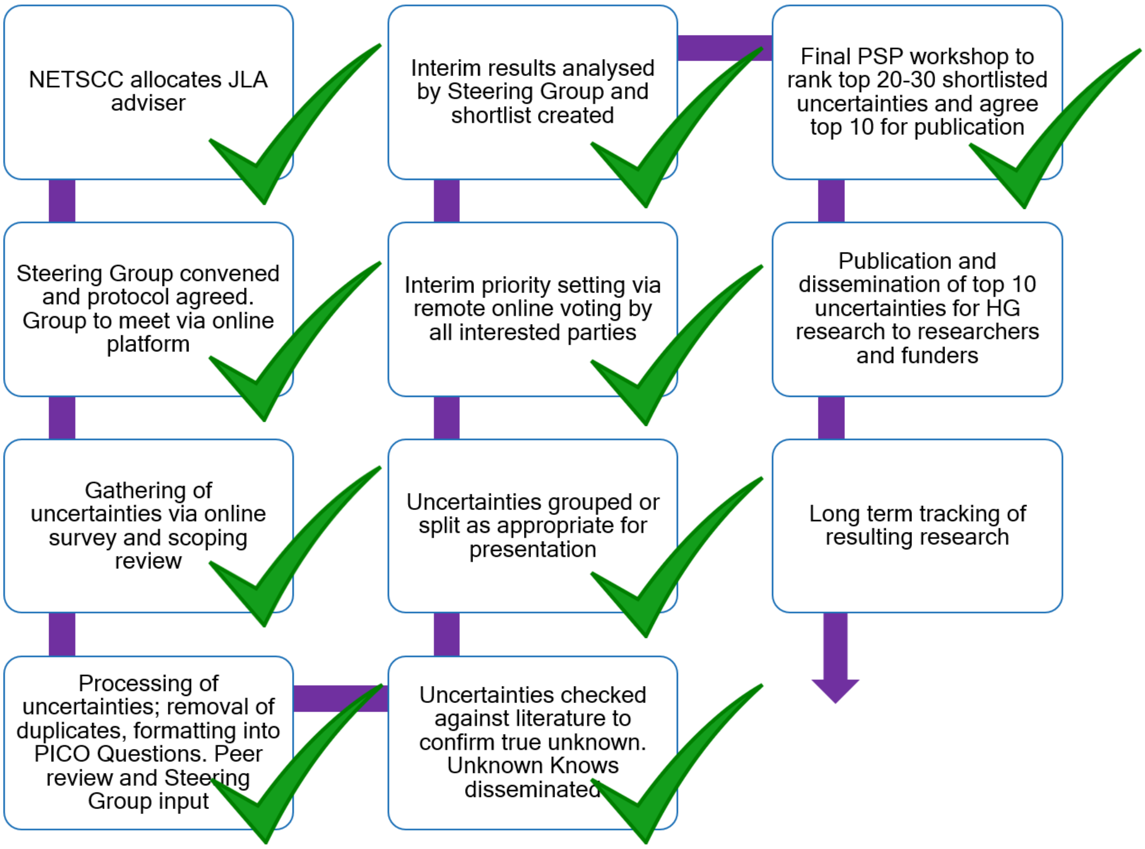

Figure 1 - the JLA process with steps completed to date

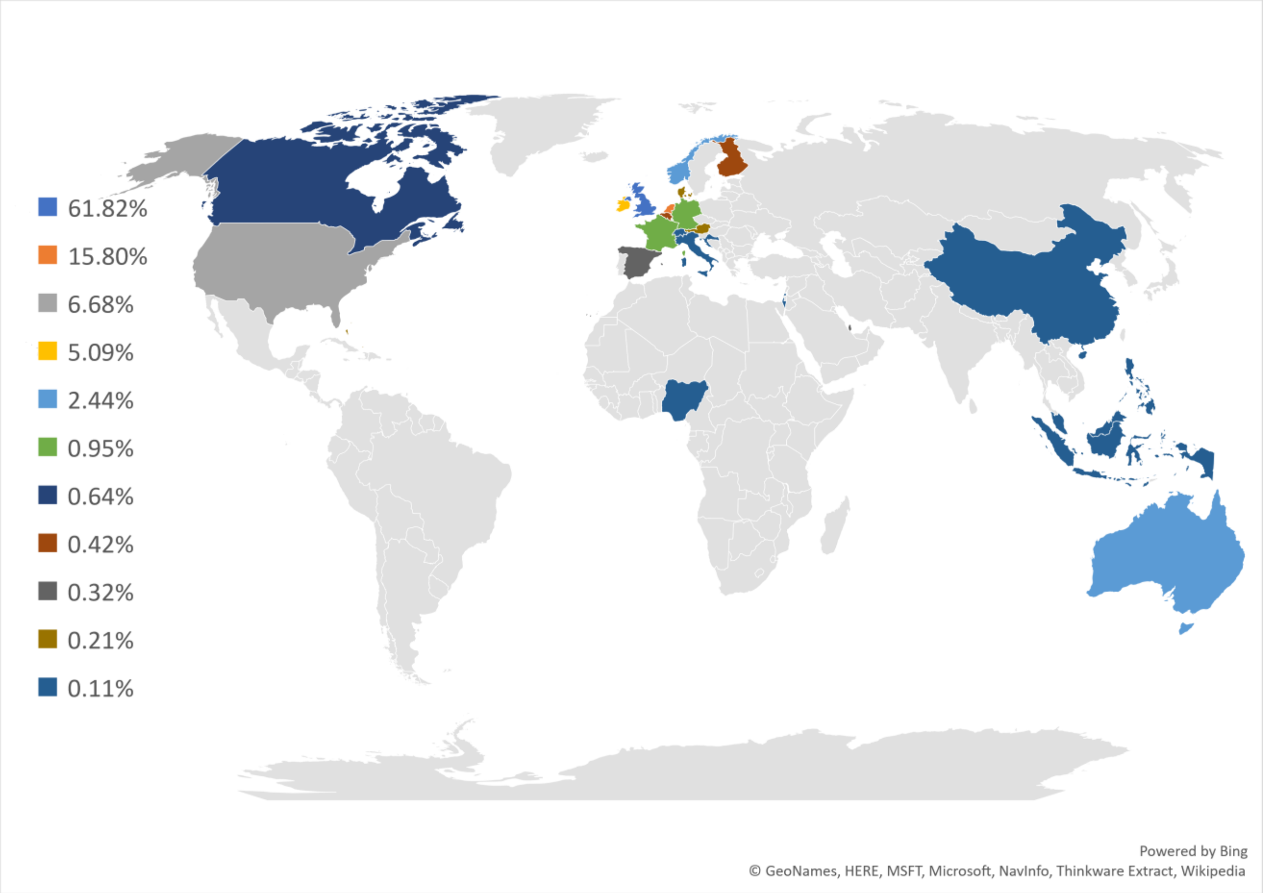

Figure 2 - Country of residence of participants in the initial survey

A JAMES LIND ALLIANCE PROJECT

HYPEREMESIS GRAVIDARUM PRIORITY SETTING PARTNERSHIP - PROTOCOL

PROTOCOL DRAFT 27/02/2018

PURPOSE OF THE PSP AND BACKGROUND

The purpose of this protocol is to set out the aims, objectives and commitments of the Hyperemesis Gravidarum (HG) Priority Setting Partnership (PSP) (HG PSP) and the basic roles and responsibilities of the partners therein. It is recommended that the Protocol is reviewed by the Steering Group and updated on at least a quarterly basis.

The James Lind Alliance (JLA) is a non-profit initiative, established in 2004. It brings patients, carers and clinicians together in Priority Setting Partnerships (PSPs). These partnerships identify and prioritise uncertainties, or 'unanswered questions', about the effects of treatments or other aspects of the condition, such as diagnosis, prognosis and the organisation of care, that they agree are the most important. The aim of this is to help ensure that those who fund health research are aware of what really matters to both patients and clinicians. The National Institute for Health Research (NIHR – www.nihr.ac.uk) funds the infrastructure of the JLA to oversee the processes for priority setting partnerships, based at the NIHR Evaluation, Trials and Studies Coordinating Centre (NETSCC), University of Southampton.

The HG PSP has come together following the 2nd International Colloquium on Hyperemesis Gravidarum held in the UK in October 2017. During this event leading HG researchers as well as patients from around the world agreed that setting Research Priorities for the condition was itself a priority and motivation for such a project was strong. Following this event, a subgroup of speakers and delegates with established international links to others in the field agreed to pursue the project and formed the initial PSP.

Historically, HG has been an under-researched condition and the quality of HG research to date has been poor (Boelig et al. 2016; McParlin et al. 2016). In part efforts have been hampered by a lack of international definition of HG and core outcome set for research, thus rendering much of the data too heterogenetic for meta-

For peer review only - http://bmjopen.bmj.com/site/about/guidelines.xhtml

analysis (Grooten et al. 2015). Thankfully these barriers are being tackled and an internationally agreed definition and core outcome set are anticipated in 2018 (ref conference).

A Cochrane review in 2016 (Boelig et al. 2016) concluded that there was insufficient evidence to provide a definitive recommendation on the best way to treat severe nausea and vomiting in early pregnancy. A simultaneous systematic review concluded that the overall quality of evidence for HG treatments was low and that further research was therefore required (O'Donnell et al. 2016). In particular the authors of the review made a number of research recommendations in order of priority. However, neither of these systematic reviews engaged meaningfully with patients who have experienced hyperemesis gravidarum and as a result the research recommendations have been criticised by patient advocacy groups for this condition (Chapman 2016) (email communication with HTA and public engagement exercises).

An increase in public and professional interest in HG in recent years and recognition of the long-term consequences for offspring has led to increase in funding and a flurry of research proposals. There is also a strong motivation among women affected by HG to take part in research and in patient and public involvement in the development of HG research (Dean and Goddard 2016).

AIMS AND OBJECTIVES OF THE HG PSP

The aim of the HG PSP is to identify the unanswered questions about HG treatment from patient and clinical perspectives and then prioritise those that patients and clinicians agree are the most important.

The objectives of the HG PSP are to:

- Together with patients and clinicians, work internationally to identify uncertainties about the effects of HG treatments and management which have not yet been answered by existing research
- Determine by international consensus a prioritised list of those uncertainties, to guide future research
- To publicise the results of the PSP among researchers, research commissioning bodies and the general public in order to stimulate research in these areas

THE STEERING GROUP

The HG PSP will be led and managed Caitlin Dean (CD) with the following steering group:

**Patient Representatives**	Margaret O'Hara (MOH)<br>Emma Watford (EW)	Pregnancy Sickness Support (PSS) (United Kingdom (UK) charity)

For peer review only - http://bmjopen.bmj.com/site/about/guidelines.xhtml

	Norah Gauw (NG)	The Netherlands
	Karina Fee (KF)	Hyperemesis Ireland
	Kimber MacGibbon (KMG)	Hyperemesis Education and Research (HER) Foundation (United States (US) and international charity)
**Clinical representatives**	Dr Rebecca Painter (RP)	Amsterdam Medical Centre
	Prof Catherine Nelson-Piercy (C N-P) (Obstetric Physician)	Professor of obstetric medicine and consultant obstetric physician, Guy's and St Thomas' Hospital
	Dr Brian Cleary (BC)(Pharmacist)	Rotunda Hospital, Dublin
	Marian MacBride (MM) (Dietician)	Rotunda Hospital, Dublin
	Kate Shorter (KS) (Nurse, HG Day Unit)	Nottingham University Hospital Trust
	Deirdre Munro (DM) (Midwife)	Global Village of Midwives Network
	Dr Roger Gadsby (RG) (General Practitioner)	Royal College of General Practitioners + experience of running a JLA PSP
	Jone Trovik (JT) (Ob/Gyne)	Haukeland University Hospital, Bergen, Norway
	Ria Clarke (RC) (Ob/Gyne)	Junior Doctor, UK
	Helen Penny (HP) (Psychologist)	Cardiff University
**The James Lind Alliance (JLA)**	Patricia Ellis (PE)	James Lind Alliance

For peer review only - http://bmjopen.bmj.com/site/about/guidelines.xhtml

The Steering Group includes representation of patient/carer groups and clinicians[1].

The Steering Group will agree the resources, including time and expertise that they will be able to contribute to each stage of the process. The JLA will advise on this.

THE WIDER PARTNERS

Organisations and individuals will be invited to be involved with the PSP as partners. Partners are groups or individuals who will commit to supporting the PSP by disseminating the PSP survey and helping the PSP to gather questions and uncertainties of practical clinical importance relating to the treatment and management of the health problem in question. Partners represent the following groups:

- people who have had HG
- carers and offspring of people who have had HG
- medical doctors, nurses and professionals allied to medicine with clinical experience of HG.

It is important that all organisations which can reach and advocate for these groups should be invited to become involved in the PSP. The JLA Adviser will take responsibility for ensuring the various stakeholder groups are able to contribute equally to the process.

EXCLUSION CRITERIA

Some organisations may be judged by the JLA or the Steering Group to have conflicts of interest, which may be perceived to adversely affect those organisations' views, causing unacceptable bias, for example, pharmaceutical companies or individual stakeholders in such companies. As such conflicts of interest would be likely to affect the ultimate findings of the PSP, those organisations will not be invited to participate. It is possible, however, that interested parties may participate in a purely observational capacity when the Steering Group considers it may be helpful.

THE METHODS THE PSP WILL USE

[1] In some cases, it has been suggested that researchers are represented at this level, to advise on the shaping of research questions. However, researchers cannot participate in the prioritisation exercise. This is to ensure that the final prioritised research questions are those agreed by patients, carers and clinicians only, in line with the JLA's mission.

For peer review only - http://bmjopen.bmj.com/site/about/guidelines.xhtml

The methods adopted in any stage will be agreed through consultation between the Steering Group members, guided by the PSP's aims and objectives.

STEP 1: IDENTIFICATION AND INVITATION OF POTENTIAL PARTNERS

Potential partner organisations will be identified through a process of peer knowledge and consultation, through the Steering Group members' networks. Potential partners will be contacted and informed of the establishment and aims of the HG PSP and may be invited to participate in an online discussion.

People wishing to participate will be able to register using an online form with a convenient email address for future communication via mailchimp. Details of which stakeholder group they identify as and which organisation they represent will be gathered.

Initial organisations will include:

Patient Charities:	Pregnancy Sickness Support
	HER Foundation
	Hyperemesis Ireland
	ZEHG (Netherlands organisation)
	Hyperemesis Gravidarum Facebook Group (10,000 + members)
	Hyperemesis Gravidarum Norway
	NB. A number of countries have budding organisations and informal patient networks, such as Australia and Germany. Representatives from each country we are aware of will be contacted. Additionally, the HER Foundation has at least one contact in most countries worldwide.
Clinician Networks:	Royal College of Obstetricians and Gynaecologists
	Royal College of General Practitioners
	Royal College of Midwives
	Royal College of Nursing Midwifery Forum and Women's Health Forum
	Association of Early Pregnancy Units
	American College of Obstetricians and Gynaecologists
	Dutch NVOG
	Dutch O&G Nurses
	"RCOG" and "RCM" equivalents for as many countries as possible will

For peer review only - http://bmjopen.bmj.com/site/about/guidelines.xhtml

be sought out and approached.

STEP 2: INITIAL STAKEHOLDER INTRODUCTION

The initial stakeholder information sheet for those registering interest in participation will have several key objectives:

- To welcome and introduce potential members of the HG PSP
- To present the proposed plan for the PSP
- To initiate discussion, answer questions and address concerns
- To invite potential partner organisations which will commit to the PSP to identify themselves and identify individuals who will be those organisations' representatives and the PSP's principal contacts
- To establish principles upon which an open, inclusive and transparent mechanism can be based for contributing to, reporting and recording the work and progress of the PSP.

This will be done via email using the emails previously registered. A forum within the project website can be used to initiate discussion, answer questions and address concerns.

STEP 3: IDENTIFYING TREATMENT UNCERTAINTIES

Each partner will disseminate an online survey to its members to solicit questions and uncertainties of practical clinical importance relating to the treatment and management of HG. A period of 8 weeks will be given to complete this exercise. Online surveys have been found to be an effective way of engaging with women affected by HG (Dean and Goddard 2016) and for collecting research priorities (Gill et al. 2013).

Predominately treatment uncertainties will be collected using an online questionnaire however the methods may be designed according to the nature and membership of each organisation, but must be as transparent, inclusive and representative as practicable. Methods may include membership meetings, email consultation, postal questionnaires, internet message boards and focus group work.

Existing sources of information about treatment uncertainties for patients and clinicians will be searched. These can include question-answering services for patients and carers and for clinicians; research recommendations in systematic reviews and clinical guidelines; protocols for systematic reviews being prepared and registers of ongoing research. A separate protocol for this search will be written.

STEP 4: REFINING QUESTIONS AND UNCERTAINTIES

The consultation process will produce "raw" unanswered questions about diagnosis and the effects of treatments. These raw questions will be assembled, categorised and refined by CD and RP into "collated

For peer review only - http://bmjopen.bmj.com/site/about/guidelines.xhtml

indicative questions" which are clear, addressable by research and understandable to all. Similar or duplicate questions will be combined where appropriate and the questions prepared for prioritisation. The following stages outlined by the JLA PSP Data Management Guide (James Lind Alliance 2016) will be followed:

Stage 1: Downloading the survey data

Survey responses from survey monkey will be downloaded and managed in an Excel spreadsheet. Additional uncertainties identified through other methods, ie. Literature search, discussion groups etc. will be added into the spreadsheet with their source documented. Each respondent and their demographic data will be identified with a unique identifier which will be attached to each of their submissions where one person submitted multiple responses. Personal identifying details will then be removed to anonymise the data.

Stage 2: Remove out of scope submissions

The scope of this PSP will need to be determined by the steering group in advance *(see minutes of steering group meeting 2 – scope). Submissions which fall outside of the scope or are established to not be uncertainties will be removed and kept separately with associated detail such as number of each stakeholder group that submitted the question and their demographics. These exclusions will be reviewed by the steering group to confirm the exclusion and decisions regarding what to do with them will be made (ie. Passing to other organisations or feeding back to the participants)

Stage 3: Categorising eligible submissions

Submissions will be categorised using the existing taxonomy by the UK Clinical Research Collaboration: Health Research Classification System (UK Clinical Research Collaboration 2009).

Stage 4: Format the submissions

Submissions will be rephrased to clarify the precise uncertainty. Where possible the "Patient/Population, Intervention, Comparison, Outcome" (PICO) format will be used. Where this is not possible uncertainties will be formatted in a way that will be useful and valuable to the research community. Lay language will be used.

Submissions which cannot be standardised will be listed and referred to the steering group for discussion. Where possible they may be combined with other eligible submissions. Formatted questions will be recorded with their frequency of submission by each stakeholder group (ie. How many times it was submitted by patients and clinicians).

Stage 5: Verifying the uncertainties

Systematic reviews and guidelines will be identified and checked by CD to see to what extent these refined questions have, or have not, been answered by previous research, specifically, up-to-date systematic review or

For peer review only - http://bmjopen.bmj.com/site/about/guidelines.xhtml

meta-analysis. Sometimes, uncertainties are expressed that can in fact be resolved with reference to existing research evidence - ie they are "unrecognised knowns" and not uncertainties. These 'answerable questions' will be recorded and dealt with separately from the 'true uncertainties' considered during the research priority setting process. If a question about treatment effects can be answered with existing information but this is not known, it suggests that information is not being communicated effectively to those who need it.

Uncertainties which are not adequately addressed by previous research will be collated and recorded on a template supplied by the JLA by CD. The list will be reviewed and agreed by the steering group.

Evidence checking protocol as agreed by Steering Group on 15th July 2019

HYPEREMESIS GRAVIDARUM PRIORITY SETTING PARTNERSHIP

EVIDENCE CHECKING PROTOCOL

This summary sets out the approach to the evidence checking process for the Hyperemesis Gravidarum Priority Setting Partnership (HG PSP), including the specific role of the Steering Group (SG) and what will be done with the findings.

THE PURPOSE OF EVIDENCE CHECKING

Systematic reviews and guidelines will be identified and checked by Caitlin Dean (CD) to see to what extent the refined questions, generated from the raw survey data, have, or have not, been answered by previous research. Sometimes, uncertainties are expressed that can in fact be resolved with reference to existing research evidence - ie they are "unrecognised knowns" and not uncertainties. If a question about treatment effects can be answered with existing information but this is not known, it suggests that information is not being communicated effectively to those who need it. Whereas uncertainties not adequately addressed by previous research will go forward to the next stage for prioritisation.

THE PROCESS FOR EVIDENCE CHECKING

Searches

A scoping search of Embase and Medline was conducted in August 2018 by information specialist Rene Spijker as part of a wider evidence mapping project running concurrently with the HG PSP. The search strategy sought to find all papers about HG without limitations. Embase, Medline were search for the key

For peer review only - http://bmjopen.bmj.com/site/about/guidelines.xhtml

terms hyperemesis gravidarum/ or ("Excessive vomiting" or (pernicious adj3 vomiting) or hyperemesis) and (gravid* or pregn* or gestation or antenatal)). Additionally, for this evidence check, searches will cover the Cochrane Database of Systematic Reviews, the Cochrane Controlled Trials Register and trip Database for Guidelines. The NICE, RCOG and ACOG guidelines will be checked for references with a suitably high level of evidence (ie. Systematic review within 10 years).

The results will been screened and labelled as to which question number they relate to by two researchers independently.

Each question will be checked against the labelled results for systematic reviews with conclusive results which directly answer the question.

Additionally, the Cochrane Library and the RCOG and ACOG guidelines will be checked for each question for a suitably high level of evidence with a definitive recommendation/answer.

Excluding NICE

NICE guidelines will not be used for evidence checking for two reasons;

1. Statements and recommendations are not referenced in a way that can be cross checked for level of evidence.
2. The website states: *"This CKS topic is largely based on the Royal College of Obstetricians and Gynaecologists guideline: The management of nausea and vomiting of pregnancy and hyperemesis gravidarum [RCOG, 2016], the American College of Obstetricians and Gynecologists Practice Bulletin: Nausea and vomiting of pregnancy [ACOG, 2015], and review articles."* These references are already being check as stated above.

10 year inclusion

Due to the paucity of HG research and lack of funding the SG felt that a three-year limit on SRs, as recommended by the James Lind Alliance, was too limited. Therefore, SRs within the last 10 years will be deemed acceptable.

Final outcome

Uncertainties which are not adequately addressed by previous research will be collated and recorded on a template supplied by the JLA (see Appendix A) by CD and will go onto the next stage for prioritisation.

For peer review only - http://bmjopen.bmj.com/site/about/guidelines.xhtml

UNKNOWN KNOWNS

Questions which are identified as already answered, but which people are unaware are already answered, ie. Unknown knowns, need to be translated into accessible information for the general public. CD will write a general article outlining the questions we have answers to and the answers themselves (not scientific) in lay language for dissemination to the general public via the main international charities, blogs and newsletters. Additionally, authors of guidelines such as the RCOG and NICE will be informed of the findings to ensure they are included in updates to the guidelines (if they are not already).

FURTHER INFORMATION

The JLA Guidebook provides step-by-step guidance to establishing and managing PSPs. You can find the Guidebook at www.jla.nihr.ac.uk

STEP 5: PRIORITISATION – INTERIM AND FINAL STAGES

The aim of the final stage of the priority setting process is to prioritise through consensus the identified uncertainties relating to the treatment or management of HG. This will be carried out by members of the Steering Group and the wider partnership that represents patients and clinicians. The stages will depend on the number of true uncertainties identified.

If there are more than 100 the Steering group will need to do an initial prioritisation to reduce the list to a more manageable number (max 80). Criteria for consideration include:

- Was the question suggested by both patients and clinicians?
- How many people suggested the question from each stakeholder group and in total?
- Was the question submitted by different kinds of clinicians or internationally?
- Whether the question overlaps with other ones or research recommendations

Interim Priority Setting

This will be conducted as an online survey. Questions will be scored and ranked by participants and the process tracked with relevant details. From here approx. 25 priorities will be retained for the final priority setting meeting.

Final Priority setting

For peer review only

For peer review only - http://bmjopen.bmj.com/site/about/guidelines.xhtml

The final stage to identify 10 prioritised uncertainties will take place at the ICHG Conference in Amsterdam in October 2019. The JLA will facilitate this process and ensure transparency, accountability and fairness. Participants will be expected to declare their interests in advance of this meeting.

DISSEMINATION OF FINDINGS AND RESEARCH

FINDINGS AND RESEARCH

The data will be submitted to the JLA for publication on its website on completion of the priority setting exercise, taking into account any changes made at the final workshop, in order to ensure that PSP results are publicly available.

It is anticipated that the findings of the HG PSP will be reported to funding and research agenda setting organisations such as the NIHR and the major research funding charities. Steering Group members and partners are expected to develop the prioritised uncertainties into research questions, and to work to establish the research needs of those unanswered questions to use when approaching potential funders, or when allocating funding for research themselves, if applicable. It is anticipated that Pregnancy Sickness Support will aim to provide modest grants for researchers addressing priority questions.

PUBLICITY

In addition to an academic paper a simple language report will be produced by the partner organisations and a PR strategy implemented.

A review of research resulting from the priority list will be presented at ICHG 2021 in Dublin and priority given at that conference to presentations and posters addressing the priority questions.

REFERENCES

Boelig RC, Barton SJ, Saccone G, Kelly AJ, Edwards SJ, Berghella V. 2016. Interventions for treating hyperemesis gravidarum. Cochrane Database Syst Rev 5:DOI: 10.1002/14651858.CD010607.pub2.

Chapman S. 2016. Hyperemesis gravidarum: sufferers need support, compassion and better research. Evidently Cochrane.

Dean C, Goddard S. 2016. Patient and Public Involvement in Designing an Online Survey about Hyperemesis Gravidarum. MIDIRS Midwifery Digest 26(3):283-290.

For peer review only - http://bmjopen.bmj.com/site/about/guidelines.xhtml

Gill FJ, Leslie GD, Grech C, Latour JM. 2013. Using a web-based survey tool to undertake a Delphi study: application for nurse education research. Nurse Educ Today 33(11):1322-8.

Grooten I, Roseboom T, Painter R. 2015. Barriers and Challenges in Hyperemesis Gravidarum Research. Nutr Metab Insights 8(Suppl 1):33-9.

James Lind Alliance. 2016. JLA PSP Data Management Guide. Southampton: James Lind Alliance.

McParlin C, O'Donnell A, Robson SC, Beyer F, Moloney E, Bryant A, Bradley J, Muirhead CR, Nelson-Piercy C, Newbury-Birch D et al. . 2016. Treatments for Hyperemesis Gravidarum and Nausea and Vomiting in Pregnancy: A Systematic Review. JAMA: Journal of the American Medical Association 316(13):1392-1401.

O'Donnell A, McParlin C, Robson SC, Beyer F, Moloney E, Bryant A, Bradley J, Muirhead C, Nelson-Piercy C, Newbury-Birch D et al. . 2016. Treatments for hyperemesis gravidarum and nausea and vomiting in pregnancy: a systematic review and economic assessment. Health Technol Assess 20(74):1-268.

UK Clinical Research Collaboration. Health Research Classification System [Internet]. London: UK Clinical Research Collaboration; 2009. Available from: http://www.hrcsonline.net/sites/default/files/HRCS_Document.pdf

For peer review only

For peer review only - http://bmjopen.bmj.com/site/about/guidelines.xhtml

Supplementary File 2:

Members of the HG PSP Steering Commitee

**Patient Representatives**	Margaret O'Hara (MOH)	Pregnancy Sickness Support (PSS) (United Kingdom(UK) charity)
	Emma Watford (EW)	Pregnancy Sickness Support (PSS) (United Kingdom(UK) charity)
	Norah Gauw (NGT)	ZEHG, The Netherlands
	Karina Fee (KF)	Hyperemesis Ireland
	Kimber MacGibbon (KMG)	Hyperemesis Education and Research (HER) Foundation (United States (US) and international charity)
**Clinical representatives**	Dr Rebecca Painter (RP) (Ob/Gyne)	Amsterdam University Medical Centres (and ZEHG Charity)
	Prof Catherine Nelson-Piercy (C N-P) (Obstetric Physician)	Professor of obstetric medicine and consultant obstetric physician, Guy's and St Thomas' Hospital
	Prof Brian Cleary (BC)(Pharmacist)	Rotunda Hospital, Dublin (and Hyperemesis Ireland Charity)
	Marian MacBride (MM) (Senior Dietician)	Health Service Executive, Dublin (and Hyperemesis Ireland Charity)
	Katherine Shorter (KS) (Nurse, HG Day Unit)	Nottingham University Hospital Trust
	Deirdre Munro (DM) (Midwife)	Global Village of Midwives Network, Portiuncula University Hospital, Co. Galway Ireland (and Hyperemesis Ireland Charity)
	Dr Roger Gadsby (RG) (General Practitioner)	Royal College of General Practitioners + experience of running a JLA PSP and PSS UK Charity
	Prof Jone Trovik (JT) (Ob/Gyne)	Haukeland University Hospital, Bergen, Norway
	Dr Ria Clarke (RC) (Ob/Gyne)	Junior Doctor, UK
	Dr Helen Penny (HP) (Clinical Psychologist)	Cardiff University
**The James Lind Alliance (JLA)**	Patricia Ellis (PE)	James Lind Alliance

For peer review only

Go straight to content.

MRC Medical Research Council

NHS Health Research Authority

Is my study research?

To print your result with title and IRAS Project ID please enter your details below:

Title of your research:

Hyperemesis Gravidarum Research Priority Setting Partnership with The James Lind Alliance

IRAS Project ID (if available):

N/A

You selected:

- **'No'** - Are the participants in your study randomised to different groups?
- **'No'** - Does your study protocol demand changing treatment/ patient care from accepted standards for any of the patients involved?
- **'No'** - Are your findings going to be generalisable?

Your study would NOT be considered Research by the NHS.

You may still need other approvals.

Researchers requiring further advice (e.g. those not confident with the outcome of this tool) should contact their R&D office or sponsor in the first instance, or the **HRA** to discuss your study. If contacting the HRA for advice, do this by sending an outline of the project (maximum one page), summarising its purpose, methodology, type of participant and planned location as well as a copy of this results page and a summary of the aspects of the decision(s) that you need further advice on to the HRA Queries Line at **HRA.Queries@nhs.net**.

For more information please visit the **Defining Research** table.

Follow this link to start again.

Print This Page

NOTE: If using Internet Explorer please use browser print function.

About this tool **Feedback** **Contact** **Glossary**

For peer review only - http://bmjopen.bmj.com/site/about/guidelines.xhtml

Supplementary file 4

Data codes

Codes for types of participants

Code	Demographic question response
P	I was diagnosed with HG during pregnancy or I had HG during pregnancy but was not diagnosed
C	I cared for my partner/daughter/sister/friend when she had HG
O	My mother had HG during her pregnancy with me (offspring)
H	I am a health or social care professional caring for women with HG
ORG	I am part of an organisation representing people with HG and their families

Codes for raw questions prior to establishing indicative questions

OOS	Out of scope;<br>1 = alternative therapy<br>2 = other
C	Cause
H	Hereditability
R	Recurrence
MMA	Markers/Measurements/Assessment tool incl. clinical predictors
RF	Risk factors
DC	Disease characteristics incl. prevalence, duration, symptoms
T<br>TM<br><br><br><br><br><br><br>TG<br>TO<br>TIV<br>TN<br>TP<br>TA<br>TSO<br>TMM<br>TL<br>TT<br>TSAT	Treatment<br>Treatment - Medication;<br>1 = efficacy<br>2 = safety<br>3 = development of new ones<br>4 = combining/polypharmacy<br>5 = side effects<br>6 = administration (route/dose/timing)<br>Treatment guidelines/protocols<br>Treatments - other<br>Treatment - Intravenous fluid<br>Treatment – nutritional/nutrition<br>Treatment - Prevention<br>Treatment - Access<br>Treatment service organisation<br>Treatment – Marijuana<br>Treatment -Licensing<br>Treatment – Termination<br>Treatment - Satisfaction
S	Support<br>1 = psychological<br>2 = holistic<br>3 = family/carer
CS	Coping Strategies
CCO	Consequences, Complications, outcomes, other symptoms<br>1 = foetal risks/consequences incl. long term

For peer review only

	2 = maternal risks/consequences incl. long term<br>3 = mental health during pregnancy<br>4 = mental health after pregnancy<br>5 = quality of life<br>6 = for family
ED	Education<br>1 = of HCPs<br>2 = public/others
POST	Post HG, recovery, trauma
ECON	Economic questions
METH	Methodology specified
A	Attitudes/Beliefs/Stigma/Awareness
OTHER	Other (for further discussion with Steering Group)

For peer review only

A JAMES LIND ALLIANCE PROJECT

HGPSP PRIORITY SETTING PARTNERSHIP

James Lind Alliance Priority Setting Partnerships

HYPEREMESIS GRAVIDARUM PRIORITY SETTING PARTNERSHIP

EVIDENCE CHECKING PROTOCOL

This summary sets out the approach to the evidence checking process for the Hyperemesis Gravidarum Priority Setting Partnership (HG PSP), including the specific role of the Steering Group (SG) and what will be done with the findings.

THE PURPOSE OF EVIDENCE CHECKING

Systematic reviews and guidelines will be identified and checked by Caitlin Dean (CD) to see to what extent the refined questions, generated from the raw survey data, have, or have not, been answered by previous research. Sometimes, uncertainties are expressed that can in fact be resolved with reference to existing research evidence - ie they are "unrecognised knowns" and not uncertainties. If a question about treatment effects can be answered with existing information but this is not known, it suggests that information is not being communicated effectively to those who need it. Whereas uncertainties not adequately addressed by previous research will go forward to the next stage for prioritisation.

THE PROCESS FOR EVIDENCE CHECKING

Searches

A scoping search of Embase and Medline will be conducted by an information specialist (René Spijker as part of a wider evidence mapping project running concurrently with the HG PSP. The search strategy will seek to find all papers about HG without limitations. Embase, Medline will be searched for the key terms hyperemesis gravidarum/ or (("Excessive vomiting" or (pernicious adj3 vomiting) or hyperemesis) and (gravid* or pregn* or gestation or antenatal)). Additionally, for this evidence check, searches will cover the Cochrane Database of Systematic Reviews, the Cochrane Controlled Trials Register and trip Database for Guidelines. The RCOG and ACOG guidelines will be checked for references with a suitably high level of evidence (ie. Systematic review within 10 years) as per the JLA methodology handbook.

The results will be screened and labelled as to which question number they relate to by two researchers independently.

Each question will be checked against the labelled results for systematic reviews with conclusive results which directly answer the question.

For peer review only

Additionally, the Cochrane Library and the RCOG and ACOG guidelines will be checked for each question for a suitably high level of evidence with a definitive recommendation/answer.

Excluding NICE

NICE guidelines will not be used for evidence checking for two reasons;

1. Statements and recommendations are not referenced in a way that can be cross checked for level of evidence.
2. The website states: *"This CKS topic is largely based on the Royal College of Obstetricians and Gynaecologists guideline: The management of nausea and vomiting of pregnancy and hyperemesis gravidarum [RCOG, 2016], the American College of Obstetricians and Gynecologists Practice Bulletin: Nausea and vomiting of pregnancy [ACOG, 2015], and review articles."* These references are already being check as stated above.

10 year inclusion

Due to the paucity of HG research and lack of funding, the SG felt that a three-year limit on SRs, as recommended by the James Lind Alliance, was too limited. Therefore, SRs within the last 10 years will be deemed acceptable.

Final outcome

Uncertainties which are not adequately addressed by previous research will be collated and recorded on a template supplied by the JLA (see Appendix A) by CD and will go onto the next stage for prioritisation.

UNKNOWN KNOWNS

Questions which are identified as already answered 'Unknown knowns', need to be translated into accessible information for the general public. CD will write a general article outlining the questions we have answers to and the answers themselves (not scientific) in lay language for dissemination to the general public via the main international charities, blogs and newsletters. Additionally, authors of guidelines such as the RCOG and NICE will be informed of the findings to ensure they are included in updates to the guidelines (if they are not already).

FURTHER INFORMATION

The JLA Guidebook provides step-by-step guidance to establishing and managing PSPs. You can find the Guidebook at www.jla.nihr.ac.uk

For peer review only - http://bmjopen.bmj.com/site/about/guidelines.xhtml

Supplementary File 6: Questions, evidence, participant categories of submissions and examples

Question rank after first prioritisation	Question code	Uncertainty	Answered?	No of refs identified from search strategy	Evidence	Total sub-missions	patients	HCPs	Carers	Off-spring	Org reps	Unkn-own	Sample of submitted questions (sic)
1	1	What causes hyperemesis gravidarum?	No	62	Various papers with new research in recent years. No definitive answer or SR yet.	282	249	28	9	31	1	14	Where does HG come from? What causes HG to begin with and why does it only affect some women? What medical studies is have been conducted to find the cause / cure for HG? Why do we get hg? What is the pathophysiology behind hyperemesis gravidarum? Why do some women get HG snd others don't? Why does one person get HG and the other don't?
11	2	What are the risk factors for developing HG and can we predict who will develop it?	No	85	Fan, L. Y.; Jacobsen, K. H. 2010 Risk factors for hyperemesis gravidarum, Current Women's Health Reviews - Volume 6, Issue 4, pp. 309-317: systematic review from 2010 identifies a few risk factors; Low pre-pregnancy weight, Helicobacter pylori infection, a history of hyperemesis gravidarum in a previous pregnancy, and carrying a female fetus but calls for further research with better definition of maternal characteristics.	96	77	21	4	12	1	7	Are there any ways to predict who will develop HG? As they learn more about HG, to learn if women could be tested thru gene testing. Is there a test being developed to determine likelihood off suffering HG? Can we use genetic testing to identify women as risk and offer preventative meds earlier? And will this lessen the severity? Is there any way of predicting if you will suffer from this?
3	3	What is the risk that HG will reoccur in a subsequent pregnancy? Does HG get progressively worse with subsequent pregnancies and what are the risk factors for recurrence?	No	8	5 papers vary from 15-81%, meta-analysis not possible.	212	191	16	10	17	0	6	What is the recurrence rate? Will I definitely have HG again in future pregnancies? Does Hyperemesis get worse with each pregnancy? What are the chances that you get HG with the following pregnancy/pregnancies? If you have HG, how likely are you to get it a second time? What are the chances of getting HG again after suffering with it during my first pregnancy? Is HG more severe dependant on partner? Are you likely to get HG again if you use sperm from a different man/donor? How much of an influence does the sperm have?
40	4	What clinical measurements and markers are most useful in assessing, diagnosing, managing and monitoring hyperemesis?	No	47	Niemeijer, M. N.; Grooten, I. J.; Vos, N.; Bais, J. M.; van der Post, J. A.; Mol, B. W.; Roseboom, T. J.; Leeflang, M. M.; Painter, R. C.; 2014 Diagnostic markers for hyperemesis gravidarum: a systematic review and metaanalysis American Journal of Obstetrics & Gynecology - Volume 211, Issue 2, pp. 150.e1-150.15: Systematic review concludes no support for the use of ketonuria in the diagnosis of HG. H	11	7	3	0	1	0	1	What anthropometric measures and nutritonal markers are most useful in assessing and monitoring hyperemesis management? (??weight, mid upper arm circumference, transferrin, amylase etc?????) What clinical indicators determine the threshold for hospital admission in women with HG? What diagnostic criteria/guidelines would provide the best treatment/outcomes? i.e. instead of waiting for significant weight loss and dehydration or “penalizing”proactive care that reduces these indicators

For peer review only

					pylori serology might be useful in specific patients.								
46	5	What objective measures can people used to know when to seek further help or hospital admission?	No	0	None	7	6	1	0	0	0	0	Self monitoring urine to know when to need help.... is this a possibility? Is there a way for the individual to self monitor how bad things are getting to be able to be proactive? How do you know when you need to go to hospital, eg what are the measures of dehydration? I remember going to hospital and being told you are not dehydrated enough for treatment following that I was reluctant to go to hospital
39	6	Can we predict the severity and duration for individual patients and which treatments they will respond to?	No	7	Some original studies identified but no systematic review or conclusive evidence	12	7	5	0	0	0	0	What factors predict severity and duration of HG in a pregnancy? Are there any clinical predictors of response to different anti-emetics in HG? Why do some HG sufferers have relief of symptoms afte 3/4 months and some are sick each day until the day they give birth? Can we predict how long HG will last? Is there any way of predicting which medications are most effective for whom?
	7	Does the sex of the foetus affect occurrence and/or severity of HG?	Yes	8	increased ratio of female to male fetus in HG pregnancies. (OR 1.27; 95% CI 1.21–1.34): MVE Veenendaal AFM van Abeelen RC Painter JAM van der Post TJ Roseboom, 2011, Consequences of hyperemesis gravidarum for offspring: a systematic review and meta-analysis. BJOG 18(11)	21	19	2	1	3	0	1	What are the chances my HG will be worse with a girl pregnancy than with a boy? Does the sex of the baby (boy/girl) make a difference in the extent of complaints of HG? Is there A difference in HG Between women who are Carrying girls vs boys?
29	8	Does Ketoanalysis have a role in the diagnosis and management of HG?	Yes and no	3	Ketoanalysis does not corrlate with severity of HG and should therefore not be used for diagnosis of HG however, this does not answer whether or not they have a role in management of HG. Niemeijer, M. N.; Grooten, I. J.; Vos, N.; Bais, J. M.; van der Post, J. A.; Mol, B. W.; Roseboom, T. J.; Leeflang, M. M.; Painter, R. C.; 2014 American Journal of Obstetrics & Gynecology - Volume 211, Issue 2, pp. 150.e1-150.15: Systematic review concludes no support for the use of ketonuria in the diagnosis of HG. Guidelines conflict but based on "good practice point/clinical experience"	21	18	3	1	1	0	0	Why do we rely on ketones to test dehydration? Can treatments be provided before you have ketones in urine instead of after? Will hospitals realise they do not have to look at ketones to diagnose dehydration in a hg patient? Why is ketosis the measure for dehydration? Can I still be dehydrated without having ketones in my urine? Why do women have to get to the point of having ketones before their condition is treated seriously?

For peer review only

For peer review only - http://bmjopen.bmj.com/site/about/guidelines.xhtml

18	9	What should the clinical definition of HG be and can it be diagnosed in a timely and consistent way?	No	1	Guidelines give level D evidence	56	51	3	0	7	0	4	At what point is your morning sickness classed as HG and is there different levels of HG ie Mild/Severe? How can we diagnose and treat patients quicker? Can hg be detected earlier in pregnancy? How can we identify hyperemesis earlier in pregnancy? What are the criteria of HG? How can Health Care Professional assess and diagnose HG earlier? How can HG be quickly diagnosed (and not dismissed as simply morning sickness)? My mother had it and was left to cope what is bad enough to get a disgnosis? How do I get diagnosed rather than told it's normal?
27	10	Is having HG a risk factor for requiring induction/c-section, miscarriage, other conditions/complications of pregnancy, or particular birth outcomes?	No	49	Multiple case-control and cohort studies identified. An SR may be able to answer this.	30	29	2	1	4	1	1	Whats the connection with HG en pregnancy diabetes? Could HG be related to any other sickness or disorder in my future, just as gestational diabetes is related with the chances of developing diabetes type 2 after pregancies? Are thyroid issues related to first having HG? Induction and C section rate amongst HG? Is there a link between hg and gestational diabetes? Is HG linked to 1gall bladder dysfunction? 2gestational diabetes 3 sluggish digestion 4 thyroid levels? Has there been any link between HG sufferers and delivery type? I.e. an increased chance of forceps delivery?
15	11	Does HG run in families? What is the level of risk of experiencing HG for a person with a family history of HG?	No	7	Multiple studies identified. An SR may be able to answer this.	74	63	6	8	10	0	2	Is HG heritable? If my mum had HG what are my chances of getting it? How likely is it to get HG if both mother and grandmother had it? Can anything specific mitigate the possibility of children experiencing hyperemesis themselves during future pregnancies, if their mother suffered hyperemesis? As I've had HG in my pregnancy what is the likelihood of a close relative suffering from the condition as well? E.g. sister or daughter. Because I had hyperemisis what are the chances of my daughter having this?
26	12	How do factors such as diet, nutrition, stress, tiredness and rest affect symptoms and coping? What can exacerbate symptoms and how can we manage those factors?	No	3	3 studies identified, each on different topics.	31	26	4	3	2	1	2	What is the effect of rest/tiredness on vomiting during pregnancy? Are there any triggers which can worsen hyperemesis? Are there and factors that aggravate HG? What factors (biological ans psychological) exacerbate HG? And how can these be managed during HG? Some foods increase phlegm/mucus and some foods increase throwing up. Is there a relation? Does fatigue/tiredness/ workload affect the nausea and vomiting exerienced? Do certain foodsubstances have more influence on HG?

For peer review only

5	13	Is HG preventable? What is the effect of preventative treatment or early intervention on the severity and duration of HG in a subsequent pregnancy?	No	1	One study which does not sufficiently answer the question	168	152	22	5	12	1	1	Is there anything the patient could have done/can do different to prevent it? What can be done to help prevent HG in those identified as at risk, I e those who have had it in previous pregnancy. What factors determine duration - do certain treatments or early intervention affect duration or is it determined by the underlying cause? What is the best way to get HG under control ASAP and limit the mental effects to the patient? If you start taking medication early on in pregnancy will this lessen symptom severity overall?
20	14	What are the disease characteristics of HG? (Prevalence, onset, duration, symptoms, peaks?)	No (and yes)	14	Some aspects answered such as prevalence, but other aspects such as onset, duration, symptoms and peaks not answered. Prevalence SR and meta analysis concluded: The prevalence of HG was 1.1% (CI95%:0.8%-1.3%), with a range of 0.3%-3.6%. Almost 70% of women worldwide experience NVP, but reported rates vary widely. HG, the most severe form, affects 1.1%. Ref: Einarson TR, Piwko C, Koren G. Quantifying the global rates of nausea and vomiting of pregnancy: a meta analysis. Journal of population therapeutics and clinical pharmacology = Journal de la therapeutique des populations et de la pharamcologie clinique. 2013;20(2):e171-83.	40	35	8	2	1	1	0	Numbers of how often HG occurs, are there any differences in different countries / parts of the world? Are there differences in the rate of HG across ethnic groups? How long is this likely to last? What is the chance of HG easing and then returning ? While suffering from H G I tend to be spitting every few seconds, can't swallow any of my saliva (spit cup necessary) any research on how long this spitting last's for? What kind of statistics are there regarding Hg? (Recurrence, who gets it, how long it will last...) Are there certain weeks when hormones always surge to make HG worse? What are the chances that this feeling will last the entire pregnancy? At what week does HG usually improve a bit? How common is it to have HG the whole pregnancy? What is the incidence of HG continueing after the first trimester and for how long does it continue?
23	15	How can symptoms of HG, other than vomiting, be effectively treated? For example, the nausea, excessive saliva, extreme sense of smell and fatigue.	No	5	Some prevalance studies on ptyalism (excessive salivation) identified. No discussion of other symptoms in guidelines	35	34	2	2	3	0	0	Is there an effective treatment for ptyalism?Is there anything to reduce the excess saliva that comes hand in hand with HG?One of the most distressing symptoms of HG for many women is the heightened sense of smell. Is there a medical way to target that symptom in particular?The sickness and nausea is obviously a big part of HG. But how do we treat the less talked about effects including extreme tiredness, no energy, headaches, dizziness, breathlessness, confusion, being unable to stand/endure movement or motion/listen to conversations/music without feeling like you are going to be sick or faint? How can the other symptoms be reduced (i.e. smell sensitivity, excessive saliva, extreme fatigue, recurring UTIs). Is there a way to control the extreme sensitivity to smell, which causes so much agony? Is there anything that can be done to dull the extremely heightened sense of smell that a lot of HG patients suffer with? As I honestly think it would have helped with the severity of the constant nausea in my case.

For peer review only

2	16	What are relative efficacies of the current medications and treatment options available? What are the optimal dose, route, timing and combination of the medications and what are the related side effects?	No	58	Cochrane review concludes: there is little high-quality and consistent evidence supporting any one intervention. Ref: Boelig RC, Barton SJ, Saccone G, Kelly AJ, Edwards SJ, Berghella V. Interventions for treating hyperemesis gravidarum. The Cochrane database of systematic reviews. 2016;5:DOI: 10.1002/14651858.CD010607.pub2. HTA SR found: there is little on the effectiveness of treatments in more severe NVP/HG. Ref: O'Donnell A, McParlin C, Robson SC, Beyer F, Moloney E, Bryant A, et al. Treatments for hyperemesis gravidarum and nausea and vomiting in pregnancy: a systematic review and economic assessment. Health Technol Assess. 2016;20(74):1-268	253	195	71	11	19	5	8	What are the options for treating it? What is the best dosage of x (insert any of treatments) for most effective results for most women? What are all the options available to women who experience HG? My doctor was very dismissive. What are the recommended first, second and third line anti-emetic drugs in HG? When to switch over to the first line to second line, and so on? Which is the highest dose of Cariban? Best regime. Why do doctors not readily prescribe combinations of anti-emetics when patient's symptoms appear unimproved with one anti-emetic. For example cyclizine and metoclopramide?
24	17	How can we most effectively manage HG? What clinical support measure is most important to women who have had hyperemesis and what did they find most beneficial? eg medical management, pharmaceutical review, nutrition support, rehydration, psychological support	No	13	Some trials, case-control and case studies for various support measures. No SR, no large well conducted trials.	34	27	6	3	5	0	1	What's the best way to manage HG? What clinical support measure is most important to women who have had hyperemesis and what did they find most beneficial? eg medical management, pharmaceutical review, nutrition support, rehydration, psychological support. What are the best treatment methods for Hyperemisis Gravidarum? What is the treatment recommendations? (Including medicines, food intake, intravenous therapy, psychological help...) How can we use the medical intervention available to us to make this disease more bearable for mothers who suffer?
17	18	Can we find a cure? What novel or new treatments are being developed/tested/used elsewhere which could have a curative effect and to address all the symptoms of HG rather than just the vomiting?	No	9	Some small and novel trials identified for gabapentin, clonidine, mitrazepam, marijuana and other therapies.	66	52	7	3	6	2	7	Is there a cure? Will there ever be a cure for this 'HG'? is there not a surgical way or medical way to eradicate this reaction? By what treatment can HG be totally cured? This means de vomitting, the nausea, lack of feeling hungry, etc. Given the 2 genes (contributing to appetite and placenta) which were recently found to be connected with the root causes of Hyperemesis Gravidarum, how can one reduce his / her chances of acquiring this sickness or cure it in an earlier stage than 20 weeks plus or the entire pregnancy? Please try to find a cure!!

For peer review only

4	19	What is the effect of HG on mental health during pregnancy? What is the efficacy of psychotherapy on symptom management/pregnancy outcomes/quality of life? How can women access psychosupportive services during pregnancy?	Yes and No	28	Systematic review and meta-analysis have shown a significantly increased frequency of depression and anxiety in women with HG. Ref: Mitchell-Jones N, Gallos I, Farren J, Tobias A, Bottomley C, Bourne T. Psychological morbidity associated with hyperemesis gravidarum: a systematic review and meta-analysis. Bjog. 2017;124(1):20-30. For interventions, various studies have assessed interventions, SR may be possible but question is not currently answered.	188	172	20	7	25	2	6	How does the mental component of HG (desperation, exhaustion, feeling that 'I can't do this for another eight months') influence the patients physical wellbeing and how do we include mental health care in the treatment? Can mental health support be provided at home for those who are unable to travel alone? How do we support women with HG before, during and after pregancy psychologicly? What mental health support is available for women and how can we improve access to this service for women who struggle to leave the house when suffering with HG? What support is available for the psychological repercussions of HG? Comparison between conventional medical treatment and psychotherapy for hyperemesis gravidarum. What are the barriers to women accessing counselling for HG? Suicidal thoughts and HG. How can this be better supported by the the community midwife and mental health team?
33	20	How can the condition be effectively managed in the community to prevent lengthy hospital admissions?	No	7	Some studies comparing ambulatory to inpatient care.	17	13	6	0	1	0	1	What is the best management or treatment to get women with HG out of the hospital and back home rather than staying in hospital? What can be done to keep HG sufferers home instead of in hospital? Can hyperemesis patients prevent themselves getting into a situation where they need to be admitted to hospital by having regular IV drips, perhaps day appointments for this or community midwifes administer this as a method of prevention/management of the condition? Are women who are car d for at home with HG more or less likely to suffer from post natal mental health issues?
25	21	Do clinical treatment guidelines for HG improve management and outcomes? And if so how can guidelines be developed and implemented nationally (where none exist) and internationally for hospital and community settings? What should be included in guidelines?	No	6	Some audits of local guidelines highlight inconsistent treatment. Effectiveness of national RCOG and ACOG guidelines have not been evaluated.	33	28	6	0	1	0	3	Would there be a benefit to developing a national best practice guidance for managing HG? can there be one HG protocol for every healthcare provider? Make a list of available medication along with safe dosage available to all medical professionals, as all too often treatment/medication given varies between hospitals, due to lack of information. Is it possible to create a worldwide guide so docters treat everybody the same. There is so much difference between country's but even between hospitals and docters.
13	22	What are the barriers to accessing treatments/services and how can we reduce them to improve access?	No	4	Four small studies/case studies identifying barriers to accessing treatment.	95	86	11	5	7	2	1	What treatment is available, how effective is each treatment and how do I access it? When partner is to ill to communicate how do I get her the best medication or know where to go for help? How can I initiate the process for home care with my insurance if homebound with HG? How can I access IV hydration at home? How can we support medical/nursing teams to access specialist mental

For peer review only

													health support for HG sufferers/families when NHS budgets are constantly being cut?
30	23	What self-management and coping strategies and treatments do people with HG find most helpful?	No	0	None identified	20	17	2	2	0	0	0	What can make women with HG feel better? I tried many thing nutricion , medication , many sleep but nothing worked out, what worked out for the best for other woman ?
34	24	What percentage of people with HG are satisfied with the care and treatment they received? How can satisfaction be increased and how can healthcare professionals best support people with HG?	No	0	None identified	17	13	3	0	2	0	1	How can we help in clinic? What percentage of women are satisfied with the treatment they receive for HG? How can quality of care be improved for women with HG? Were the midwives and doctors helpful whilst you were suffering? Which support and treatment options do women attribute to helping them have a positive pregnancy experience (eg a care plan, continuous care from a midwife, early diagnosis and treatment, help to make informed choice regarding treatment options etc)?
37	25	How can dehydration be prevented/managed without an IV drip?	No	0	None identified	13	10	2	0	1	0	1	What am I supposed to do when I can't keep liquid down to stay hydrated? Can the priority shift from treating dehydration to preventing dehydration? How dangerous is getting towards dehydration for longer periods or more often for the vascular system of the uterus and effects on the foetus?
10	26	What healthcare services exist and how can they collaborate and be organised to better identify, treat and support women with HG? For example, do services such as outpatient clinics or IV at home, improve outcomes and reduce the physical/mental burden of the condition?	No	18	Some audits, case-control studies and RCTs identify services such as Home Healthcare and outpatient services in specific areas of individual countries. SR of outpatient verse inpatient outcomes may be possible.	97	78	21	4	12	3	2	How could the healthcare system be better equipped to identify and treat hg? which reproducible model of management (inpatient day unit/ via a&e) has the best outcomes? How can consultant-led hyperemesis specialty clinics in maternity services improve co-ordination of care and outcomes for women? Do you think there is better scope for community treatment of Hyperemisis Gravidarum to improve women's lifestyles and mental health? Do women prefer inpatient or outpatient care for hyperemesis? What is the cost/impact of HG management in acute settings vs a community based model?
14	27	What other services are available/could be developed to support families affected by HG? And how can access be enabled to services which currently exist	No	2	Two studies suggest counselling and martial support may be helpful	78	70	12	6	5	0	0	Can mental health support be provided at home for those who are unable to travel alone? Is there any association of volunteers to help or support the expectant mother, husband and or older siblings in everyday life? E.g. picking up medication for mom, prepare food, taking care of kids...
19	28	How long does it take people to recover from HG? What post-pregnancy is care available (or could be developed) for people who have suffered HG during pregnancy and what is its impact on recovery?	No	2	Two studies (one survey, one case-control) looking at aftereffects and recovery identified.	50	46	1	4	9	1	1	How long does the average HG sufferer take to 'get over' the trauma of her pregnancy? Do some never get over it? What aftercare can be made readily available for women who have had hg during pregnancy? How does postpartum care need to be organised to shorten recovery?

For peer review only

47	29	Do specific specialist healthcare professional roles for conditions such as HG improve outcomes? How can such roles be developed for midwives/nurses/doctors?	No	0	None identified	7	6	2	0	1	0	0	Will there be special hg physicians in the future? Can we look into a community midwife specialising in hyperemsis? It would be nice if there is a special HG clinic or team that is specialized in HG. Would that be possible in the near future? Which doctor (gynecologist)is the best, most experienced doctor for treating Hyperemesis gravidarum in Europe?
35	30	How does HG treatment/management vary by country (and regions within countries) and how do the variations effect outcomes?	No	0	Various types of services reported in studies and guidelines from around the world. No research addressing question.	16	14	1	0	1	0	1	Why is the medical treatment different in an other country? Here in the Netherlands you can only get emesafene and sometimes metoclopramide for max 5 days. Why is the treatment in the uk not as efficient as that of the us? (I.e home care, pick lines). Why is care so different in so many areas ? Why do the treatments vary so much per hospital/country/doctor
44	31	What additional checks, tests and medical reviews should people with HG be offered during pregnancy?	No	17	Various tests proposed/highlighted in case reports and case-control studies. SR may be helpful to address.	8	8	1	0	3	0	0	Should you be seen more by consultant when pregnant with HG? How often should a doctor review the patient's condition and treatments? Why don't women with HG get more regular or routine appointments to check the healthy development of the foetus? Why are HG sufferers not offered earlier blood tests to monitor iron levels etc? when do you consider further investigations like imaging/endoscopy in cases of persistent hyperemesis beyond 16 weeks of pregnancy? why aren't women who suffer from HG monitored more closely to make sure baby's growth is going well? When you vomit many times a day for months, why aren't electrolyte levels checked?
8	32	Do negative stigmas, attitudes and beliefs surrounding HG affect the quality/consistency of care provided on individual healthcare professional and institutional levels? If so, what is the impact of such stigmas and how can they be addressed?	No	1	A number of studies identified that found persistent stigmatisation of HG among healthcare professionals in the UK. Qualitative SR may be possible	112	108	9	4	10	3	0	Why does the quality/consitency of care, in HG patients, differ so much between medical professionals? Why is the care so variable - I.e from one gp to the next? Why is it so difficult to access rapid and effective treatment? Having to fight for anti emetics and rehydration and being treated like a hysterical addict by doctors adds to the trauma of hg. Why is it so difficult to access the right medication and treatment in some areas? How can you get taken seriously by you gp when you report this, and not just brushed off as being silly? How can I explain what I'm feeling to my doctor in a way that will help me be taken seriously? What are the consequences for the patient when professionals say to them that it's a psychological issue, even if they still treat the patient physically?

For peer review only

51	33	How many people terminate their pregnancy due to HG symptoms, lack of access to treatment or other related factors? What are their experiences and how can they be supported?	No	1	Some studies identifying rate of termination but no conclusive evidence. SR may be possible.	6	6	1	1	0	1	0	How often do women terminate wanted pregnancies because the hg is too difficult? How many terminations are carried out because of lack of access to treatment for HG
53	34	When is it appropriate to induce labour early and/or conduct elective c-section due to third trimester HG?	No	0	No papers identified and no mention of induction/elective section in guidelines	4	3	0	0	0	0	1	Can I be induced at 36 weeks because of the severity of hg? If an unborn child can be delivered- healthy as early as 36 (?) weeks, why are doctors not relieving HG mothers of their symptoms who are still suffering near the end of their third trimester. If the woman with Hyperemesis Gravidarum suffers from the condition throughout the whole pregnancy and is severely anemic and weak, is elective Cesarean section recommended, that is, is that woman in greater risk of having complications enduring vaginal birth?
28	35	How can people with a history of, or significant risk factors for HG be supported to plan for a pregnancy and does such planning improve outcomes? What should a pre-pregnancy plan contain?	No	0	RCOG guidelines suggest possible benefit to prophylactic treatment but evidence is poor. No evidence for pre-pregnancy care plan.	27	24	2	2	5	0	1	Planning for a second pregnancy can be very difficult. How can barriers be broken down for more women before pregnancy ? How can we better support women who are terrified to have another child?
31	36	What is the role of vitamin supplementation during HG, which vitamins are important, how should they be administered, and do they effect outcomes?	No	36	Multiple articles addressing various vitamins particularly Vit K and B1. SR may be possible.	20	17	3	2	3	0	1	It is safe to take vitamins if I have HG? I was unable to swallow pregnancy tablets and was malnourished through being unable to eat or drink for 6 months. I received one dose of vitamins through a drip in this time. Why was I not given these regularly.
60	37	How can appointments and clinics within all possible settings (home, GP, hospital etc) be made more 'HG friendly'? (ie. Reducing sensory stimulations, smells, noise, location, access to toilet/sick bowls etc)	No	0	No studies identified	2	1	1	0	0	0	0	How can appointments and clinics within all possible settings (home, GP, hospital etc) be made more accessible and 'HG friendly'?
38	38	What are the barriers to taking/prescribing medication for HG? How can the risk and benefits of HG and its treatments be better communicated to support informed decision making and consent to treatment?	No	1	Some barriers identified but no conclusive studies within HG literature. A wider search regarding medication use in pregnancy may yield more insight which is applicable to HG?	13	11	2	0	2	0	1	The biggest worry is taking medication and any impact that may have on your baby, clear advice and information needs to be provided prior to taking any medication. I was only offered stemitil which didn't help and only learned afterwards other medication was available.
54	39	What are the barriers to medication testing and licensing for use during pregnancy and how do we try to overcome those barriers?	No	0	No results within HG literature but a wider search regarding general medication testing in pregnancy may yield more insight which is applicable to HG?	4	4	0	0	0	0	0	Need of RCTs whene it comes to effect of medications? Why are there so few medications licensed and well-tested for use for HG during pregnancy? Has odansetron become licensed for use in pregnancy yet? Can it become standard for GP's to prescribe nationwide?

For peer review only

56	40	Do personalised care plans reduce suffering and improve outcomes for people?	No	3	One RCT assessed difference between patients receiving individual holistic care package and controls receiving "normal care". No statistical difference was found. Further studies required.	3	2	1	0	1	0	0	How can action plans be written up to ensure dehydration, hospitalisation are reduced? Such as day visits for fluids, and consult led care
6	41	What is the currently level of knowledge about HG and its treatments among healthcare professionals (particularly GPs)? How can effective education for healthcare professionals be designed and delivered to improve the general knowledge and awareness of HG among HCPs?	No	5	Some studies/audits have tried to assess local current knowledge but do not address how education can be improved or delivered.	148	138	9	8	22	2	4	I think raising awareness in medical professionals is the best way to support women with HG. How can we do this? Could all hospitals and medical professionals be better educated as to how they deal with HG? Is there a process to follow? considering complications, general state, psychological state, evolution of the pregnancy, effects on patient's day to day life and for patient's surroundings: are there significant differences between patients treated by well informed professionals and patients treated by professionals who don't have particular knowledge of HG ?
32	42	What is the most effective IV rehydration regime: which solution in what quantity over what time period and how frequently? Does regular rehydration improve symptoms/outcomes/quality of life?	No	5	Lack of clear studies, current recommendations in guidelines from low level evidence.	19	12	7	1	0	0	0	Is there a difference between different electrolyte infusions? What is the best IV fluid regimen in women with HG? How effective is rapid rehydration as an outpatient treatment?
36	43	How can weight loss/malnutrition from HG be effectively managed/reduced? Do nutritional therapies such as NG tubes, TPN, PEG feeding improve outcomes?	No	15	Some studies and case reports of enteral feeding for HG with mixed results. Lack of evidence for nutritional therapies in general. ACOG guidelines vague on enteral feeding, discourages parenteral feeding with low level evidence references but lacks alternatives to managing weight loss and malnutrition	14	10	4	0	0	0	0	Whicht treatment to reduce the HG and minimize the weightloss? Is it nutrition or IV for example? What role does IV nutrition play in preventing long term health impacts for HG babies? What role does IV nutrition play in preventing premature birth?
57	44	What are the risks and benefits to using Peripherally Inserted Central Catheters (PICC) lines and central lines for people with HG requiring regular IV therapy?	No	2	ACOG and RCOG references a number of low-level evidence sources highlighting risks associated with PICC lines in pregnancy.	3	3	0	0	0	0	0	If I am going to emerge 3 or 4 times a week for IV fluids and they have trouble finding veins because of dehydration why can't they put in a port or picc earlier why must we suffer so long and get yelled at for a shitty veins ?
61	45	How should treatment vary throughout pregnancy according to physiological changes and metabolic requirements? Ie. Should doses increase later in pregnancy, are some medications less suitable close to delivery	No	0	No studies identified	2	1	0	1	0	0	0	Does choice of best medication to treat HG change with gestation?

For peer review only

55	46	How does HG impact on a person's (and their family's) quality of life? How does quality and efficacy of treatment impact that effect?	Yes and No	7	Negative effects identified in a qualitative systematic review ref: Dean C, Bannigan K, Marsden J. Reviewing the effect of hyperemesis gravidarum on women's lives and mental health. British Journal of Midwifery. 2018;26(2): Plus further studies since then add to evidence base. However, no evidence identified on how quality and efficacy of treatments mitigate or exacerbate the effect.	4	3	2	0	0	2	0	What is the range of difference in quality of life for patients as linked to the medicines they are given?
58	47	Why are some cases of HG unresponsive to all antiemetics and how can we treat such cases?	No	9	Cause of unresponsiveness not addressed in literature. Various novel therapies, enteral/parenteral feeding and termination proposed for unresponsive cases in a small number of papers and the two guidelines.	3	3	0	0	0	0	0	Why in some cases is it not possible to control the sickness and vomiting? Why doesn't medication work (good enough)? Why do different medications or combinations of medications work (or not work) for different patients? Why don't pills or medications work against the hg effects?
12	48	What are the immediate and long-term effects of the various medications/treatments on the developing foetus throughout the various stages of pregnancy and in varying doses or combinations of treatments?	No	20	Various studies and reviews of individual medications for particular exposure periods. For example, SR found: "first trimester ondansetron exposure is not associated with an overall statistically significant increased risk of congenital malformations or cardiovascular defects." ref: Balayla, J.; D'Alton, M. E. 2016 First trimester ondansetron exposure and the risk of major congenital malformations and heart defects: A systematic review and meta-analysis. American Journal of Obstetrics and Gynecology - Volume 1, Issue 0, pp. S177. This doesn't answer the question on whether ondansetron is safe throughout pregnancy, third trimester or in what doses or in combination with other medication	96	80	19	2	9	0	3	Do any of the medicines harm the baby? Does the type, dose and duration of medication have different effects on the baby depending on the time of the pregnancy the medication is used. For example, does ondansetron have a different (potential) effect on the baby if this is taken in the first trimester vs the last trimester? Is this drugs cocktail really the best thing for baby?
45	49	What dietic service support is currently available to people with HG within maternity services? What impact does dietic care have on clinical outcomes and quality of life?	No	0	No studies identified, Guidelines suggest dietician involvement based on expert opinion	8	4	5	0	0	0	0	What is women's experience of dietetic support in maternity services? What is women's experience of dietetic support in maternity services? What impact does dietetic care have on clinical outcomes and QoL for women with hyperemesis? Is there any improvement in clinical outcomes in those who recieve dietetic input versus those who do not?

For peer review only

21	50	What are the nutritional requirements of the 1st, 2nd and 3rd trimesters and how can people with HG achieve these goals? Ie. Oral supplements, fortifying food, dietary measures	No	1	None identified within HG specific literature but a search within wider pregnancy literature may identify evidence relevant to this question.	38	32	5	4	6	0	0	Is there benefit in providing hyperemesis patients with oral nutritional supplements? Which foods are best to eat when suffering from HG? What is the best way to gain and retain vital nutrients during HG? What dietary advice is best for a HG-patient?
63	51	Does physiotherapy have a role to play in HG management?	No	0	No studies identified. Guidelines suggest multidisciplinary approach	1	1	0	0	0	0	0	Would having some Physiotherapy possibly reduce the risk of blood clots and help mental health Of those suffering HG?
22	52	What are the wider biopsychosocial impacts of HG on partners, families, older children, relationships, work and finances? And how can families be supported?	No	5	some studies address wider family impacts. SR maybe possible. No studies on support for families	37	34	1	0	4	2	2	What is the impact of HG on the woman's partner/ family (e.g. having to take time off work, becoming a carer, feeling hopeless and helpless) and how does HG change relationships
7	53	What are the immediate and long term effects of HG (including malnutrition and dehydration, stress) on the developing foetus?	Yes and No	56	Some immediate effects identified in systematic review but long-term consequences require further study: Veenendaal MV, van Abeelen AF, Painter RC, van der Post JA, Roseboom TJ. Consequences of hyperemesis gravidarum for offspring: a systematic review and meta-analysis. Bjog. 2011;118(11):1302-13.	130	112	25	3	8	1	3	What are the effects/consequences of HG for the baby and the mother in long term? What are the risks to the fetus when a woman has Hyperemisis Gravidarum? What are the long term effects of a HG pregnancy on the health of a child? (Impact of medication but also, is there any impact on the child anyway without meds e.g. long term growth, IQ, physical health, mental health etc)
9	54	What are the immediate and long term, physical, mental and social consequences and complications of HG (including malnutrition and dehydration) on the pregnant person's body? (ie. Metabolic impact, DVT, depression, effects of dehydration)	No	136	Psychological morbidity has been demonstrated with systematic review: Mitchell-Jones, N., et al. (2017). "Psychological morbidity associated with hyperemesis gravidarum: a systematic review and meta-analysis." Bjog-an International Journal of Obstetrics and Gynaecology 124(1): 20-30. A myriad of other consequences and complications have been identified in the literature but no systematic review.	110	95	12	5	12	2	6	What are the most common complications of HG? How can we raise the risk of embolism, so women know when to seek help? What are the longterm effects of HG for mother and baby? Metabolic impact of hyperemesis during and after pregnancy? What are the long tern effects of HG (i.e. up to 5 years after the pregnancy)? Is HG linked to higher rates of postnatal depression, family breakdown, and decisions not to have any other children? How many HG sufferers had to stop working prematurely?
41	55	Does HG effect breastfeeding or early bonding and attachment with the baby?	No	3	Some studies looking at attachment and bonding with conflicting results. Not enough for SR. Original research required.	11	10	1	1	1	0	0	What is the impact of HG on early bonding and attachment with baby? How does HG affect breastfeeding? How does the experience of HG affect subsequent bonding and attachment between the mother and baby? Can you breastfeed through HG (i.e. breastfeed an older child -- I did but couldn't get any support or find anyone else who had done it)? Are women with HG less likely to breastfeed?

For peer review only

42	56	Does HG impact on people's reproductive choices and/or limit families? Does this have an effect on family wellbeing and mental health?	No	2	Mixed results from a couple of heterogeneous studies SR not possible yet, research needed	10	9	3	0	0	0	0	How does HG affect the likelihood of a family choosing to have another baby? Do you feel as though your experience with HG was limiting/ debilitating enough to make you not want to have another child, as opposed to if you hadn't suffered HG during your pregnancy? was HG a reason not to get any more children? Did HG stop you having further pregnancies?
48	57	How should people with comorbidities be managed while suffering HG, for example, diabetes, epilepsy, mental health conditions?	No	21	Various case reports of a variety of comorbidities with HG and a cohort study looking at diabetes. Further research needed. An SR on the case reports of gastric bands/bypass in relation to HG may be possible.	7	5	3	0	0	0	0	How do I maintain adequate blood sugar control as a patient with type 1 diabetes and hg? How can we best handle gestational diabetes when HG impacts what and when and if you can eat? If a type 1 diabetic woman gets HG what should she do? How does Syndrome Gilbert affect pregnant woman, especially one that suffers from Hyperemesis Gravidarum? How does HG effect women with current or past eating disorders? As someone that already suffers with mental health problems is there a chance hg has made things worse ?
16	58	How can awareness of the condition be raised among the public/employers/media etc? How does the public perception of the condition affect people suffering HG?	No	0	No studies identified	67	59	3	8	7	0	4	What do employers need to know in order to be able to support women with HG? The general public should be made aware of this condition - any plans to publicise this illness? Many people do not understand the severity of HG. How can it be explained to family members and employers? How can we all make the world more aware of what HG is and the horrific issues behind it?
49	59	What is the total economic cost to the individual, their family, the health system, and society when a person has HG? How do differing models of care alter this?	No	8	An SR which attempted economic analysis of treatments for HG/NVP found" The economic analysis was limited by lack of effectiveness data", Further research required. Ref: O'Donnell A, McParlin C, Robson SC, Beyer F, Moloney E, Bryant A, et al. Treatments for hyperemesis gravidarum and nausea and vomiting in pregnancy: a systematic review and economic assessment. Health Technol Assess. 2016;20(74):1-268	7	4	4	0	0	1	0	What is the total economic cost to the individual, her family, the health system and society when a patient has hyperemesis gravidarum? Explore economic burden of severe NVP/hyperemesis in different countries (direct + indirect cost). What is the cost/impact of HG management in acute settings vs a community based model. What is the estimated annual cost to the national economy of working-days lost due to HG and related conditions?
50	60	How can HG research be improved and stimulated? Which treatment outcomes are meaningful to patients, what definition of HG can be used for research, what light can qualitative research shed on woman's experiences of HG, can a database of HG offspring be established, How can RCTs	No	5	Problems with definition and outcomes identified in Systematic Review which demonstrates need for consensus on these for research: Koot, M. H., et al. (2018). "Variation in hyperemesis gravidarum definition and outcome reporting in randomised clinical trials: a systematic review." Bjog-an International Journal of Obstetrics and Gynaecology 125(12): 1514-1521.	7	2	5	0	0	0	0	Which treatment outcomes are meaningful to HG patients? Can we agree on an international definition of HG? women's experiences of severe HG qualitative research? Are the children born to HG sufferers put on any sort of database to try and collate some sort of information for future medical survey/information reference? I have a more general question, methodological. Which (Medical) interventions improve the relevant outcomes. I want to see rcts! No more or much less observAtional

For peer review only

For peer review only - http://bmjopen.bmj.com/site/about/guidelines.xhtml

		of medical treatments be encouraged and supported?											
59	61	What other diagnosis should be considered, and at which points in pregnancy/disease stage?	No	70	Many case reports of differential diagnoses at varying points in pregnancy, SR would be helpful. Guidelines list various possible differential diagnosis based on low quality evidence and expert opinion.	3	2	1	0	0	0	0	when do you consider further investigations like imaging/endoscopy in cases of persistent hyperemesis beyond 16 weeks of pregnancy
62	62	Would an alternative name/classification for hyperemesis gravidarum better reflect the scope of the condition and improve awareness/reduce stigma?	No	0	No evidence identified. It may be worth looking at literature from other condition which have been renamed to assess potential impact of such work.	2	1	1	0	0	0	1	What can we rename HG which reflects that some women don't have severe vomiting but severe nausea which leads to same outcomes
64	63	What are people's rights to treatment and how can people with HG be enabled to exercise their rights?	No	0	No literature identified.	1	1	0	0	0	0	0	What are my rights if GP is refusing to offer medicines?
52	64	What is the mode of action for the various medications?	No	0	No specific reports within the HG literature identified. Searches on specific medications within pharmacology literature may yield better results.	5	4	1	0	0	0	0	What is the action of the various medications prescribed? I've seen caraban used for HG how does this mechanism work? Why is HG better controlled on a combination of medicines rather than just one?
43	65	What employment rights do people with HG have and what financial support is available to them?	No	0	No literature identified in relation to HG, general pregnancy literature searches per country may yield more results	9	8	0	1	1	0	0	
65	66	Are there survival/evolutionary benefits to HG?	No	3	Some studies proposing theories, no SR identified.	1	0	1	0	0	0	0	Are there survival/evolutionary benefits to HG?

For peer review only

BMJ Open

A Patient-Clinician James Lind Alliance Partnership to Identify Research Priorities for Hyperemesis Gravidarum

Journal:	*BMJ Open*
Manuscript ID	bmjopen-2020-041254.R1
Article Type:	Original research
Date Submitted by the Author:	29-Sep-2020
Complete List of Authors:	Dean, Caitlin; Amsterdam UMC Location AMC, Obstetrics and Gynecology; Pregnancy Sickness Support<br>Bierma, Hyke; Amsterdam UMC Location AMC, Obstetrics and Gynecology<br>Clarke, Ria; Frimley Park Hospital NHS Foundation Trust, Obstetrics and Gynaecology<br>Cleary, Brian; Royal College of Surgeons in Ireland, School of Pharmacy; Hyperemesis Ireland<br>Ellis, Patricia; James Lind Alliance<br>Gadsby, Roger; Warwick Medical School, Institute of Clinical Education; Pregnancy Sickness Support<br>Gauw, Norah; ZEHG Foundation<br>Lodge, Karen; Pregnancy Sickness Support<br>MacGibbon, Kimber; Hyperemesis Education Research<br>McBride, Marian; Hyperemesis Ireland; Health Service Executive, Strategic Planning and Transformation<br>Munro, Deirdre; Portiuncula Hospital; Hyperemesis Ireland<br>Nelson-Piercy, Catherine; Guy's & St Thomas' Foundation Trust<br>O'Hara, Margaret; Pregnancy Sickness Support<br>Penny, Helen; Cardiff University, School of Psychology<br>Shorter, Katherine; Queen's Medical Centre Nottingham University Hospital NHS Trust<br>Spijker, René; Amsterdam UMC Location AMC<br>Trovik, Jone; Haukeland University Hospital, Obstetrics and Gynaecology<br>Watford, Emma; Pregnancy Sickness Support<br>Painter, Rebecca; Amsterdam UMC Location AMC, Obstetrics and Gynaecology
**Primary Subject Heading**:	Obstetrics and gynaecology
Secondary Subject Heading:	Research methods, Patient-centred medicine
Keywords:	Maternal medicine < OBSTETRICS, OBSTETRICS, HEALTH SERVICES ADMINISTRATION & MANAGEMENT, STATISTICS & RESEARCH METHODS

SCHOLARONE™
Manuscripts

I, the Submitting Author has the right to grant and does grant on behalf of all authors of the Work (as defined in the below author licence), an exclusive licence and/or a non-exclusive licence for contributions from authors who are: i) UK Crown employees; ii) where BMJ has agreed a CC-BY licence shall apply, and/or iii) in accordance with the terms applicable for US Federal Government officers or employees acting as part of their official duties; on a worldwide, perpetual, irrevocable, royalty-free basis to BMJ Publishing Group Ltd ("BMJ") its licensees and where the relevant Journal is co-owned by BMJ to the co-owners of the Journal, to publish the Work in this journal and any other BMJ products and to exploit all rights, as set out in our licence.

The Submitting Author accepts and understands that any supply made under these terms is made by BMJ to the Submitting Author unless you are acting as an employee on behalf of your employer or a postgraduate student of an affiliated institution which is paying any applicable article publishing charge ("APC") for Open Access articles. Where the Submitting Author wishes to make the Work available on an Open Access basis (and intends to pay the relevant APC), the terms of reuse of such Open Access shall be governed by a Creative Commons licence – details of these licences and which Creative Commons licence will apply to this Work are set out in our licence referred to above.

Other than as permitted in any relevant BMJ Author's Self Archiving Policies, I confirm this Work has not been accepted for publication elsewhere, is not being considered for publication elsewhere and does not duplicate material already published. I confirm all authors consent to publication of this Work and authorise the granting of this licence.

For peer review only

A Patient-Clinician James Lind Alliance Partnership to Identify Research Priorities for Hyperemesis Gravidarum

Caitlin Dean[1,2], Hyke Bierma[2], Ria Clarke[3], Brian Cleary[4,5], Patricia Ellis[6], Roger Gadsby[1,7], Norah Gauw[8], Karen Lodge[1], Kimber MacGibbon[9], Marian McBride[4,10], Deirdre Munro[4,11], Catherine Nelson-Piercy[12], Margaret O'Hara[1], Helen Penny[13], Katherine Shorter[14], René Spijker[2], Jone Trovik[15], Emma Watford[1], Rebecca C. Painter[2]

1. *Pregnancy Sickness Support, 19G Normandy Way, Bodmin, Cornwall, UK*
2. *Amsterdam University Medical Centres, University of Amsterdam, Meibergdreef 9, 1105 AZ Amsterdam, the Netherlands*
3. *Obstetrics and Gynaecology, Frimley Park Hospital, Surrey, UK*
4. *Hyperemesis Ireland, Dublin, Ireland*
5. *School of Pharmacy, Royal College of Surgeons in Ireland, Dublin, Ireland*
6. *James Lind Alliance, Southampton, UK*
7. *Warwick Medical School, University of Warwick, UK*
8. *ZEHG Foundation, The Netherlands*
9. *Hyperemesis Education and Research Foundation, Damascus, Oregon USA*
10. *Strategic Planning and Transformation, Health Service Executive, Dublin, Ireland*
11. *Portiuncula University Hospital Galway, Ireland*
12. *Guy's & St Thomas' Hospital, London, UK*
13. *School of Psychology, Cardiff University, Cardiff, UK*
14. *QMC, Nottingham University Hospital Trust, Nottingham, UK*
15. *Department Obstetrics and Gynaecology, Haukeland University Hospital, Jonas Liesvei 72, 5021, Bergen, Norway*

Corresponding author: Caitlin Dean, Pregnancy Sickness Support, 19G Normandy Way, Bodmin, Cornwall, UK; telephone number +44(0)1208 872801; email c.r.dean@amsterdamumc.nl

Word count: 3820

Objective: There are many uncertainties surrounding the etiology, treatment and sequelae of Hyperemesis Gravidarum (HG). Prioritising research questions could reduce research waste, helping researchers and funders direct attention to those questions which most urgently need addressing. The HG Priority Setting Partnership was established to identify and rank the top 25 priority research questions important to both patients and clinicians.

Methods: Following the James Lind Alliance (JLA) methodology, an HG Priority Setting Partnership (PSP) steering group was established. Stakeholders representing patients, carers and multidisciplinary professionals completed an online survey to gather uncertainties. Eligible uncertainties related to hyperemesis gravidarum. Uncertainties on nausea and vomiting of pregnancy and those on complementary treatments were not eligible. Questions were verified against the evidence. Two rounds of prioritisation included an online ranking survey and a one-day consensus workshop.

Results: 1,009 participants (938 patients/carers, 118 professionals with overlap between categories) submitted 2,899 questions. Questions originated from participants in 26 different countries, and people from 32 countries took part in the first prioritisation stage. 66 unique questions emerged, which were evidence checked according to the agreed

For peer review only

protocol. 65 true uncertainties were narrowed via an online ranking survey to 26 unranked uncertainties. The consensus workshop was attended by 19 international patients and clinicians who reached consensus on the top ten questions for international researchers to address. More patients than professionals took part in the surveys but were equally distributed during the consensus workshop. Participants from developing countries noted that the priorities may be different in their settings.

Conclusions: By following the JLA method, a prioritised list of uncertainties relevant to both HG patients and their clinicians has been identified which can inform the international HG research agenda, funders and policy makers. While it is possible to conduct an international PSP, results from developed countries may not be as relevant in low-middle income countries.

Strengths and limitations of this study

- The research process was driven by patients and healthcare professionals with a specific interest in hyperemesis gravidarum.
- Participants from a wide range of countries participated in the first truly international James Lind Priority Setting Partnership.
- Results may be less relevant to researchers within developing nations where education and awareness may be more important priorities.
- There was cross over between stakeholder groups so that many healthcare professionals and carers were also patients, therefore healthcare professionals may have been underrepresented.

INTRODUCTION

Hyperemesis gravidarum (HG) is severe vomiting and nausea in pregnancy. Affecting between 1 and 3% of all pregnancies (1), HG presents the major reason for hospital admissions in the first half of pregnancy. Yet, HG's pathophysiology has not been elucidated. Historically, HG has been an under-researched condition and the quality of HG research to date has been poor (2, 3).

A number of systematic reviews (2, 3) have shown that there is insufficient evidence to provide a definitive recommendation on the best way to treat HG. These reviews also concluded that the overall quality of evidence for HG treatments was low, and that further research was therefore required (3). The authors of the later review made several research recommendations in order of priority. However, neither of these systematic reviews engaged meaningfully with patients who had experienced hyperemesis gravidarum. As a result, the research recommendations have been criticised by patient advocacy groups for this condition (4, 5).

All too often questions being addressed by the research community are not aligned with the priorities of patients or healthcare professionals who seek answers to guide practice and decision making (6). In 2018, NHS England and the National Institute for Health Research (NIHR) conducted a needs assessment for medical research which highlighted that new

For Peer review only

For peer review only - http://bmjopen.bmj.com/site/about/guidelines.xhtml

research should only be undertaken where there is a defined evidence gap and a need which can be clearly articulated (7). The James Lind Alliance (JLA) is a non-profit initiative which seeks to address this mismatch by bringing patients, carers and clinicians together into Priority Setting Partnerships (PSP) to identify and prioritise the unanswered questions which they consider to be most important. Established in 2004, the JLA is funded by the NIHR and the PSPs to oversee the process with their established methodology and over 100 PSPs have been established since inception (8).

The HG PSP came together following the 2nd International Colloquium on Hyperemesis Gravidarum (ICHG) held in the United Kingdom in October 2017. During this event, leading HG researchers as well as patients from around the world agreed that setting Research Priorities for the condition was itself a priority and motivation for such a project was strong. It was noted that many of the topics relevant to patients are completely absent from the current literature (9), a mismatch noted by other JLA initiatives (6). Following this event, a subgroup of speakers and delegates with established international links to others in the field agreed to pursue the project and formed the initial PSP.

The HG PSP sought to identify the unanswered questions about HG treatment from patient and clinical perspectives and then prioritise those that patients and clinicians agree are the most important.

The objectives of the HG PSP were to:

- work internationally with patients and clinicians to identify uncertainties about the effects of HG treatments and management which have not yet been answered by existing research,
- determine by international consensus a prioritised list of those uncertainties, to guide future research, and
- to publicise the results of the PSP among researchers, research commissioning bodies and the general public in order to stimulate research in these areas.

METHODS

Patient and Public Involvement

People affected by HG, including patients, their carers and adult offspring were involved throughout this project from inception in line with the JLA methodology. The lead researcher, CD, is a patient representative and along with the patient representatives on the steering group ensured patient and carers were equal partners throughout the process including decision making around design, piloting surveys, participation and interpretation of results. The online HG patient community has been instrumental in disseminating research and will be utilised again to ensure broad public dissemination of the results from this project.

Steering group

The HG PSP steering group was established to oversee the project in accordance with the JLA guidelines (8). The steering group was chaired by a facilitator from JLA. Members consisted of both patients and clinicians from a range of professions involved in the care and treatment of HG, see supplementary file 1 for a list of members.

For peer review only - http://bmjopen.bmj.com/site/about/guidelines.xhtml

Due to the international nature of the project meetings were held online. The project ran from the first steering group meeting on the 21st March 2018 and cumulated with the final steering group meeting on 21st November 2019. The steering group's tasks were to provide input and consensus on the protocol and the survey, defining the scope of HG symptoms eligible, as well as the scope of the outcomes eligible for inclusion. Furthermore, the steering group monitored the progress of the project, provided input on the interpretation of participant feedback, and monitored the quality of the conclusions. All steering group decisions were reached by consensus.

Steering group meetings required the participation of at least three of each stakeholder groups to proceed.

Protocol, definitions, scope
The steering group developed and agreed on the study protocol (supplementary file 2 and available at: http://www.jla.nihr.ac.uk/priority-setting-partnerships/hyperemesis-gravidarum/) and formally adopted terms of reference, see figure 1. The steering group agreed that no formal definition of HG would be applied, such as hospital admission; people who considered themselves to have experienced HG (or their partners/offspring/carers) would be welcome to participate. Participation would not be limited by time since experience of the condition. However, questions specifically regarding mild nausea and vomiting of pregnancy or "morning sickness" would be considered out of scope. It was unanimously agreed that the only other criteria that would render questions to be out of scope were those regarding complementary and alternative therapies, such as homeopathy, acupuncture and herbal remedies including ginger.

Collection of research questions - Initial survey
Uncertainties were collected using an online survey (via SurveyMonkey®), which was also made available on paper. The survey was piloted by the steering group members.
Online dissemination of the survey followed an agreed social media strategy utilising the various partner organisation's platforms. Those steering group members who distributed paper copies within clinics accepted responsibility for collecting completed surveys and returning them to the lead researcher. Posters advertising the survey with a weblink and QR code were produced and distributed to relevant clinical setting where either steering group members or other engaged stakeholders were able to display them. Healthcare professional organisations from multiple countries were engaged with on social media platforms and directly to request assistance in promoting the survey to their audiences.

Participants could submit up to five questions regarding any aspect of HG research such as diagnosis, prognosis, treatment and the organisation of care. The survey platform could only be used once on any one device. The survey was translated into both Dutch and Norwegian and paper versions of both were made available online and pre-printed. Information provided at the start of the survey about how and why to take part was also provided in video format (Viewable at: https://vimeo.com/285975872). The relevant ethics waivers were obtained for each hospital setting and a Health Research Authority certificate of non-research activity provided where required (Supplementary File 3).

For peer review only - http://bmjopen.bmj.com/site/about/guidelines.xhtml

After the first two weeks of data collection, the demographics of respondents were reviewed by the steering group members, in order to allow advertising targeted at underrepresented stakeholder groups.

Participation in this stage of the project was not a prerequisite to participation in later stages such as the interim ranking and final workshop.

Processing the data

Once the initial survey was closed, the raw data was downloaded. Foreign language versions on either paper or online were translated independently by both a healthcare professional and a patient representative for each language.

Respondents were allocated unique numbers and their raw questions separated into unique entries with the corresponding demographic information retained with each question.

The lead researcher thematically coded all responses, including out of scope questions (See supplementary file 4 for codes). Queries were discussed within the steering group until consensus was reached. To ensure consistency of coding, the steering group checked the first 100 questions and, if this gave rise to significant disagreement, the number of questions to be checked could be expanded.

Indicative questions were formed which captured the meaning of the original questions in a logical and complete question. Each raw question was allocated to the indicative question which represented it. The steering group was divided into small groups to cross-check each raw question against the allocated indicative question to ensure that they were fully represented in lay language, and original meaning was not lost or skewed during the coding/question development process.

Evidence checking

In order to identify the responses for which enough evidence of sufficient quality was available to consider them as answered i.e. "unknown knowns", an extensive check of the evidence was undertaken according to an agreed protocol (supplementary file 5). Two reviewers, CD and HB, labelled the search results according to the question references related to and CD then cross checked each question against the evidence and the RCOG and ACOG guidelines for a suitably high level of evidence identified, i.e. systematic review with conclusive results.

The steering group convened to discuss the evidence for each question. We considered a question sufficiently answered if a systematic review and meta-analysis in the last 10 years had provided a summary statistic with a narrow confidence interval, which would make it unlikely to change with increased sample size. This was a deviation from the JLA recommendation that systematic review less than three years old are considered relevant and up to date (8), however the steering group agreed that this definition would be too narrow.

Interim Ranking Survey

For peer review only - http://bmjopen.bmj.com/site/about/guidelines.xhtml

To narrow the long list down for the final workshop, so that a manageable number of questions, of around 25, could be prioritised at the workshop, an interim online survey was conducted. Participants were presented with an online survey (via SurveyMonkey®) where they were asked to read each question individually and scored its importance on a scale of 1-10 where 1 was not at all important and 10 was the most important. Information was given at the start of the survey to encourage participants to use the full scale and to continue through the 65 questions. Several steering group members piloted the survey and comments were incorporated before launch. The questions appeared in a random order to each participant. The survey was only made available in English, however it was distributed internationally. Partial completions were not excluded.

The survey was initially open from 19th August to 18th September 2019 when a review of the demographic data was conducted. Following the review, the survey remained open until the 30th September 2019 in order to increase representation of healthcare professionals and a recruitment drive on social media and via healthcare professional organisations was implemented.

Consensus Workshop

Potential participants for the final consensus workshop were invited via email. Specifically, participants of the earlier surveys who had requested further information on the project were emailed as well as registered delegates for the International Colloquium on Hyperemesis Gravidarum (ICHG). Additionally, the workshop was advertised on social media through charity partner platforms and directly via email and word of mouth to steering group member contacts.

The workshop was facilitated by the JLA representative, the lead researcher (CD) was present as an observer. Participants were divided into three groups and across two sessions were switched around so that the balance of groups was always evenly distributed with an equal mix of patients and professionals. The participants were presented with all 26 indicative questions, in conjunction with the rank the indicative question had gained after the survey round. Healthcare, patient and carer ranks were separately presented to workshop participants. During the first two sessions the various groups ranked the questions 1-26 and the results were collated by the facilitators in order to reach an agreed consensus on the top 26 (8). The final session of the day saw the whole group convene, discuss any disputes regarding order, and make the final decision.

RESULTS

Uncertainty harvesting survey

The survey was open from 12th September – 16th November 2018, during which period two key healthcare professional conferences (General Practice and Early Pregnancy events) were attended and paper copies were distributed amongst attendees.

1009 participants took part in the survey, although not all participants completed all the demographic questions (between 774-964/1009, depending on the question). Eligibility was not determined by completeness of demographic questions and those who did not

For peer review only - http://bmjopen.bmj.com/site/about/guidelines.xhtml

complete demographic questions were not excluded. Of those who did answer any of the questions, 12.3% (n=118/963) were healthcare professionals and 97% (n=938/963) were patients and/or carers. Of the healthcare professionals who took part, 37% (n=44/118) had also suffered the condition themselves. Most participants identified as women (n=934). Only 30 people who identified as men took part of which 16 were healthcare professionals, 14 were carers (1 was both carer and professional), and one stated they had been diagnosed with hyperemesis gravidarum. The UK and the Netherlands had the most engagement however questions were submitted from across the globe, see figure 2.

Initial Survey results

After removing blank and non-answers (n=19), a total of 2899 raw questions were received and coded. Of these, 37 questions were considered out of scope, 33 related to complementary therapies and four were out of scope for other reasons; one suggested a class action law suit on behalf of HG patients, two were requests to take part in research and one was excluded because no member of the steering group understood the abbreviations in the submitted question.

Indicative questions

We developed 66 indicative questions, some of which had markedly more submissions than others, for example, “what is the cause?” (n=282), “which drugs are most effective?” (n=253) and “what is the chance it will reoccur in a subsequent pregnancy?” (n=212). As the indicative questions were developed, the types of participants (patient, healthcare professional, carer, offspring) who asked each question were recorded and can be viewed in supplementary file 6.

Evidence check

The search strategy revealed 627 references, which were labelled as to which question they related to by two reviewers. Each indicative question was then checked against the search results, the two identified guidelines (RCOG and ACOG) and the Cochrane Library, for whether it could be considered answered by systematic review.

The question “does the sex of the fetus effect the occurrence/severity of the condition” was removed following the evidence check as it was deemed to be sufficiently answered by a systematic review; there is a slight increased ratio of female to male fetus in HG pregnancies (OR 1.27; 95% CI 1.21–1.34, based on x=2,672,040) (10). Five questions were considered to have partial answers, such as the role of plasma or urine ketoanalysis in diagnosis and management of HG (11), none were considered to be sufficiently answered that no further research is required. In all 18 questions had no references related to them while one question, complications of HG, had 136 references the majority of which were case reports. The results of the evidence check with references per question is viewable in Supplementary file 6.

Interim ranking survey

The interim survey contained all 65 indicative questions. 1,115 participants took part in the ranking survey with 524 (47%) people ranking every question. Because the survey was displayed in a random order, all questions were ranked between 603-608 times each.

For peer review only - http://bmjopen.bmj.com/site/about/guidelines.xhtml

Healthcare professionals represented 9% of participants and were particularly hard to engage with despite efforts to inform them about the survey and encourage participation. Carers accounted for 3.6% of participants and there were notable differences in their priorities to those of patients and healthcare professionals, for example carers prioritised questions regarding symptom control for the sufferer over questions regarding harm to the fetus. International spread of participants was good with 32 countries represented.

The lack of healthcare professional representation was discussed with the steering group and a strategy to adjust for this was discussed with the JLA advisor. The question ranks were analysed and sorted by participant type. The first 21 questions were ranked above 25 by both patients and professionals and were automatically included. An additional 4, that healthcare professionals had ranked above these 21 where then included, to create a top 25. However, this resulted in one question which had previously been included by patient and healthcare professionals, and which was considered acutely important by the steering group without being sufficiently covered by other questions, to be eliminated. Therefore, 26 shortlisted questions went to the workshop for final ranking.

Consensus workshop

19 participants attended from 9 countries; The Netherlands, UK, USA, Australia, Germany, Sweden, Nigeria, Ireland, Italy. There were 10 healthcare professionals and 9 patient representatives. There was good consensus between the groups and, in the final session, the whole group came together and agreed the top ten until there was unanimous agreement. The top 26 questions are shown in order of priority in Table 1.

Table 1 - Top 26 ranked priorities for hyperemesis gravidarum research

Ranking	Question
1	Can we find a cure? What novel or new treatments are being developed/tested/used elsewhere which could have a curative effect and to address all the symptoms of HG rather than just the vomiting?
2	How can we most effectively manage HG? What clinical support measure is most important to people who have had hyperemesis and what did they find most beneficial? e.g. medical management, pharmaceutical review, nutrition support, rehydration, psychological support
3	What causes hyperemesis gravidarum?
4	Is HG preventable? What is the effect of preventative treatment or early intervention on the severity and duration of HG in a subsequent pregnancy?
5	What are the immediate and long-term effects of HG (including malnutrition and dehydration, stress) on the developing foetus (offspring)?
6	What are the immediate and long-term effects of the various medications/treatments on the developing foetus (offspring) throughout the various stages of pregnancy and in varying doses or combinations of treatments?
7	What are relative efficacies of the current medications and treatment options available? What is the optimal dose, route, timing and combination of the medications and what are the related side effects?
8	What are the immediate and long term, physical, mental and social consequences and complications of HG (including malnutrition and dehydration) on the pregnant person's body? (ie. Metabolic impact, DVT, depression, effects of dehydration)
9	What clinical measurements and markers are most useful in assessing, diagnosing, managing and monitoring hyperemesis?
10	What are the nutritional requirements of the 1st, 2nd and 3rd trimesters and how can people with HG achieve these goals? i.e. Oral supplements, fortifying food, dietary measures

For peer review only - http://bmjopen.bmj.com/site/about/guidelines.xhtml

11	How can symptoms of HG, other than vomiting, be effectively treated? For example, the nausea, excessive saliva, extreme sense of smell and fatigue.
12	Why are some cases of HG unresponsive to all antiemetics and how can we treat such cases?
13	What is the risk that HG will reoccur in a subsequent pregnancy? Does HG get progressively worse with subsequent pregnancies and what are the risk factors for reoccurrence?
14	Do clinical treatment guidelines for HG improve management and outcomes? And if so, how can guidelines be developed and implemented nationally (where none exist) and internationally for hospital and community settings? What should be included in guidelines?
15	How can people with a history of, or significant risk factors for HG be supported to plan for a pregnancy and does such planning improve outcomes? What should a pre-pregnancy plan contain?
16	How does HG impact on a person's (and their family's) quality of life? How does quality and efficacy of treatment impact that effect?
17	What is the currently level of knowledge about HG and its treatments among healthcare professionals (particularly GPs)? How can effective education for healthcare professionals be designed and delivered to improve the general knowledge and awareness of HG among HCPs?
18	What is the most effective IV rehydration regime; which solution in what quantity over what time period and how frequently? Does regular rehydration improve symptoms/outcomes/quality of life?
19	What is the effect of HG on mental health during (and after) pregnancy? What is the efficacy of psychotherapy on symptom management/pregnancy outcomes/quality of life? How can people access psycho-supportive services during pregnancy?
20	What are the barriers to taking/prescribing medication for HG? How can the risk and benefits of HG and its treatments be better communicated to support informed decision making and consent to treatment?
21	What are the barriers to accessing treatments/services and how can we reduce them to improve access?
22	What healthcare services exist and how can they collaborate and be organised to better identify, treat and support people with HG? For example, do services such as outpatient clinics or IV at home, improve outcomes and reduce the physical/mental burden of the condition?
23	How can the condition be effectively managed in the community to prevent lengthy hospital admissions?
24	What self-management and coping strategies and treatments do people with HG find most helpful?
25	What employment rights do people with HG have and what financial support is available to them?
26	Do specific specialist healthcare professional roles for conditions such as HG improve outcomes? How can such roles be developed for midwives/nurses/doctors?

One participant from Nigeria expressed concern that the top ten reflected the priorities for developed, high income nations and that in low-middle income countries the priorities would be different to those reflected here. It was agreed that, despite the international nature of the project, the report would reflect that these priorities are not representative of low-middle income country (LMIC) priorities and the exercise would need to be repeated with participants representative of those countries.

DISCUSSION

The top priorities for HG research, reached by stakeholder consensus, demonstrate the lack of knowledge about fundamental aspects of HG including effective treatment, and prognosis. Our results can be used by researchers to identify future research priorities that are most relevant to patients, carers and healthcare professionals, as well as being of use to

For peer review only

funders. The high numbers of patient responders to this PSP on HG, a transient, relatively rare condition limited to pregnancy, demonstrates the urgency felt among patients to prioritize progress in the identified research areas.

The theme of HG etiology and consequences were both highly prioritized: We don't yet have sufficient knowledge about the pathophysiology of HG or the effects of the condition on the developing fetus such as exposure to malnutrition, chronic dehydration, heightened anxiety, maternal isolation and trauma. Such information is vital for patients and clinicians to be able to make evidence based informed decisions about when and how to treat. The importance placed on identifying a cause and cure for the condition contrasts to many other JLA PSPs for conditions in which etiology and effective treatments have long been established. However, common to many previous PSPs, the HG priority top questions contain general uncertainties pertaining to prevention, best management and psychological impact on those affected.

While safety evidence regarding some medications is available and plentiful, particularly of the more commonly used medications such as doxylamine/pyridoxine, which is licensed for pregnancy sickness in various countries, and off-license treatments such as ondansetron (12, 13), questions remain over efficacy, combination therapy, dose, routes of administration and almost every other aspect of treating with these medications. The lack of robust information about the consequences of HG itself has led prescribers to assume that not treating is safer than treating (14-16). In recent years researchers have made a start identifying the risks of HG, including associations with autistic spectrum disorders among offspring (17), cardiometabolic disturbances (18) and poor birth outcomes (19). However, this is not sufficient to fully inform the important and difficult decisions required by patients and their healthcare providers during management of this condition.

While healthcare professional involvement was generally lower than patient involvement across JLA projects, healthcare professionals in this project only accounted for 12% and 9% of participants in the two survey rounds. This was disappointing given the efforts made to engage with healthcare professionals, the far-reaching connections from within the steering group and the presence of paper surveys in clinical settings in multiple countries. By comparison, the acne treatments PSP achieved 28% healthcare professional participation at the harvesting stage, and 64% at the ranking stage (20). The liver glycogen storage disease PSP achieved 35% at both stages (21). However, in comparison with other women's health and pregnancy conditions, engagement was similar. The miscarriage PSP had 1093 participants in round one, of which only 9.5% were healthcare professionals; the endometriosis PSP achieved 20% healthcare professional participation, of which 15% had experienced the condition themselves (22). Likewise, as in this project, the cross over between healthcare professionals and patients was notable in other women's health conditions, such as 11% in the preterm birth PSP (23) and 31% in the pessary use for prolapse PSP (24). The high level of patient engagement was anticipated by the authors as the patient population for this condition has previously demonstrated a strong interest for taking part in research with hundreds or even thousands of responses on surveys in a matter of days (25, 26). Although the low level of health care professional engagement was disappointing, it is not anticipated to negatively impact the goal of the PSP: to increase relevant research. Low healthcare professional engagement in pervious PSPs did not affect

For peer review only - http://bmjopen.bmj.com/site/about/guidelines.xhtml

research stimulation: both 'sight loss' and 'tinnitus' PSPs have reported impressive funding stimulation and research uptake in the years following their PSPs (27, 28) despite low healthcare professional engagement; 17% in the sight loss PSP (29) and 11-19% in the various stages of the tinnitus PSP (30). The lack of specialist healthcare professionals roles for certain conditions may account for some of the variation in engagement, for example, are no specific HG specialist nurse or midwife roles which may have affected engagement compared to other JLA projects such as diabetes, dermatology, and stroke which have nurse and physician specialists. Alternatively, the low healthcare professional engagement in the HG PSP may reflect a more general lack of interest in, or knowledge of HG among healthcare disciplines involved in HG care.

This PSP was the second fully international PSP so far and representation was obtained from a large number of countries. However, high income countries were significantly over-represented and, despite efforts to engage doctors and patients from LMICs via social media and personal contacts, there was little engagement in the survey stages and only one representative at the final workshop. By comparison, the only other international PSP for liver glycogen storage disease had three representatives (27%) from LMIC (exclusively South American countries) at its workshop (21). This is likely due to lack of funding although, for this project, travel bursaries were available including full bursaries for participants from LMICs. The JLA does not currently have a protocol for establishing how representative an international PSP should be or indeed how internationally relevant Priority Lists can be adopted in other countries. This can lead to significant research effort waste. For example, a PSP established a top ten priority list for gestational diabetes in Canada in 2017 using JLA methodology (31), yet a UK specific group is conducting a JLA for diabetes in pregnancy this year (32). Within our workshop, it was noted that the top ten priorities may not be relevant for LMICs. While repeating this process in similar high-income countries would be considered wasteful, repeating the prioritisation process of the identified uncertainties in LMICs may be a worthwhile project.

CONCLUSION

Following the JLA method, the international research arena can now direct resources to the top ten priority research questions, which will have the greatest impact for people affected by HG and the healthcare professionals caring for them.

Funding:
Funding was received from Pregnancy Sickness Support for the James Lind Alliance facilitator.
The final workshop was funded by the Bikkja Trust who also provided some bursaries for attendance at the workshop.

Author Contributions:
CD and RCP were the lead researchers, instigating the application to the JLA. CD organised the steering group meetings, designing and building the surveys, analysed the data, conducted the evidence check and wrote the majority of the final manuscript under supervision of RCP. PE (JLA Chair) chaired all the meetings, lead the consensus workshop and ensured compliance with methodology throughout. KL took minutes for steering group

For peer review only - http://bmjopen.bmj.com/site/about/guidelines.xhtml

meetings, built and distributed surveys, organised the consensus workshop and acted as a facilitator in the workshop. RS conducted the searches for the evidence check and HB screened the results with CD. Members of the steering committee RC, BC, RG, NG, KMG, MM, DM, CN-P, MOH, HP, KS, JT and EW all attended a majority of the meetings, agreed the initial protocol and the evidence check protocol, piloted and signed off the surveys and disseminated them, checked the raw questions against the indicative ones, reviewed the evidence check results and agreed the final longlist. CD, RCP, KL, PE, RG, NG, KMG, MM and MOH were present at the consensus workshop. All authors reviewed and contributed to the final manuscript and approved it prior to submission.

Competing interests:
CD is chair of the charity Pregnancy Sickness Support, which funded the JLA aspect of this project. RG and MOH are trustees, KL is an employee and EW is a volunteer for Pregnancy Sickness Support. CD and CN-P are trustees of the Bikkja Trust which funded the consensus workshop and provided some travel bursaries. CN-P has received consultancy and speaker fees from Alliance Pharma.

Data sharing statement:
All survey data and submitted questions will be available via the James Lind Alliance website without access restrictions (http://www.jla.nihr.ac.uk/priority-setting-partnerships/hyperemesis-gravidarum/)

REFERENCES

1. Einarson TR, Piwko C, Koren G. Quantifying the global rates of nausea and vomiting of pregnancy: a meta analysis. *J Popul Ther Clin Pharmacol* 2013;20(2):e171-83.
2. Boelig RC, Barton SJ, Saccone G, *et al*. Interventions for treating hyperemesis gravidarum. *Cochrane Database Syst Rev* 2016 May 11;(5): DOI: 10.1002/14651858.CD010607.pub2.
3. O'Donnell A, McParlin C, Robson SC, *et al*. Treatments for hyperemesis gravidarum and nausea and vomiting in pregnancy: a systematic review and economic assessment. *Health Technol Assess* 2016;20(74):1-268.
4. Chapman S. Hyperemesis gravidarum: sufferers need support, compassion and better research. *Evidently Cochrane* 2016. http://www.evidentlycochrane.net/hyperemesis-gravidarum-sufferers-need-support-compassion-and-better-research/ Assessed May 2020
5. Dean C, Goddard S. Patient and Public Involvement in Designing an Online Survey about Hyperemesis Gravidarum. *MIDIRS Midwifery Digest* 2016;26(3):283-90.
6. Crowe S, Fenton M, Hall M, *et al*. Patients', clinicians' and the research communities' priorities for treatment research: there is an important mismatch. *Res Involv Engagem*. 2015;1(2). https://doi.org/10.1186/s40900-015-0003-x
7. NHS England, National Institute for Health Research. NHS England's Research Needs Assessment 2018. Southampton: NHS England; 2018. p. 19.
8. The James Lind Alliance. The James Lind Alliance Guidebook Version 6. National Institute for Health Research; 2016.
9. Painter RC. Need for Research Priorities in Hyperemesis Gravidarum. International Conference on Hyperemesis Gravidarum; 2017; Windsor, UK.

For peer review only - http://bmjopen.bmj.com/site/about/guidelines.xhtml

10. Veenendaal MV, van Abeelen AF, Painter RC, *et al*. Consequences of hyperemesis gravidarum for offspring: a systematic review and meta-analysis. *Bjog*. 2011;118(11):1302-13.
11. Niemeijer MN, Grooten IJ, Vos N, *et al*. Diagnostic markers for hyperemesis gravidarum: a systematic review and metaanalysis. *Am J Obstet Gynecol*. 2012;211(2):150.e1-.e15.
12. Kaplan YC, Richardson JL, Keskin-Arslan E, *et al*. Use of ondansetron during pregnancy and the risk of major congenital malformations: A systematic review and meta-analysis. *Reprod Toxicol*. 2019;86:1-13.
13. Huybrechts KF, Hernández-Díaz S, Straub L, *et al*. Association of Maternal First-Trimester Ondansetron Use With Cardiac Malformations and Oral Clefts in Offspring. *JAMA*. 2018;320(23):2429-37.
14. Dean C, Murphy C. I could not survive another day: Improving treatment and tackling stigma: lessons from women's experiences of abortion for severe pregnancy sickness. London: Pregnancy Sickness Support and BPAS; 2015.
15. Dean C. Does the historical stigma of hyperemesis gravidarum impact healthcare professional's attitudes and treatment towards women with the condition today? A review of recent literature. *MIDIRS Midwifery Digest*. 2016;26(2):186-94.
16. Gadsby R, Barnie-Adshead T, Sykes C. Why won't doctors prescribe antiemetics in pregnancy? *BMJ*. 2011;343:d4387.
17. Getahun D, Fassett MJ, Jacobsen SJ, *et al*. Autism Spectrum Disorders in Children Exposed in Utero to Hyperemesis Gravidarum. *Am J Perinatol.* 2019.
18. Ayyavoo A, Derraik JGB, Hofman PL, *et al*. Hyperemesis gravidarum and long-term health of the offspring. *Am J Obstet Gynecol*.210(6):521-5.
19. Fiaschi L, Nelson-Piercy C, Gibson J, *et al*. Adverse Maternal and Birth Outcomes in Women Admitted to Hospital for Hyperemesis Gravidarum: a Population-Based Cohort Study. *Paediatr Perinat Epidemiol*. 2018;32(1):40-51.
20. Layton A, Eady EA, Peat M, *et al*. Identifying acne treatment uncertainties via a James Lind Alliance Priority Setting Partnership. *BMJ Open*. 2015;5(7):e008085.
21. Peeks F, Boonstra WF, de Baere L, *et al*. Research priorities for liver glycogen storage disease: An international priority setting partnership with the James Lind Alliance. *J Inherit Metab Dis*. 2019.
22. Horne AW, Saunders PTK, Abokhrais IM, *et al*. Top ten endometriosis research priorities in the UK and Ireland. *The Lancet*. 2017;389(10085):2191-2.
23. Oliver S, Uhm S, Duley L, *et al*. Top research priorities for preterm birth: results of a prioritisation partnership between people affected by preterm birth and healthcare professionals. *BMC Pregnancy Childbirth*. 2019;19(1):528.
24. Lough K, Hagen S, McClurg D, *et al*.. Shared research priorities for pessary use in women with prolapse: results from a James Lind Alliance Priority Setting Partnership. *BMJ Open*. 2018;8(4):e021276.
25. Dean CR, O'Hara ME. Ginger is ineffective for hyperemesis gravidarum, and causes harm: an internet based survey of sufferers. *MIDIRS Midwifery Digest*. 2015;25(4):449-55.
26. Nana M, Dean C, Bevan G, *et al*. Severe hyperemesis gravidarum increases rates of termination of pregnancy and suicidal ideation: results from a UK questionnaire completed by >5000 participants. International Colloquium Hyperemesis Gravidarum; 2019 10th October 2019; Amsterdam, Netherlands: ICHG.

For peer review only - http://bmjopen.bmj.com/site/about/guidelines.xhtml

27. Fight for Sight. Report on the funding recieved for top priority research following the James Lind Alliance PSP 2019 Available from: https://www.fightforsight.org.uk/our-research/sight-loss-priority-setting-partnership/funded-research/. Accessed May 2020
28. British Tinnitus Association. Five Years Later: What happened after the James Lind Alliance Tinnitus Priority Setting Partnership. Sheffield: British Tinnitus Association; 2018.
29. Rowe F, Wormald R, Cable R, *et al.* The Sight Loss and Vision Priority Setting Partnership (SLV-PSP): overview and results of the research prioritisation survey process. *BMJ Open*. 2014;4(7):e004905.
30. Hall DA, Mohamad N, Firkins L, *et al*. Identifying and prioritizing unmet research questionsfor people with tinnitus: the James Lind Alliance Tinnitus Priority Setting Partnership. *Clinical Investigation*. 2013;3(1):21-8.
31. Rees SE, Chadha R, Donovan LE, et al. Engaging Patients and Clinicians in Establishing Research Priorities for Gestational Diabetes Mellitus. 2017;41(2):2352-3840.
32. Diabetes and Pregnancy Priority Setting Partnership. Protocol Version 1.0. 2019. https://www.npeu.ox.ac.uk/downloads/files/jla-psp/FINAL1.0_JLA-PSP-Diabetes-and-Pregnancy-Protocol.pdf Accessed May 2020

Figure Captions:

Figure 1 - the JLA process with steps completed to date

Figure 2 - Country of residence of participants in the initial survey

For peer review only

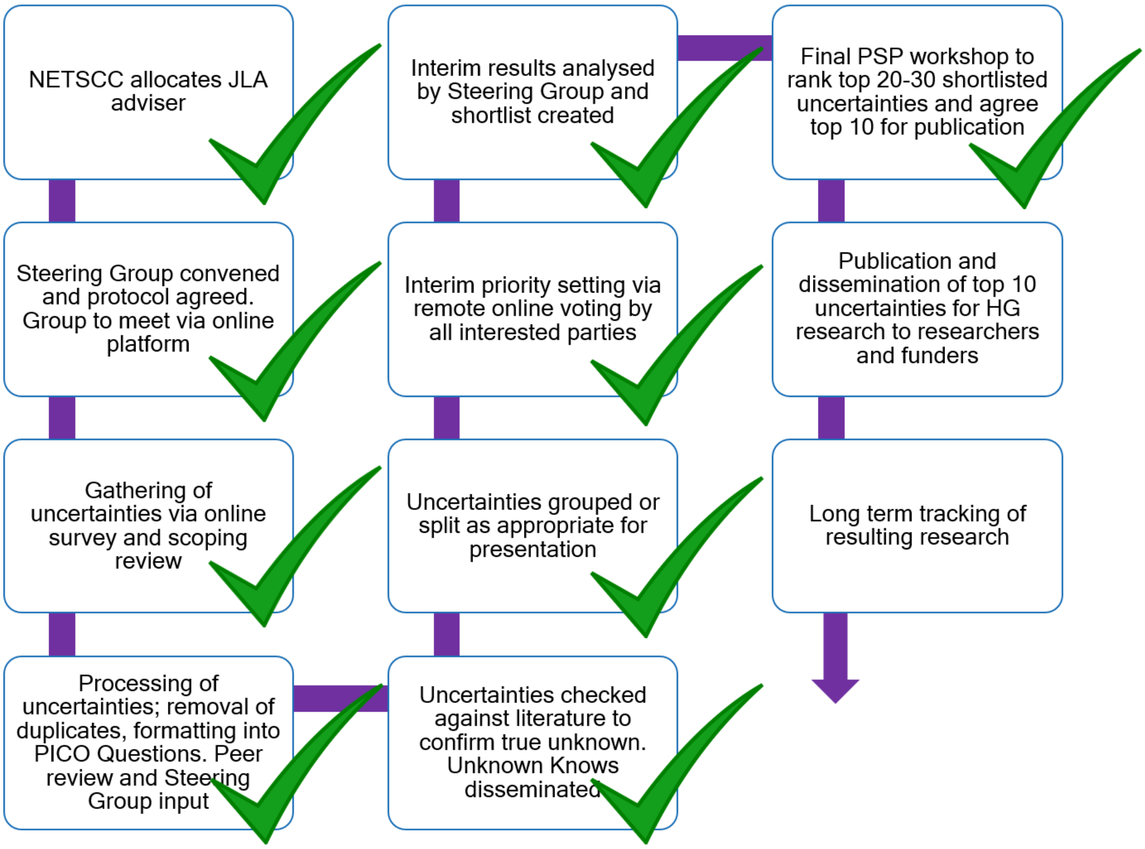

Figure 1 - the JLA process with steps completed to date

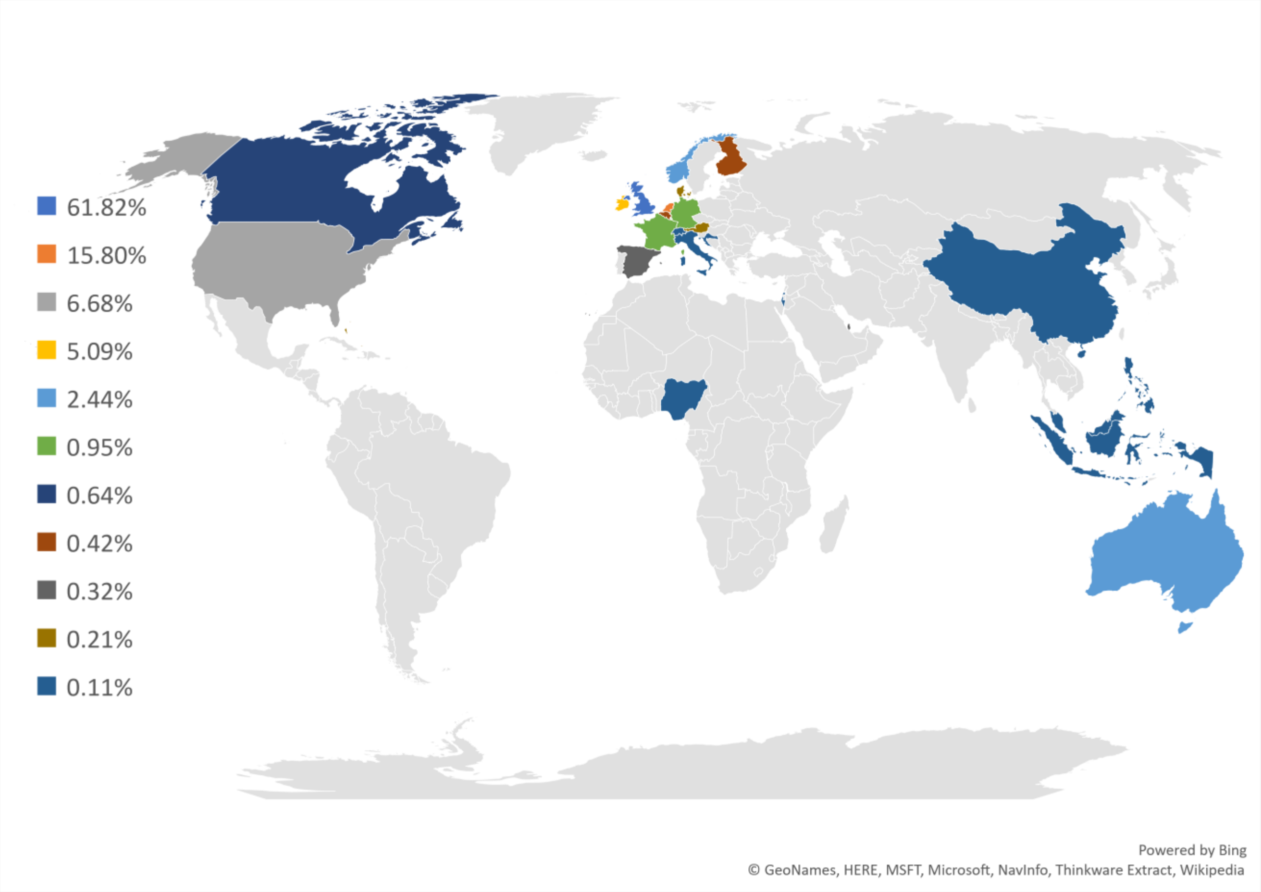

Figure 2 - Country of residence of participants in the initial survey

Supplementary File 1:

Members of the HG PSP Steering Committee

**Patient Representatives**	Margaret O'Hara (MOH)	Pregnancy Sickness Support (PSS) (United Kingdom(UK) charity)
	Emma Watford (EW)	Pregnancy Sickness Support (PSS) (United Kingdom(UK) charity)
	Norah Gauw (NGT)	ZEHG, The Netherlands
	Karina Fee (KF)	Hyperemesis Ireland
	Kimber MacGibbon (KMG)	Hyperemesis Education and Research (HER) Foundation (United States (US) and international charity)
**Clinical representatives**	Dr Rebecca Painter (RP) (Ob/Gyne)	Amsterdam University Medical Centres (and ZEHG Charity)
	Prof Catherine Nelson-Piercy (C N-P) (Obstetric Physician)	Professor of obstetric medicine and consultant obstetric physician, Guy's and St Thomas' Hospital
	Prof Brian Cleary (BC)(Pharmacist)	Rotunda Hospital, Dublin (and Hyperemesis Ireland Charity)
	Marian MacBride (MM) (Senior Dietician)	Health Service Executive, Dublin (and Hyperemesis Ireland Charity)
	Katherine Shorter (KS) (Nurse, HG Day Unit)	Nottingham University Hospital Trust
	Deirdre Munro (DM) (Midwife)	Global Village of Midwives Network, Portiuncula University Hospital, Co. Galway Ireland (and Hyperemesis Ireland Charity)
	Dr Roger Gadsby (RG) (General Practitioner)	Royal College of General Practitioners + experience of running a JLA PSP and PSS UK Charity
	Prof Jone Trovik (JT) (Ob/Gyne)	Haukeland University Hospital, Bergen, Norway
	Dr Ria Clarke (RC) (Ob/Gyne)	Junior Doctor, UK
	Dr Helen Penny (HP) (Clinical Psychologist)	Cardiff University
**The James Lind Alliance (JLA)**	Patricia Ellis (PE)	James Lind Alliance

For peer review only

A JAMES LIND ALLIANCE PROJECT

HGPSP
PRIORITY SETTING PARTNERSHIP

James Lind Alliance
Priority Setting Partnerships

HYPEREMESIS GRAVIDARUM PRIORITY SETTING PARTNERSHIP - PROTOCOL

For peer review only

PROTOCOL DRAFT 27/02/2018

PURPOSE OF THE PSP AND BACKGROUND

The purpose of this protocol is to set out the aims, objectives and commitments of the Hyperemesis Gravidarum (HG) Priority Setting Partnership (PSP) (HG PSP) and the basic roles and responsibilities of the partners therein. It is recommended that the Protocol is reviewed by the Steering Group and updated on at least a quarterly basis.

The James Lind Alliance (JLA) is a non-profit initiative, established in 2004. It brings patients, carers and clinicians together in Priority Setting Partnerships (PSPs). These partnerships identify and prioritise uncertainties, or 'unanswered questions', about the effects of treatments or other aspects of the condition, such as diagnosis, prognosis and the organisation of care, that they agree are the most important. The aim of this is to help ensure that those who fund health research are aware of what really matters to both patients and clinicians. The National Institute for Health Research (NIHR – www.nihr.ac.uk) funds the infrastructure of the JLA to oversee the processes for priority setting partnerships, based at the NIHR Evaluation, Trials and Studies Coordinating Centre (NETSCC), University of Southampton.

The HG PSP has come together following the 2nd International Colloquium on Hyperemesis Gravidarum held in the UK in October 2017. During this event leading HG researchers as well as patients from around the world agreed that setting Research Priorities for the condition was itself a priority and motivation for such a project was strong. Following this event, a subgroup of speakers and delegates with established international links to others in the field agreed to pursue the project and formed the initial PSP.

Historically, HG has been an under-researched condition and the quality of HG research to date has been poor (Boelig et al. 2016; McParlin et al. 2016). In part efforts have been hampered by a lack of international definition of HG and core outcome set for research, thus rendering much of the data too heterogenetic for meta-analysis

(Grooten et al. 2015). Thankfully these barriers are being tackled and an internationally agreed definition and core outcome set are anticipated in 2018 (ref conference).

A Cochrane review in 2016 (Boelig et al. 2016) concluded that there was insufficient evidence to provide a definitive recommendation on the best way to treat severe nausea and vomiting in early pregnancy. A simultaneous systematic review concluded that the overall quality of evidence for HG treatments was low and that further research was therefore required (O'Donnell et al. 2016). In particular the authors of the review made a number of research recommendations in order of priority. However, neither of these systematic reviews engaged meaningfully with patients who have experienced hyperemesis gravidarum and as a result the research recommendations have been criticised by patient advocacy groups for this condition (Chapman 2016) (email communication with HTA and public engagement exercises).

An increase in public and professional interest in HG in recent years and recognition of the long-term consequences for offspring has led to increase in funding and a flurry of research proposals. There is also a strong motivation among women affected by HG to take part in research and in patient and public involvement in the development of HG research (Dean and Goddard 2016).

AIMS AND OBJECTIVES OF THE HG PSP

The aim of the HG PSP is to identify the unanswered questions about HG treatment from patient and clinical perspectives and then prioritise those that patients and clinicians agree are the most important.

The objectives of the HG PSP are to:

- Together with patients and clinicians, work internationally to identify uncertainties about the effects of HG treatments and management which have not yet been answered by existing research
- Determine by international consensus a prioritised list of those uncertainties, to guide future research
- To publicise the results of the PSP among researchers, research commissioning bodies and the general public in order to stimulate research in these areas

THE STEERING GROUP

The HG PSP will be led and managed Caitlin Dean (CD) with the following steering group:

**Patient Representatives**	Margaret O'Hara (MOH)<br>Emma Watford (EW)	Pregnancy Sickness Support (PSS) (United Kingdom (UK) charity)

For peer review only - http://bmjopen.bmj.com/site/about/guidelines.xhtml

	Norah Gauw (NG)	The Netherlands
	Karina Fee (KF)	Hyperemesis Ireland
	Kimber MacGibbon (KMG)	Hyperemesis Education and Research (HER) Foundation (United States (US) and international charity)
**Clinical representatives**	Dr Rebecca Painter (RP)	Amsterdam Medical Centre
	Prof Catherine Nelson-Piercy (C N-P) (Obstetric Physician)	Professor of obstetric medicine and consultant obstetric physician, Guy's and St Thomas' Hospital
	Dr Brian Cleary (BC)(Pharmacist)	Rotunda Hospital, Dublin
	Marian MacBride (MM) (Dietician)	Rotunda Hospital, Dublin
	Kate Shorter (KS) (Nurse, HG Day Unit)	Nottingham University Hospital Trust
	Deirdre Munro (DM) (Midwife)	Global Village of Midwives Network
	Dr Roger Gadsby (RG) (General Practitioner)	Royal College of General Practitioners + experience of running a JLA PSP
	Jone Trovik (JT) (Ob/Gyne)	Haukeland University Hospital, Bergen, Norway
	Ria Clarke (RC) (Ob/Gyne)	Junior Doctor, UK
	Helen Penny (HP) (Psychologist)	Cardiff University
**The James Lind Alliance (JLA)**	Patricia Ellis (PE)	James Lind Alliance

For peer review only - http://bmjopen.bmj.com/site/about/guidelines.xhtml

The Steering Group includes representation of patient/carer groups and clinicians[1].

The Steering Group will agree the resources, including time and expertise that they will be able to contribute to each stage of the process. The JLA will advise on this.

THE WIDER PARTNERS

Organisations and individuals will be invited to be involved with the PSP as partners. Partners are groups or individuals who will commit to supporting the PSP by disseminating the PSP survey and helping the PSP to gather questions and uncertainties of practical clinical importance relating to the treatment and management of the health problem in question. Partners represent the following groups:

- people who have had HG
- carers and offspring of people who have had HG
- medical doctors, nurses and professionals allied to medicine with clinical experience of HG.

It is important that all organisations which can reach and advocate for these groups should be invited to become involved in the PSP. The JLA Adviser will take responsibility for ensuring the various stakeholder groups are able to contribute equally to the process.

EXCLUSION CRITERIA

Some organisations may be judged by the JLA or the Steering Group to have conflicts of interest, which may be perceived to adversely affect those organisations' views, causing unacceptable bias, for example, pharmaceutical companies or individual stakeholders in such companies. As such conflicts of interest would be likely to affect the ultimate findings of the PSP, those organisations will not be invited to participate. It is possible, however, that interested parties may participate in a purely observational capacity when the Steering Group considers it may be helpful.

[1] In some cases, it has been suggested that researchers are represented at this level, to advise on the shaping of research questions. However, researchers cannot participate in the prioritisation exercise. This is to ensure that the final prioritised research questions are those agreed by patients, carers and clinicians only, in line with the JLA's mission.

For peer review only - http://bmjopen.bmj.com/site/about/guidelines.xhtml

THE METHODS THE PSP WILL USE

The methods adopted in any stage will be agreed through consultation between the Steering Group members, guided by the PSP's aims and objectives.

STEP 1: IDENTIFICATION AND INVITATION OF POTENTIAL PARTNERS

Potential partner organisations will be identified through a process of peer knowledge and consultation, through the Steering Group members' networks. Potential partners will be contacted and informed of the establishment and aims of the HG PSP and may be invited to participate in an online discussion.

People wishing to participate will be able to register using an online form with a convenient email address for future communication via mailchimp. Details of which stakeholder group they identify as and which organisation they represent will be gathered.

Initial organisations will include:

Patient Charities:	Pregnancy Sickness Support
	HER Foundation
	Hyperemesis Ireland
	ZEHG (Netherlands organisation)
	Hyperemesis Gravidarum Facebook Group (10,000 + members)
	Hyperemesis Gravidarum Norway
	NB. A number of countries have budding organisations and informal patient networks, such as Australia and Germany. Representatives from each country we are aware of will be contacted. Additionally, the HER Foundation has at least one contact in most countries worldwide.
Clinician Networks:	Royal College of Obstetricians and Gynaecologists
	Royal College of General Practitioners
	Royal College of Midwives
	Royal College of Nursing Midwifery Forum and Women's Health Forum
	Association of Early Pregnancy Units
	American College of Obstetricians and Gynaecologists
	Dutch NVOG

For peer review only - http://bmjopen.bmj.com/site/about/guidelines.xhtml

	Dutch O&G Nurses
	"RCOG" and "RCM" equivalents for as many countries as possible will be sought out and approached.

STEP 2: INITIAL STAKEHOLDER INTRODUCTION

The initial stakeholder information sheet for those registering interest in participation will have several key objectives:

- To welcome and introduce potential members of the HG PSP
- To present the proposed plan for the PSP
- To initiate discussion, answer questions and address concerns
- To invite potential partner organisations which will commit to the PSP to identify themselves and identify individuals who will be those organisations' representatives and the PSP's principal contacts
- To establish principles upon which an open, inclusive and transparent mechanism can be based for contributing to, reporting and recording the work and progress of the PSP.

This will be done via email using the emails previously registered. A forum within the project website can be used to initiate discussion, answer questions and address concerns.

STEP 3: IDENTIFYING TREATMENT UNCERTAINTIES

Each partner will disseminate an online survey to its members to solicit questions and uncertainties of practical clinical importance relating to the treatment and management of HG. A period of 8 weeks will be given to complete this exercise. Online surveys have been found to be an effective way of engaging with women affected by HG (Dean and Goddard 2016) and for collecting research priorities (Gill et al. 2013).

Predominately treatment uncertainties will be collected using an online questionnaire however the methods may be designed according to the nature and membership of each organisation, but must be as transparent, inclusive and representative as practicable. Methods may include membership meetings, email consultation, postal questionnaires, internet message boards and focus group work.

Existing sources of information about treatment uncertainties for patients and clinicians will be searched. These can include question-answering services for patients and carers and for clinicians; research recommendations in systematic reviews and clinical guidelines; protocols for systematic reviews being prepared and registers of ongoing research. A separate protocol for this search will be written.

STEP 4: REFINING QUESTIONS AND UNCERTAINTIES

For peer review only - http://bmjopen.bmj.com/site/about/guidelines.xhtml

The consultation process will produce "raw" unanswered questions about diagnosis and the effects of treatments. These raw questions will be assembled, categorised and refined by CD and RP into "collated indicative questions" which are clear, addressable by research and understandable to all. Similar or duplicate questions will be combined where appropriate and the questions prepared for prioritisation. The following stages outlined by the JLA PSP Data Management Guide (James Lind Alliance 2016) will be followed:

Stage 1: Downloading the survey data

Survey responses from survey monkey will be downloaded and managed in an Excel spreadsheet. Additional uncertainties identified through other methods, ie. Literature search, discussion groups etc. will be added into the spreadsheet with their source documented. Each respondent and their demographic data will be identified with a unique identifier which will be attached to each of their submissions where one person submitted multiple responses. Personal identifying details will then be removed to anonymise the data.

Stage 2: Remove out of scope submissions

The scope of this PSP will need to be determined by the steering group in advance *(see minutes of steering group meeting 2 – scope). Submissions which fall outside of the scope or are established to not be uncertainties will be removed and kept separately with associated detail such as number of each stakeholder group that submitted the question and their demographics. These exclusions will be reviewed by the steering group to confirm the exclusion and decisions regarding what to do with them will be made (ie. Passing to other organisations or feeding back to the participants)

Stage 3: Categorising eligible submissions

Submissions will be categorised using the existing taxonomy by the UK Clinical Research Collaboration: Health Research Classification System (UK Clinical Research Collaboration 2009).

Stage 4: Format the submissions

Submissions will be rephrased to clarify the precise uncertainty. Where possible the "Patient/Population, Intervention, Comparison, Outcome" (PICO) format will be used. Where this is not possible uncertainties will be formatted in a way that will be useful and valuable to the research community. Lay language will be used.

Submissions which cannot be standardised will be listed and referred to the steering group for discussion. Where possible they may be combined with other eligible submissions. Formatted questions will be recorded with their frequency of submission by each stakeholder group (ie. How many times it was submitted by patients and clinicians).

Stage 5: Verifying the uncertainties

For peer review only - http://bmjopen.bmj.com/site/about/guidelines.xhtml

Systematic reviews and guidelines will be identified and checked by CD to see to what extent these refined questions have, or have not, been answered by previous research, specifically, up-to-date systematic review or meta-analysis. Sometimes, uncertainties are expressed that can in fact be resolved with reference to existing research evidence - ie they are "unrecognised knowns" and not uncertainties. These 'answerable questions' will be recorded and dealt with separately from the 'true uncertainties' considered during the research priority setting process. If a question about treatment effects can be answered with existing information but this is not known, it suggests that information is not being communicated effectively to those who need it.

Uncertainties which are not adequately addressed by previous research will be collated and recorded on a template supplied by the JLA by CD. The list will be reviewed and agreed by the steering group.

Evidence checking protocol as agreed by Steering Group on 15th July 2019

HYPEREMESIS GRAVIDARUM PRIORITY SETTING PARTNERSHIP

EVIDENCE CHECKING PROTOCOL

This summary sets out the approach to the evidence checking process for the Hyperemesis Gravidarum Priority Setting Partnership (HG PSP), including the specific role of the Steering Group (SG) and what will be done with the findings.

THE PURPOSE OF EVIDENCE CHECKING

Systematic reviews and guidelines will be identified and checked by Caitlin Dean (CD) to see to what extent the refined questions, generated from the raw survey data, have, or have not, been answered by previous research. Sometimes, uncertainties are expressed that can in fact be resolved with reference to existing research evidence - ie they are "unrecognised knowns" and not uncertainties. If a question about treatment effects can be answered with existing information but this is not known, it suggests that information is not being communicated effectively to those who need it. Whereas uncertainties not adequately addressed by previous research will go forward to the next stage for prioritisation.

THE PROCESS FOR EVIDENCE CHECKING

Searches

For peer review only - http://bmjopen.bmj.com/site/about/guidelines.xhtml

A scoping search of Embase and Medline was conducted in August 2018 by information specialist Rene Spijker as part of a wider evidence mapping project running concurrently with the HG PSP. The search strategy sought to find all papers about HG without limitations. Embase, Medline were search for the key terms hyperemesis gravidarum/ or (("Excessive vomiting" or (pernicious adj3 vomiting) or hyperemesis) and (gravid* or pregn* or gestation or antenatal)). Additionally, for this evidence check, searches will cover the Cochrane Database of Systematic Reviews, the Cochrane Controlled Trials Register and trip Database for Guidelines. The NICE, RCOG and ACOG guidelines will be checked for references with a suitably high level of evidence (ie. Systematic review within 10 years).

The results will been screened and labelled as to which question number they relate to by two researchers independently.

Each question will be checked against the labelled results for systematic reviews with conclusive results which directly answer the question.

Additionally, the Cochrane Library and the RCOG and ACOG guidelines will be checked for each question for a suitably high level of evidence with a definitive recommendation/answer.

Excluding NICE

NICE guidelines will not be used for evidence checking for two reasons;

1. Statements and recommendations are not referenced in a way that can be cross checked for level of evidence.
2. The website states: *"This CKS topic is largely based on the Royal College of Obstetricians and Gynaecologists guideline: The management of nausea and vomiting of pregnancy and hyperemesis gravidarum [RCOG, 2016], the American College of Obstetricians and Gynecologists Practice Bulletin: Nausea and vomiting of pregnancy [ACOG, 2015], and review articles."* These references are already being check as stated above.

10 year inclusion

Due to the paucity of HG research and lack of funding the SG felt that a three-year limit on SRs, as recommended by the James Lind Alliance, was too limited. Therefore, SRs within the last 10 years will be deemed acceptable.

Final outcome

For peer review only - http://bmjopen.bmj.com/site/about/guidelines.xhtml

Uncertainties which are not adequately addressed by previous research will be collated and recorded on a template supplied by the JLA (see Appendix A) by CD and will go onto the next stage for prioritisation.

UNKNOWN KNOWNS

Questions which are identified as already answered, but which people are unaware are already answered, ie. Unknown knowns, need to be translated into accessible information for the general public. CD will write a general article outlining the questions we have answers to and the answers themselves (not scientific) in lay language for dissemination to the general public via the main international charities, blogs and newsletters. Additionally, authors of guidelines such as the RCOG and NICE will be informed of the findings to ensure they are included in updates to the guidelines (if they are not already).

FURTHER INFORMATION

The JLA Guidebook provides step-by-step guidance to establishing and managing PSPs. You can find the Guidebook at www.jla.nihr.ac.uk

STEP 5: PRIORITISATION – INTERIM AND FINAL STAGES

The aim of the final stage of the priority setting process is to prioritise through consensus the identified uncertainties relating to the treatment or management of HG. This will be carried out by members of the Steering Group and the wider partnership that represents patients and clinicians. The stages will depend on the number of true uncertainties identified.

If there are more than 100 the Steering group will need to do an initial prioritisation to reduce the list to a more manageable number (max 80). Criteria for consideration include:

- Was the question suggested by both patients and clinicians?
- How many people suggested the question from each stakeholder group and in total?
- Was the question submitted by different kinds of clinicians or internationally?
- Whether the question overlaps with other ones or research recommendations

Interim Priority Setting

This will be conducted as an online survey. Questions will be scored and ranked by participants and the process tracked with relevant details. From here approx. 25 priorities will be retained for the final priority setting meeting.

For peer review only - http://bmjopen.bmj.com/site/about/guidelines.xhtml

Final Priority setting

The final stage to identify 10 prioritised uncertainties will take place at the ICHG Conference in Amsterdam in October 2019. The JLA will facilitate this process and ensure transparency, accountability and fairness. Participants will be expected to declare their interests in advance of this meeting.

DISSEMINATION OF FINDINGS AND RESEARCH

FINDINGS AND RESEARCH

The data will be submitted to the JLA for publication on its website on completion of the priority setting exercise, taking into account any changes made at the final workshop, in order to ensure that PSP results are publicly available.

It is anticipated that the findings of the HG PSP will be reported to funding and research agenda setting organisations such as the NIHR and the major research funding charities. Steering Group members and partners are expected to develop the prioritised uncertainties into research questions, and to work to establish the research needs of those unanswered questions to use when approaching potential funders, or when allocating funding for research themselves, if applicable. It is anticipated that Pregnancy Sickness Support will aim to provide modest grants for researchers addressing priority questions.

PUBLICITY

In addition to an academic paper a simple language report will be produced by the partner organisations and a PR strategy implemented.

A review of research resulting from the priority list will be presented at ICHG 2021 in Dublin and priority given at that conference to presentations and posters addressing the priority questions.

For peer review only

REFERENCES

Boelig RC, Barton SJ, Saccone G, Kelly AJ, Edwards SJ, Berghella V. 2016. Interventions for treating hyperemesis gravidarum. Cochrane Database Syst Rev 5:DOI: 10.1002/14651858.CD010607.pub2.

Chapman S. 2016. Hyperemesis gravidarum: sufferers need support, compassion and better research. Evidently Cochrane.

For peer review only - http://bmjopen.bmj.com/site/about/guidelines.xhtml

Dean C, Goddard S. 2016. Patient and Public Involvement in Designing an Online Survey about Hyperemesis Gravidarum. MIDIRS Midwifery Digest 26(3):283-290.

Gill FJ, Leslie GD, Grech C, Latour JM. 2013. Using a web-based survey tool to undertake a Delphi study: application for nurse education research. Nurse Educ Today 33(11):1322-8.

Grooten I, Roseboom T, Painter R. 2015. Barriers and Challenges in Hyperemesis Gravidarum Research. Nutr Metab Insights 8(Suppl 1):33-9.

James Lind Alliance. 2016. JLA PSP Data Management Guide. Southampton: James Lind Alliance.

McParlin C, O'Donnell A, Robson SC, Beyer F, Moloney E, Bryant A, Bradley J, Muirhead CR, Nelson-Piercy C, Newbury-Birch D et al. . 2016. Treatments for Hyperemesis Gravidarum and Nausea and Vomiting in Pregnancy: A Systematic Review. JAMA: Journal of the American Medical Association 316(13):1392-1401.

O'Donnell A, McParlin C, Robson SC, Beyer F, Moloney E, Bryant A, Bradley J, Muirhead C, Nelson-Piercy C, Newbury-Birch D et al. . 2016. Treatments for hyperemesis gravidarum and nausea and vomiting in pregnancy: a systematic review and economic assessment. Health Technol Assess 20(74):1-268.

UK Clinical Research Collaboration. Health Research Classification System [Internet]. London: UK Clinical Research Collaboration; 2009. Available from: http://www.hrcsonline.net/sites/default/files/HRCS_Document.pdf

For peer review only

For peer review only - http://bmjopen.bmj.com/site/about/guidelines.xhtml

Go straight to content.

MRC Medical Research Council

NHS Health Research Authority

Is my study research?

To print your result with title and IRAS Project ID please enter your details below:

Title of your research:

```
Hyperemesis Gravidarum Research Priority Setting Partnership
with The James Lind Alliance
```

IRAS Project ID (if available):

N/A

You selected:

- **'No'** - Are the participants in your study randomised to different groups?
- **'No'** - Does your study protocol demand changing treatment/ patient care from accepted standards for any of the patients involved?
- **'No'** - Are your findings going to be generalisable?

Your study would NOT be considered Research by the NHS.

You may still need other approvals.

Researchers requiring further advice (e.g. those not confident with the outcome of this tool) should contact their R&D office or sponsor in the first instance, or the **HRA** to discuss your study. If contacting the HRA for advice, do this by sending an outline of the project (maximum one page), summarising its purpose, methodology, type of participant and planned location as well as a copy of this results page and a summary of the aspects of the decision(s) that you need further advice on to the HRA Queries Line at **HRA.Queries@nhs.net**.

For more information please visit the **Defining Research** table.

Follow this link to start again.

Print This Page

NOTE: If using Internet Explorer please use browser print function.

About this tool **Feedback** **Contact** **Glossary**

Supplementary file 4

Data codes

Codes for types of participants

Code	Demographic question response
P	I was diagnosed with HG during pregnancy or I had HG during pregnancy but was not diagnosed
C	I cared for my partner/daughter/sister/friend when she had HG
O	My mother had HG during her pregnancy with me (offspring)
H	I am a health or social care professional caring for women with HG
ORG	I am part of an organisation representing people with HG and their families

Codes for raw questions prior to establishing indicative questions

OOS	Out of scope;<br>1 = alternative therapy<br>2 = other
C	Cause
H	Hereditability
R	Recurrence
MMA	Markers/Measurements/Assessment tool incl. clinical predictors
RF	Risk factors
DC	Disease characteristics incl. prevalence, duration, symptoms
T<br>TM<br><br><br><br><br><br><br>TG<br>TO<br>TIV<br>TN<br>TP<br>TA<br>TSO<br>TMM<br>TL<br>TT<br>TSAT	Treatment<br>Treatment - Medication;<br>1 = efficacy<br>2 = safety<br>3 = development of new ones<br>4 = combining/polypharmacy<br>5 = side effects<br>6 = administration (route/dose/timing)<br>Treatment guidelines/protocols<br>Treatments - other<br>Treatment - Intravenous fluid<br>Treatment – nutritional/nutrition<br>Treatment - Prevention<br>Treatment - Access<br>Treatment service organisation<br>Treatment – Marijuana<br>Treatment -Licensing<br>Treatment – Termination<br>Treatment - Satisfaction
S	Support<br>1 = psychological<br>2 = holistic<br>3 = family/carer
CS	Coping Strategies
CCO	Consequences, Complications, outcomes, other symptoms<br>1 = foetal risks/consequences incl. long term

For peer review only

	2 = maternal risks/consequences incl. long term<br>3 = mental health during pregnancy<br>4 = mental health after pregnancy<br>5 = quality of life<br>6 = for family
ED	Education<br>1 = of HCPs<br>2 = public/others
POST	Post HG, recovery, trauma
ECON	Economic questions
METH	Methodology specified
A	Attitudes/Beliefs/Stigma/Awareness
OTHER	Other (for further discussion with Steering Group)

For peer review only

A JAMES LIND ALLIANCE PROJECT

HYPEREMESIS GRAVIDARUM PRIORITY SETTING PARTNERSHIP

EVIDENCE CHECKING PROTOCOL

This summary sets out the approach to the evidence checking process for the Hyperemesis Gravidarum Priority Setting Partnership (HG PSP), including the specific role of the Steering Group (SG) and what will be done with the findings.

THE PURPOSE OF EVIDENCE CHECKING

Systematic reviews and guidelines will be identified and checked by Caitlin Dean (CD) to see to what extent the refined questions, generated from the raw survey data, have, or have not, been answered by previous research. Sometimes, uncertainties are expressed that can in fact be resolved with reference to existing research evidence - ie they are "unrecognised knowns" and not uncertainties. If a question about treatment effects can be answered with existing information but this is not known, it suggests that information is not being communicated effectively to those who need it. Whereas uncertainties not adequately addressed by previous research will go forward to the next stage for prioritisation.

THE PROCESS FOR EVIDENCE CHECKING

Searches

A scoping search of Embase and Medline will be conducted by an information specialist (René Spijker as part of a wider evidence mapping project running concurrently with the HG PSP. The search strategy will seek to find all papers about HG without limitations. Embase, Medline will be searched for the key terms hyperemesis gravidarum/ or (("Excessive vomiting" or (pernicious adj3 vomiting) or hyperemesis) and (gravid* or pregn* or gestation or antenatal)). Additionally, for this evidence check, searches will cover the Cochrane Database of Systematic Reviews, the Cochrane Controlled Trials Register and trip Database for Guidelines. The RCOG and ACOG guidelines will be checked for references with a suitably high level of evidence (ie. Systematic review within 10 years) as per the JLA methodology handbook.

The results will be screened and labelled as to which question number they relate to by two researchers independently.

Each question will be checked against the labelled results for systematic reviews with conclusive results which directly answer the question.

Additionally, the Cochrane Library and the RCOG and ACOG guidelines will be checked for each question for a suitably high level of evidence with a definitive recommendation/answer.

Excluding NICE

NICE guidelines will not be used for evidence checking for two reasons;

1. Statements and recommendations are not referenced in a way that can be cross checked for level of evidence.
2. The website states: *"This CKS topic is largely based on the Royal College of Obstetricians and Gynaecologists guideline: The management of nausea and vomiting of pregnancy and hyperemesis gravidarum [RCOG, 2016], the American College of Obstetricians and Gynecologists Practice Bulletin: Nausea and vomiting of pregnancy [ACOG, 2015], and review articles."* These references are already being check as stated above.

10 year inclusion

Due to the paucity of HG research and lack of funding, the SG felt that a three-year limit on SRs, as recommended by the James Lind Alliance, was too limited. Therefore, SRs within the last 10 years will be deemed acceptable.

Final outcome

Uncertainties which are not adequately addressed by previous research will be collated and recorded on a template supplied by the JLA (see Appendix A) by CD and will go onto the next stage for prioritisation.

UNKNOWN KNOWNS

Questions which are identified as already answered 'Unknown knowns', need to be translated into accessible information for the general public. CD will write a general article outlining the questions we have answers to and the answers themselves (not scientific) in lay language for dissemination to the general public via the main international charities, blogs and newsletters. Additionally, authors of guidelines such as the RCOG and NICE will be informed of the findings to ensure they are included in updates to the guidelines (if they are not already).

FURTHER INFORMATION

The JLA Guidebook provides step-by-step guidance to establishing and managing PSPs. You can find the Guidebook at www.jla.nihr.ac.uk

For peer review only - http://bmjopen.bmj.com/site/about/guidelines.xhtml

Supplementary File 6: Questions, evidence, participant categories of submissions and examples

Question rank after first prioritisation	Question code	Uncertainty	Answered?	No of refs identified from search strategy	Evidence	Total sub-missions	patients	HCPs	Carers	Off-spring	Org reps	Unkn-own	Sample of submitted questions (sic)
1	1	What causes hyperemesis gravidarum?	No	62	Various papers with new research in recent years. No definitive answer or SR yet.	282	249	28	9	31	1	14	Where does HG come from? What causes HG to begin with and why does it only affect some women? What medical studies is have been conducted to find the cause / cure for HG? Why do we get hg? What is the pathophysiology behind hyperemesis gravidarum? Why do some women get HG snd others don't? Why does one person get HG and the other don't?
11	2	What are the risk factors for developing HG and can we predict who will develop it?	No	85	Fan, L. Y.; Jacobsen, K. H. 2010 Risk factors for hyperemesis gravidarum, Current Women's Health Reviews - Volume 6, Issue 4, pp. 309-317: systematic review from 2010 identifies a few risk factors; Low pre-pregnancy weight, Helicobacter pylori infection, a history of hyperemesis gravidarum in a previous pregnancy, and carrying a female fetus but calls for further research with better definition of maternal characteristics.	96	77	21	4	12	1	7	Are there any ways to predict who will develop HG? As they learn more about HG, to learn if women could be tested thru gene testing. Is there a test being developed to determine likelihood off suffering HG? Can we use genetic testing to identify women as risk and offer preventative meds earlier? And will this lessen the severity? Is there any way of predicting if you will suffer from this?
3	3	What is the risk that HG will reoccur in a subsequent pregnancy? Does HG get progressively worse with subsequent pregnancies and what are the risk factors for recurrence?	No	8	5 papers vary from 15-81%, meta-analysis not possible.	212	191	16	10	17	0	6	What is the recurrence rate? Will I definitely have HG again in future pregnancies? Does Hyperemesis get worse with each pregnancy? What are the chances that you get HG with the following pregnancy/pregnancies? If you have HG, how likely are you to get it a second time? What are the chances of getting HG again after suffering with it during my first pregnancy? Is HG more severe dependant on partner? Are you likely to get HG again if you use sperm from a different man/donor? How much of an influence does the sperm have?
40	4	What clinical measurements and markers are most useful in assessing, diagnosing, managing and monitoring hyperemesis?	No	47	Niemeijer, M. N.; Grooten, I. J.; Vos, N.; Bais, J. M.; van der Post, J. A.; Mol, B. W.; Roseboom, T. J.; Leeflang, M. M.; Painter, R. C.; 2014 Diagnostic markers for hyperemesis gravidarum: a systematic review and metaanalysis American Journal of Obstetrics & Gynecology - Volume 211, Issue 2, pp. 150.e1-150.15: Systematic review concludes no support for the use of ketonuria in the diagnosis of HG. H	11	7	3	0	1	0	1	What anthropometric measures and nutritonal markers are most useful in assessing and monitoring hyperemesis management? (??weight, mid upper arm circumference, transferrin, amylase etc?????) What clinical indicators determine the threshold for hospital admission in women with HG? What diagnostic criteria/guidelines would provide the best treatment/outcomes? i.e. instead of waiting for significant weight loss and dehydration or “penalizing”proactive care that reduces these indicators

For peer review only - http://bmjopen.bmj.com/site/about/guidelines.xhtml

					pylori serology might be useful in specific patients.								
46	5	What objective measures can people used to know when to seek further help or hospital admission?	No	0	None	7	6	1	0	0	0	0	Self monitoring urine to know when to need help.... is this a possibility? Is there a way for the individual to self monitor how bad things are getting to be able to be proactive? How do you know when you need to go to hospital, eg what are the measures of dehydration? I remember going to hospital and being told you are not dehydrated enough for treatment following that I was reluctant to go to hospital
39	6	Can we predict the severity and duration for individual patients and which treatments they will respond to?	No	7	Some original studies identified but no systematic review or conclusive evidence	12	7	5	0	0	0	0	What factors predict severity and duration of HG in a pregnancy? Are there any clinical predictors of response to different anti-emetics in HG? Why do some HG sufferers have relief of symptoms afte 3/4 months and some are sick each day until the day they give birth? Can we predict how long HG will last? Is there any way of predicting which medications are most effective for whom?
	7	Does the sex of the foetus affect occurrence and/or severity of HG?	Yes	8	increased ratio of female to male fetus in HG pregnancies. (OR 1.27; 95% CI 1.21–1.34): MVE Veenendaal AFM van Abeelen RC Painter JAM van der Post TJ Roseboom, 2011, Consequences of hyperemesis gravidarum for offspring: a systematic review and meta-analysis. BJOG 18(11)	21	19	2	1	3	0	1	What are the chances my HG will be worse with a girl pregnancy than with a boy? Does the sex of the baby (boy/girl) make a difference in the extent of complaints of HG? Is there A difference in HG Between women who are Carrying girls vs boys?
29	8	Does Ketoanalysis have a role in the diagnosis and management of HG?	Yes and no	3	Ketoanalysis does not corrlate with severity of HG and should therefore not be used for diagnosis of HG however, this does not answer whether or not they have a role in management of HG. Niemeijer, M. N.; Grooten, I. J.; Vos, N.; Bais, J. M.; van der Post, J. A.; Mol, B. W.; Roseboom, T. J.; Leeflang, M. M.; Painter, R. C.; 2014 American Journal of Obstetrics & Gynecology - Volume 211, Issue 2, pp. 150.e1-150.15: Systematic review concludes no support for the use of ketonuria in the diagnosis of HG. Guidelines conflict but based on "good practice point/clinical experience"	21	18	3	1	1	0	0	Why do we rely on ketones to test dehydration? Can treatments be provided before you have ketones in urine instead of after? Will hospitals realise they do not have to look at ketones to diagnose dehydration in a hg patient? Why is ketosis the measure for dehydration? Can I still be dehydrated without having ketones in my urine? Why do women have to get to the point of having ketones before their condition is treated seriously?

For peer review only

18	9	What should the clinical definition of HG be and can it be diagnosed in a timely and consistent way?	No	1	Guidelines give level D evidence	56	51	3	0	7	0	4	At what point is your morning sickness classed as HG and is there different levels of HG ie Mild/Severe? How can we diagnose and treat patients quicker? Can hg be detected earlier in pregnancy? How can we identify hyperemesis earlier in pregnancy? What are the criteria of HG? How can Health Care Professional assess and diagnose HG earlier? How can HG be quickly diagnosed (and not dismissed as simply morning sickness)? My mother had it and was left to cope what is bad enough to get a disgnosis? How do I get diagnosed rather than told it's normal?
27	10	Is having HG a risk factor for requiring induction/c-section, miscarriage, other conditions/complications of pregnancy, or particular birth outcomes?	No	49	Multiple case-control and cohort studies identified. An SR may be able to answer this.	30	29	2	1	4	1	1	Whats the connection with HG en pregnancy diabetes? Could HG be related to any other sickness or disorder in my future, just as gestational diabetes is related with the chances of developing diabetes type 2 after pregancies? Are thyroid issues related to first having HG? Induction and C section rate amongst HG? Is there a link between hg and gestational diabetes? Is HG linked to 1gall bladder dysfunction? 2gestational diabetes 3 sluggish digestion 4 thyroid levels? Has there been any link between HG sufferers and delivery type? I.e. an increased chance of forceps delivery?
15	11	Does HG run in families? What is the level of risk of experiencing HG for a person with a family history of HG?	No	7	Multiple studies identified. An SR may be able to answer this.	74	63	6	8	10	0	2	Is HG heritable? If my mum had HG what are my chances of getting it? How likely is it to get HG if both mother and grandmother had it? Can anything specific mitigate the possibility of children experiencing hyperemesis themselves during future pregnancies, if their mother suffered hyperemesis? As I've had HG in my pregnancy what is the likelihood of a close relative suffering from the condition as well? E.g. sister or daughter. Because I had hyperemisis what are the chances of my daughter having this?
26	12	How do factors such as diet, nutrition, stress, tiredness and rest affect symptoms and coping? What can exacerbate symptoms and how can we manage those factors?	No	3	3 studies identified, each on different topics.	31	26	4	3	2	1	2	What is the effect of rest/tiredness on vomiting during pregnancy? Are there any triggers which can worsen hyperemesis? Are there and factors that aggravate HG? What factors (biological ans psychological) exacerbate HG? And how can these be managed during HG? Some foods increase phlegm/mucus and some foods increase throwing up. Is there a relation? Does fatigue/tiredness/ workload affect the nausea and vomiting exerienced? Do certain foodsubstances have more influence on HG?

For peer review only

For peer review only - http://bmjopen.bmj.com/site/about/guidelines.xhtml

5	13	Is HG preventable? What is the effect of preventative treatment or early intervention on the severity and duration of HG in a subsequent pregnancy?	No	1	One study which does not sufficiently answer the question	168	152	22	5	12	1	1	Is there anything the patient could have done/can do different to prevent it? What can be done to help prevent HG in those identified as at risk, I e those who have had it in previous pregnancy. What factors determine duration - do certain treatments or early intervention affect duration or is it determined by the underlying cause? What is the best way to get HG under control ASAP and limit the mental effects to the patient? If you start taking medication early on in pregnancy will this lessen symptom severity overall?
20	14	What are the disease characteristics of HG? (Prevalence, onset, duration, symptoms, peaks?)	No (and yes)	14	Some aspects answered such as prevalence, but other aspects such as onset, duration, symptoms and peaks not answered. Prevalence SR and meta analysis concluded: The prevalence of HG was 1.1% (CI95%:0.8%-1.3%), with a range of 0.3%-3.6%. Almost 70% of women worldwide experience NVP, but reported rates vary widely. HG, the most severe form, affects 1.1%. Ref: Einarson TR, Piwko C, Koren G. Quantifying the global rates of nausea and vomiting of pregnancy: a meta analysis. Journal of population therapeutics and clinical pharmacology = Journal de la therapeutique des populations et de la pharamcologie clinique. 2013;20(2):e171-83.	40	35	8	2	1	1	0	Numbers of how often HG occurs, are there any differences in different countries / parts of the world? Are there differences in the rate of HG across ethnic groups? How long is this likely to last? What is the chance of HG easing and then returning ? While suffering from H G I tend to be spitting every few seconds, can't swallow any of my saliva (spit cup necessary) any research on how long this spitting last's for? What kind of statistics are there regarding Hg? (Recurrence, who gets it, how long it will last...) Are there certain weeks when hormones always surge to make HG worse? What are the chances that this feeling will last the entire pregnancy? At what week does HG usually improve a bit? How common is it to have HG the whole pregnancy? What is the incidence of HG continueing after the first trimester and for how long does it continue?
23	15	How can symptoms of HG, other than vomiting, be effectively treated? For example, the nausea, excessive saliva, extreme sense of smell and fatigue.	No	5	Some prevalance studies on ptyalism (excessive salivation) identified. No discussion of other symptoms in guidelines	35	34	2	2	3	0	0	Is there an effective treatment for ptyalism?Is there anything to reduce the excess saliva that comes hand in hand with HG?One of the most distressing symptoms of HG for many women is the heightened sense of smell. Is there a medical way to target that symptom in particular?The sickness and nausea is obviously a big part of HG. But how do we treat the less talked about effects including extreme tiredness, no energy, headaches, dizziness, breathlessness, confusion, being unable to stand/endure movement or motion/listen to conversations/music without feeling like you are going to be sick or faint? How can the other symptoms be reduced (i.e. smell sensitivity, excessive saliva, extreme fatigue, recurring UTIs). Is there a way to control the extreme sensitivity to smell, which causes so much agony? Is there anything that can be done to dull the extremely heightened sense of smell that a lot of HG patients suffer with? As I honestly think it would have helped with the severity of the constant nausea in my case.

For peer review only

2	16	What are relative efficacies of the current medications and treatment options available? What are the optimal dose, route, timing and combination of the medications and what are the related side effects?	No	58	Cochrane review concludes: there is little high-quality and consistent evidence supporting any one intervention. Ref: Boelig RC, Barton SJ, Saccone G, Kelly AJ, Edwards SJ, Berghella V. Interventions for treating hyperemesis gravidarum. The Cochrane database of systematic reviews. 2016;5:DOI: 10.1002/14651858.CD010607.pub2. HTA SR found: there is little on the effectiveness of treatments in more severe NVP/HG. Ref: O'Donnell A, McParlin C, Robson SC, Beyer F, Moloney E, Bryant A, et al. Treatments for hyperemesis gravidarum and nausea and vomiting in pregnancy: a systematic review and economic assessment. Health Technol Assess. 2016;20(74):1-268	253	195	71	11	19	5	8	What are the options for treating it? What is the best dosage of x (insert any of treatments) for most effective results for most women? What are all the options available to women who experience HG? My doctor was very dismissive. What are the recommended first, second and third line anti-emetic drugs in HG? When to switch over to the first line to second line, and so on? Which is the highest dose of Cariban? Best regime. Why do doctors not readily prescribe combinations of anti-emetics when patient's symptoms appear unimproved with one anti-emetic. For example cyclizine and metoclopramide?
24	17	How can we most effectively manage HG? What clinical support measure is most important to women who have had hyperemesis and what did they find most beneficial? eg medical management, pharmaceutical review, nutrition support, rehydration, psychological support	No	13	Some trials, case-control and case studies for various support measures. No SR, no large well conducted trials.	34	27	6	3	5	0	1	What's the best way to manage HG? What clinical support measure is most important to women who have had hyperemesis and what did they find most beneficial? eg medical management, pharmaceutical review, nutrition support, rehydration, psychological support. What are the best treatment methods for Hyperemisis Gravidarum? What is the treatment recommendations? (Including medicines, food intake, intravenous therapy, psychological help...) How can we use the medical intervention available to us to make this disease more bearable for mothers who suffer?
17	18	Can we find a cure? What novel or new treatments are being developed/tested/used elsewhere which could have a curative effect and to address all the symptoms of HG rather than just the vomiting?	No	9	Some small and novel trials identified for gabapentin, clonidine, mitrazepam, marijuana and other therapies.	66	52	7	3	6	2	7	Is there a cure? Will there ever be a cure for this 'HG'? is there not a surgical way or medical way to eradicate this reaction? By what treatment can HG be totally cured? This means de vomitting, the nausea, lack of feeling hungry, etc. Given the 2 genes (contributing to appetite and placenta) which were recently found to be connected with the root causes of Hyperemesis Gravidarum, how can one reduce his / her chances of acquiring this sickness or cure it in an earlier stage than 20 weeks plus or the entire pregnancy? Please try to find a cure!!

For peer review only

For peer review only - http://bmjopen.bmj.com/site/about/guidelines.xhtml

4	19	What is the effect of HG on mental health during pregnancy? What is the efficacy of psychotherapy on symptom management/pregnancy outcomes/quality of life? How can women access psychosupportive services during pregnancy?	Yes and No	28	Systematic review and meta-analysis have shown a significantly increased frequency of depression and anxiety in women with HG. Ref: Mitchell-Jones N, Gallos I, Farren J, Tobias A, Bottomley C, Bourne T. Psychological morbidity associated with hyperemesis gravidarum: a systematic review and meta-analysis. Bjog. 2017;124(1):20-30. For interventions, various studies have assessed interventions, SR may be possible but question is not currently answered.	188	172	20	7	25	2	6	How does the mental component of HG (desperation, exhaustion, feeling that 'I can't do this for another eight months') influence the patients physical wellbeing and how do we include mental health care in the treatment? Can mental health support be provided at home for those who are unable to travel alone? How do we support women with HG before, during and after pregancy psychologicly? What mental health support is available for women and how can we improve access to this service for women who struggle to leave the house when suffering with HG? What support is available for the psychological repercussions of HG? Comparison between conventional medical treatment and psychotherapy for hyperemesis gravidarum. What are the barriers to women accessing counselling for HG? Suicidal thoughts and HG. How can this be better supported by the the community midwife and mental health team?
33	20	How can the condition be effectively managed in the community to prevent lengthy hospital admissions?	No	7	Some studies comparing ambulatory to inpatient care.	17	13	6	0	1	0	1	What is the best management or treatment to get women with HG out of the hospital and back home rather than staying in hospital? What can be done to keep HG sufferers home instead of in hospital? Can hyperemesis patients prevent themselves getting into a situation where they need to be admitted to hospital by having regular IV drips, perhaps day appointments for this or community midwifes administer this as a method of prevention/management of the condition? Are women who are car d for at home with HG more or less likely to suffer from post natal mental health issues?
25	21	Do clinical treatment guidelines for HG improve management and outcomes? And if so how can guidelines be developed and implemented nationally (where none exist) and internationally for hospital and community settings? What should be included in guidelines?	No	6	Some audits of local guidelines highlight inconsistent treatment. Effectiveness of national RCOG and ACOG guidelines have not been evaluated.	33	28	6	0	1	0	3	Would there be a benefit to developing a national best practice guidance for managing HG? can there be one HG protocol for every healthcare provider? Make a list of available medication along with safe dosage available to all medical professionals, as all too often treatment/medication given varies between hospitals, due to lack of information. Is it possible to create a worldwide guide so docters treat everybody the same. There is so much difference between country's but even between hospitals and docters.
13	22	What are the barriers to accessing treatments/services and how can we reduce them to improve access?	No	4	Four small studies/case studies identifying barriers to accessing treatment.	95	86	11	5	7	2	1	What treatment is available, how effective is each treatment and how do I access it? When partner is to ill to communicate how do I get her the best medication or know where to go for help? How can I initiate the process for home care with my insurance if homebound with HG? How can I access IV hydration at home? How can we support medical/nursing teams to access specialist mental

For peer review only

													health support for HG sufferers/families when NHS budgets are constantly being cut?
30	23	What self-management and coping strategies and treatments do people with HG find most helpful?	No	0	None identified	20	17	2	2	0	0	0	What can make women with HG feel better? I tried many thing nutricion , medication , many sleep but nothing worked out, what worked out for the best for other woman ?
34	24	What percentage of people with HG are satisfied with the care and treatment they received? How can satisfaction be increased and how can healthcare professionals best support people with HG?	No	0	None identified	17	13	3	0	2	0	1	How can we help in clinic? What percentage of women are satisfied with the treatment they receive for HG? How can quality of care be improved for women with HG? Were the midwives and doctors helpful whilst you were suffering? Which support and treatment options do women attribute to helping them have a positive pregnancy experience (eg a care plan, continuous care from a midwife, early diagnosis and treatment, help to make informed choice regarding treatment options etc)?
37	25	How can dehydration be prevented/managed without an IV drip?	No	0	None identified	13	10	2	0	1	0	1	What am I supposed to do when I can't keep liquid down to stay hydrated? Can the priority shift from treating dehydration to preventing dehydration? How dangerous is getting towards dehydration for longer periods or more often for the vascular system of the uterus and effects on the foetus?
10	26	What healthcare services exist and how can they collaborate and be organised to better identify, treat and support women with HG? For example, do services such as outpatient clinics or IV at home, improve outcomes and reduce the physical/mental burden of the condition?	No	18	Some audits, case-control studies and RCTs identify services such as Home Healthcare and outpatient services in specific areas of individual countries. SR of outpatient verse inpatient outcomes may be possible.	97	78	21	4	12	3	2	How could the healthcare system be better equipped to identify and treat hg? which reproducible model of management (inpatient day unit/ via a&e) has the best outcomes? How can consultant-led hyperemesis specialty clinics in maternity services improve co-ordination of care and outcomes for women? Do you think there is better scope for community treatment of Hyperemisis Gravidarum to improve women's lifestyles and mental health? Do women prefer inpatient or outpatient care for hyperemesis? What is the cost/impact of HG management in acute settings vs a community based model?
14	27	What other services are available/could be developed to support families affected by HG? And how can access be enabled to services which currently exist	No	2	Two studies suggest counselling and martial support may be helpful	78	70	12	6	5	0	0	Can mental health support be provided at home for those who are unable to travel alone? Is there any association of volunteers to help or support the expectant mother, husband and or older siblings in everyday life? E.g. picking up medication for mom, prepare food, taking care of kids...
19	28	How long does it take people to recover from HG? What post-pregnancy is care available (or could be developed) for people who have suffered HG during pregnancy and what is its impact on recovery?	No	2	Two studies (one survey, one case-control) looking at aftereffects and recovery identified.	50	46	1	4	9	1	1	How long does the average HG sufferer take to 'get over' the trauma of her pregnancy? Do some never get over it? What aftercare can be made readily available for women who have had hg during pregnancy? How does postpartum care need to be organised to shorten recovery?

For peer review only

47	29	Do specific specialist healthcare professional roles for conditions such as HG improve outcomes? How can such roles be developed for midwives/nurses/doctors?	No	0	None identified	7	6	2	0	1	0	0	Will there be special hg physicians in the future? Can we look into a community midwife specialising in hyperemsis? It would be nice if there is a special HG clinic or team that is specialized in HG. Would that be possible in the near future? Which doctor (gynecologist)is the best, most experienced doctor for treating Hyperemesis gravidarum in Europe?
35	30	How does HG treatment/management vary by country (and regions within countries) and how do the variations effect outcomes?	No	0	Various types of services reported in studies and guidelines from around the world. No research addressing question.	16	14	1	0	1	0	1	Why is the medical treatment different in an other country? Here in the Netherlands you can only get emesafene and sometimes metoclopramide for max 5 days. Why is the treatment in the uk not as efficient as that of the us? (I.e home care, pick lines). Why is care so different in so many areas ? Why do the treatments vary so much per hospital/country/doctor
44	31	What additional checks, tests and medical reviews should people with HG be offered during pregnancy?	No	17	Various tests proposed/highlighted in case reports and case-control studies. SR may be helpful to address.	8	8	1	0	3	0	0	Should you be seen more by consultant when pregnant with HG? How often should a doctor review the patient's condition and treatments? Why don't women with HG get more regular or routine appointments to check the healthy development of the foetus? Why are HG sufferers not offered earlier blood tests to monitor iron levels etc? when do you consider further investigations like imaging/endoscopy in cases of persistent hyperemesis beyond 16 weeks of pregnancy? why aren't women who suffer from HG monitored more closely to make sure baby's growth is going well? When you vomit many times a day for months, why aren’t electrolyte levels checked?
8	32	Do negative stigmas, attitudes and beliefs surrounding HG affect the quality/consistency of care provided on individual healthcare professional and institutional levels? If so, what is the impact of such stigmas and how can they be addressed?	No	1	A number of studies identified that found persistent stigmatisation of HG among healthcare professionals in the UK. Qualitative SR may be possible	112	108	9	4	10	3	0	Why does the quality/consitency of care, in HG patients, differ so much between medical professionals? Why is the care so variable - I.e from one gp to the next? Why is it so difficult to access rapid and effective treatment? Having to fight for anti emetics and rehydration and being treated like a hysterical addict by doctors adds to the trauma of hg. Why is it so difficult to access the right medication and treatment in some areas? How can you get taken seriously by you gp when you report this, and not just brushed off as being silly? How can I explain what I’m feeling to my doctor in a way that will help me be taken seriously? What are the consequences for the patient when professionals say to them that it’s a psychological issue, even if they still treat the patient physically?

For peer review only

51	33	How many people terminate their pregnancy due to HG symptoms, lack of access to treatment or other related factors? What are their experiences and how can they be supported?	No	1	Some studies identifying rate of termination but no conclusive evidence. SR may be possible.	6	6	1	1	0	1	0	How often do women terminate wanted pregnancies because the hg is too difficult? How many terminations are carried out because of lack of access to treatment for HG
53	34	When is it appropriate to induce labour early and/or conduct elective c-section due to third trimester HG?	No	0	No papers identified and no mention of induction/elective section in guidelines	4	3	0	0	0	0	1	Can I be induced at 36 weeks because of the severity of hg? If an unborn child can be delivered-healthy as early as 36 (?) weeks, why are doctors not relieving HG mothers of their symptoms who are still suffering near the end of their third trimester. If the woman with Hyperemesis Gravidarum suffers from the condition throughout the whole pregnancy and is severely anemic and weak, is elective Cesarean section recommended, that is, is that woman in greater risk of having complications enduring vaginal birth?
28	35	How can people with a history of, or significant risk factors for HG be supported to plan for a pregnancy and does such planning improve outcomes? What should a pre-pregnancy plan contain?	No	0	RCOG guidelines suggest possible benefit to prophylactic treatment but evidence is poor. No evidence for pre-pregnancy care plan.	27	24	2	2	5	0	1	Planning for a second pregnancy can be very difficult. How can barriers be broken down for more women before pregnancy ? How can we better support women who are terrified to have another child?
31	36	What is the role of vitamin supplementation during HG, which vitamins are important, how should they be administered, and do they effect outcomes?	No	36	Multiple articles addressing various vitamins particularly Vit K and B1. SR may be possible.	20	17	3	2	3	0	1	It is safe to take vitamins if I have HG? I was unable to swallow pregnancy tablets and was malnourished through being unable to eat or drink for 6 months. I received one dose of vitamins through a drip in this time. Why was I not given these regularly.
60	37	How can appointments and clinics within all possible settings (home, GP, hospital etc) be made more 'HG friendly'? (ie. Reducing sensory stimulations, smells, noise, location, access to toilet/sick bowls etc)	No	0	No studies identified	2	1	1	0	0	0	0	How can appointments and clinics within all possible settings (home, GP, hospital etc) be made more accessible and 'HG friendly'?
38	38	What are the barriers to taking/prescribing medication for HG? How can the risk and benefits of HG and its treatments be better communicated to support informed decision making and consent to treatment?	No	1	Some barriers identified but no conclusive studies within HG literature. A wider search regarding medication use in pregnancy may yield more insight which is applicable to HG?	13	11	2	0	2	0	1	The biggest worry is taking medication and any impact that may have on your baby, clear advice and information needs to be provided prior to taking any medication. I was only offered stemitil which didn't help and only learned afterwards other medication was available.
54	39	What are the barriers to medication testing and licensing for use during pregnancy and how do we try to overcome those barriers?	No	0	No results within HG literature but a wider search regarding general medication testing in pregnancy may yield more insight which is applicable to HG?	4	4	0	0	0	0	0	Need of RCTs whene it comes to effect of medications? Why are there so few medications licensed and well-tested for use for HG during pregnancy? Has odansetron become licensed for use in pregnancy yet? Can it become standard for GP's to prescribe nationwide?

56	40	Do personalised care plans reduce suffering and improve outcomes for people?	No	3	One RCT assessed difference between patients receiving individual holistic care package and controls receiving "normal care". No statistical difference was found. Further studies required.	3	2	1	0	1	0	0	How can action plans be written up to ensure dehydration, hospitalisation are reduced? Such as day visits for fluids, and consult led care
6	41	What is the currently level of knowledge about HG and its treatments among healthcare professionals (particularly GPs)? How can effective education for healthcare professionals be designed and delivered to improve the general knowledge and awareness of HG among HCPs?	No	5	Some studies/audits have tried to assess local current knowledge but do not address how education can be improved or delivered.	148	138	9	8	22	2	4	I think raising awareness in medical professionals is the best way to support women with HG. How can we do this? Could all hospitals and medical professionals be better educated as to how they deal with HG? Is there a process to follow? considering complications, general state, psychological state, evolution of the pregnancy, effects on patient's day to day life and for patient's surroundings: are there significant differences between patients treated by well informed professionals and patients treated by professionals who don't have particular knowledge of HG ?
32	42	What is the most effective IV rehydration regime: which solution in what quantity over what time period and how frequently? Does regular rehydration improve symptoms/outcomes/quality of life?	No	5	Lack of clear studies, current recommendations in guidelines from low level evidence.	19	12	7	1	0	0	0	Is there a difference between different electrolyte infusions? What is the best IV fluid regimen in women with HG? How effective is rapid rehydration as an outpatient treatment?
36	43	How can weight loss/malnutrition from HG be effectively managed/reduced? Do nutritional therapies such as NG tubes, TPN, PEG feeding improve outcomes?	No	15	Some studies and case reports of enteral feeding for HG with mixed results. Lack of evidence for nutritional therapies in general. ACOG guidelines vague on enteral feeding, discourages parenteral feeding with low level evidence references but lacks alternatives to managing weight loss and malnutrition	14	10	4	0	0	0	0	Whicht treatment to reduce the HG and minimize the weightloss? Is it nutrition or IV for example? What role does IV nutrition play in preventing long term health impacts for HG babies? What role does IV nutrition play in preventing premature birth?
57	44	What are the risks and benefits to using Peripherally Inserted Central Catheters (PICC) lines and central lines for people with HG requiring regular IV therapy?	No	2	ACOG and RCOG references a number of low-level evidence sources highlighting risks associated with PICC lines in pregnancy.	3	3	0	0	0	0	0	If I am going to emerge 3 or 4 times a week for IV fluids and they have trouble finding veins because of dehydration why can't they put in a port or picc earlier why must we suffer so long and get yelled at for a shitty veins ?
61	45	How should treatment vary throughout pregnancy according to physiological changes and metabolic requirements? Ie. Should doses increase later in pregnancy, are some medications less suitable close to delivery	No	0	No studies identified	2	1	0	1	0	0	0	Does choice of best medication to treat HG change with gestation?

For peer review only

For peer review only - http://bmjopen.bmj.com/site/about/guidelines.xhtml

55	46	How does HG impact on a person's (and their family's) quality of life? How does quality and efficacy of treatment impact that effect?	Yes and No	7	Negative effects identified in a qualitative systematic review ref: Dean C, Bannigan K, Marsden J. Reviewing the effect of hyperemesis gravidarum on women's lives and mental health. British Journal of Midwifery. 2018;26(2): Plus further studies since then add to evidence base. However, no evidence identified on how quality and efficacy of treatments mitigate or exacerbate the effect.	4	3	2	0	0	2	0	What is the range of difference in quality of life for patients as linked to the medicines they are given?
58	47	Why are some cases of HG unresponsive to all antiemetics and how can we treat such cases?	No	9	Cause of unresponsiveness not addressed in literature. Various novel therapies, enteral/parenteral feeding and termination proposed for unresponsive cases in a small number of papers and the two guidelines.	3	3	0	0	0	0	0	Why in some cases is it not possible to control the sickness and vomiting? Why doesn't medication work (good enough)? Why do different medications or combinations of medications work (or not work) for different patients? Why don't pills or medications work against the hg effects?
12	48	What are the immediate and long-term effects of the various medications/treatments on the developing foetus throughout the various stages of pregnancy and in varying doses or combinations of treatments?	No	20	Various studies and reviews of individual medications for particular exposure periods. For example, SR found: "first trimester ondansetron exposure is not associated with an overall statistically significant increased risk of congenital malformations or cardiovascular defects." ref: Balayla, J.; D'Alton, M. E. 2016 First trimester ondansetron exposure and the risk of major congenital malformations and heart defects: A systematic review and meta-analysis. American Journal of Obstetrics and Gynecology - Volume 1, Issue 0, pp. S177. This doesn't answer the question on whether ondansetron is safe throughout pregnancy, third trimester or in what doses or in combination with other medication	96	80	19	2	9	0	3	Do any of the medicines harm the baby? Does the type, dose and duration of medication have different effects on the baby depending on the time of the pregnancy the medication is used. For example, does ondansetron have a different (potential) effect on the baby if this is taken in the first trimester vs the last trimester? Is this drugs cocktail really the best thing for baby?
45	49	What dietic service support is currently available to people with HG within maternity services? What impact does dietic care have on clinical outcomes and quality of life?	No	0	No studies identified, Guidelines suggest dietician involvement based on expert opinion	8	4	5	0	0	0	0	What is women's experience of dietetic support in maternity services? What is women's experience of dietetic support in maternity services? What impact does dietetic care have on clinical outcomes and QoL for women with hyperemesis? Is there any improvement in clinical outcomes in those who recieve dietetic input versus those who do not?

For peer review only

21	50	What are the nutritional requirements of the 1st, 2nd and 3rd trimesters and how can people with HG achieve these goals? Ie. Oral supplements, fortifying food, dietary measures	No	1	None identified within HG specific literature but a search within wider pregnancy literature may identify evidence relevant to this question.	38	32	5	4	6	0	0	Is there benefit in providing hyperemesis patients with oral nutritional supplements? Which foods are best to eat when suffering from HG? What is the best way to gain and retain vital nutrients during HG? What dietary advice is best for a HG-patient?
63	51	Does physiotherapy have a role to play in HG management?	No	0	No studies identified. Guidelines suggest multidisciplinary approach	1	1	0	0	0	0	0	Would having some Physiotherapy possibly reduce the risk of blood clots and help mental health Of those suffering HG?
22	52	What are the wider biopsychosocial impacts of HG on partners, families, older children, relationships, work and finances? And how can families be supported?	No	5	some studies address wider family impacts. SR maybe possible. No studies on support for families	37	34	1	0	4	2	2	What is the impact of HG on the woman's partner/ family (e.g. having to take time off work, becoming a carer, feeling hopeless and helpless) and how does HG change relationships
7	53	What are the immediate and long term effects of HG (including malnutrition and dehydration, stress) on the developing foetus?	Yes and No	56	Some immediate effects identified in systematic review but long-term consequences require further study: Veenendaal MV, van Abeelen AF, Painter RC, van der Post JA, Roseboom TJ. Consequences of hyperemesis gravidarum for offspring: a systematic review and meta-analysis. Bjog. 2011;118(11):1302-13.	130	112	25	3	8	1	3	What are the effects/consequences of HG for the baby and the mother in long term? What are the risks to the fetus when a woman has Hyperemisis Gravidarum? What are the long term effects of a HG pregnancy on the health of a child? (Impact of medication but also, is there any impact on the child anyway without meds e.g. long term growth, IQ, physical health, mental health etc)
9	54	What are the immediate and long term, physical, mental and social consequences and complications of HG (including malnutrition and dehydration) on the pregnant person's body? (ie. Metabolic impact, DVT, depression, effects of dehydration)	No	136	Psychological morbidity has been demonstrated with systematic review: Mitchell-Jones, N., et al. (2017). "Psychological morbidity associated with hyperemesis gravidarum: a systematic review and meta-analysis." Bjog-an International Journal of Obstetrics and Gynaecology 124(1): 20-30. A myriad of other consequences and complications have been identified in the literature but no systematic review.	110	95	12	5	12	2	6	What are the most common complications of HG? How can we raise the risk of embolism, so women know when to seek help? What are the longterm effects of HG for mother and baby? Metabolic impact of hyperemesis during and after pregnancy? What are the long tern effects of HG (i.e. up to 5 years after the pregnancy)? Is HG linked to higher rates of postnatal depression, family breakdown, and decisions not to have any other children? How many HG sufferers had to stop working prematurely?
41	55	Does HG effect breastfeeding or early bonding and attachment with the baby?	No	3	Some studies looking at attachment and bonding with conflicting results. Not enough for SR. Original research required.	11	10	1	1	1	0	0	What is the impact of HG on early bonding and attachment with baby? How does HG affect breastfeeding? How does the experience of HG affect subsequent bonding and attachment between the mother and baby? Can you breastfeed through HG (i.e. breastfeed an older child -- I did but couldn't get any support or find anyone else who had done it)? Are women with HG less likely to breastfeed?

For peer review only

42	56	Does HG impact on people's reproductive choices and/or limit families? Does this have an effect on family wellbeing and mental health?	No	2	Mixed results from a couple of heterogeneous studies SR not possible yet, research needed	10	9	3	0	0	0	0	How does HG affect the likelihood of a family choosing to have another baby? Do you feel as though your experience with HG was limiting/ debilitating enough to make you not want to have another child, as opposed to if you hadn't suffered HG during your pregnancy? was HG a reason not to get any more children? Did HG stop you having further pregnancies?
48	57	How should people with comorbidities be managed while suffering HG, for example, diabetes, epilepsy, mental health conditions?	No	21	Various case reports of a variety of comorbidities with HG and a cohort study looking at diabetes. Further research needed. An SR on the case reports of gastric bands/bypass in relation to HG may be possible.	7	5	3	0	0	0	0	How do I maintain adequate blood sugar control as a patient with type 1 diabetes and hg? How can we best handle gestational diabetes when HG impacts what and when and if you can eat? If a type 1 diabetic woman gets HG what should she do? How does Syndrome Gilbert affect pregnant woman, especially one that suffers from Hyperemesis Gravidarum? How does HG effect women with current or past eating disorders? As someone that already suffers with mental health problems is there a chance hg has made things worse ?
16	58	How can awareness of the condition be raised among the public/employers/media etc? How does the public perception of the condition affect people suffering HG?	No	0	No studies identified	67	59	3	8	7	0	4	What do employers need to know in order to be able to support women with HG? The general public should be made aware of this condition - any plans to publicise this illness? Many people do not understand the severity of HG. How can it be explained to family members and employers? How can we all make the world more aware of what HG is and the horrific issues behind it?
49	59	What is the total economic cost to the individual, their family, the health system, and society when a person has HG? How do differing models of care alter this?	No	8	An SR which attempted economic analysis of treatments for HG/NVP found" The economic analysis was limited by lack of effectiveness data", Further research required. Ref: O'Donnell A, McParlin C, Robson SC, Beyer F, Moloney E, Bryant A, et al. Treatments for hyperemesis gravidarum and nausea and vomiting in pregnancy: a systematic review and economic assessment. Health Technol Assess. 2016;20(74):1-268	7	4	4	0	0	1	0	What is the total economic cost to the individual, her family, the health system and society when a patient has hyperemesis gravidarum? Explore economic burden of severe NVP/hyperemesis in different countries (direct + indirect cost). What is the cost/impact of HG management in acute settings vs a community based model. What is the estimated annual cost to the national economy of working-days lost due to HG and related conditions?
50	60	How can HG research be improved and stimulated? Which treatment outcomes are meaningful to patients, what definition of HG can be used for research, what light can qualitative research shed on woman's experiences of HG, can a database of HG offspring be established, How can RCTs	No	5	Problems with definition and outcomes identified in Systematic Review which demonstrates need for consensus on these for research: Koot, M. H., et al. (2018). "Variation in hyperemesis gravidarum definition and outcome reporting in randomised clinical trials: a systematic review." Bjog-an International Journal of Obstetrics and Gynaecology 125(12): 1514-1521.	7	2	5	0	0	0	0	Which treatment outcomes are meaningful to HG patients? Can we agree on an international definition of HG? women's experiences of severe HG qualitative research? Are the children born to HG sufferers put on any sort of database to try and collate some sort of information for future medical survey/information reference? I have a more general question, methodological. Which (Medical) interventions improve the relevant outcomes. I want to see rcts! No more or much less observAtional

For peer review only

		of medical treatments be encouraged and supported?											
59	61	What other diagnosis should be considered, and at which points in pregnancy/disease stage?	No	70	Many case reports of differential diagnoses at varying points in pregnancy, SR would be helpful. Guidelines list various possible differential diagnosis based on low quality evidence and expert opinion.	3	2	1	0	0	0	0	when do you consider further investigations like imaging/endoscopy in cases of persistent hyperemesis beyond 16 weeks of pregnancy
62	62	Would an alternative name/classification for hyperemesis gravidarum better reflect the scope of the condition and improve awareness/reduce stigma?	No	0	No evidence identified. It may be worth looking at literature from other condition which have been renamed to assess potential impact of such work.	2	1	1	0	0	0	1	What can we rename HG which reflects that some women don't have severe vomiting but severe nausea which leads to same outcomes
64	63	What are people's rights to treatment and how can people with HG be enabled to exercise their rights?	No	0	No literature identified.	1	1	0	0	0	0	0	What are my rights if GP is refusing to offer medicines?
52	64	What is the mode of action for the various medications?	No	0	No specific reports within the HG literature identified. Searches on specific medications within pharmacology literature may yield better results.	5	4	1	0	0	0	0	What is the action of the various medications prescribed? I've seen caraban used for HG how does this mechanism work? Why is HG better controlled on a combination of medicines rather than just one?
43	65	What employment rights do people with HG have and what financial support is available to them?	No	0	No literature identified in relation to HG, general pregnancy literature searches per country may yield more results	9	8	0	1	1	0	0	
65	66	Are there survival/evolutionary benefits to HG?	No	3	Some studies proposing theories, no SR identified.	1	0	1	0	0	0	0	Are there survival/evolutionary benefits to HG?

For peer review only
